# Anion Recognition by Neutral and Cationic Iodotriazole Halogen Bonding Scaffolds

**DOI:** 10.3390/molecules25040798

**Published:** 2020-02-12

**Authors:** Iñigo Iribarren, Goar Sánchez-Sanz, Cristina Trujillo

**Affiliations:** 1 Trinity Biomedical Sciences Institute, School of Chemistry, The University of Dublin, Trinity College, D02 R590 Dublin 2, Ireland; iribarri@tcd.ie; 2 Irish Centre of High-End Computing, Grand Canal Quay, Dublin 2, Ireland & School of Chemistry, University College Dublin, Belfield, D02 HP83 Dublin 4, Ireland; goar.sanchez@ichec.ie

**Keywords:** halogen bond, anion recognition, DFT study

## Abstract

A computational study of the iodide discrimination by different neutral and cationic iodotriazole halogen bonding hosts was carried out by means of Density Functional Theory. The importance of the size of the scaffold was highlighted and its impact observed in the binding energies and intermolecular X⋯I distances. Larger scaffolds were found to reduce the electronic repulsion and increase the overlap between the halide electron lone pair and the corresponding I-C antibonding orbital, increasing the halogen bonding interactions. Additionally, the planarity plays an important role within the interaction, and can be tuned using hydroxyl to perform intramolecular hydrogen bonds (IMHB) between the scaffold and the halogen atoms. Structures with IMHB exhibit stronger halogen bond interactions, as evidenced by the shorter intramolecular distances, larger electron density values at the bond critical point and more negative binding energies.

## 1. Introduction

Interest in anion recognition and anion transporters, particularly in supramolecular chemistry, has increased in the last two decades. [1,2,3,4,5] Different varieties of anions play a fundamental role in chemical, biological, medical, industrial, and environmental processes. However, the field concerning anion transporters has suffered from a slower development in comparison to the cation analogous field. This is mainly because of the intrinsic properties of anions, presenting poorly defined coordination preferences in contrast with the parallel coordination found for cations. In order to address this anion-binding conundrum, halogen bonding (XB) has emerged as an attractive noncovalent interaction with tuneable features which make it a promising approach by which to enhance anion transportation. Commonly, there is a wide range of available noncovalent interactions which can be used within the ion transport field to trap or stabilise anion-host complexes such as hydrogen bonds [6] or chalcogen bonds [7], anion-π etc. Hydrogen bonding interactions are, traditionally, the most prevalent noncovalent interaction found in anion recognition, but some works point to the use of a scaffold with chalcogen atoms (S) as potential anion transporters through tuneable chalcogen interactions between halides and sulphur [7,8]. On the other hand, halogen bonds, which are often described as analogous to hydrogen bonds, present comparable binding strength and similar directionality (at least 175° in the gas phase). Scheiner reported theoretical anion transporter scaffolds establishing not only chalcogen bonds, but also tetrel, pnicogen and halogen bonds [2,3,4].

A halogen-based scaffold capacity to behave as a good anion transporter is also highlighted in the literature, not only from an experimental perspective, but also based upon a theoretical approach [9,10,11,12]. Those scaffolds establish halogen bonds with the anions due to the existence of a σ-hole on the halogen atom, described by Politzer et al. [13,14,15]. This well-known “σ-hole” accounts for the electron-deficient outer lobe of a *p* orbital involved in forming a covalent bond. It is also well-established that the strength of noncovalent interactions, particularly halogen bonds, can be tuned by a) varying the nature of the halogen donor atom (Cl, Br, I), and b) with the electron-withdrawing capacity of the atoms/group covalently bonded to the halogen atom. Halogen atoms down a group, which are more polarizable and less electronegative, are more likely to present deeper σ-holes and therefore facilitate the formation of halogen bonds with anions. Among the halogens, only the heavier elements (Br and I) present the most effective σ-hole.

**Figure 1:**
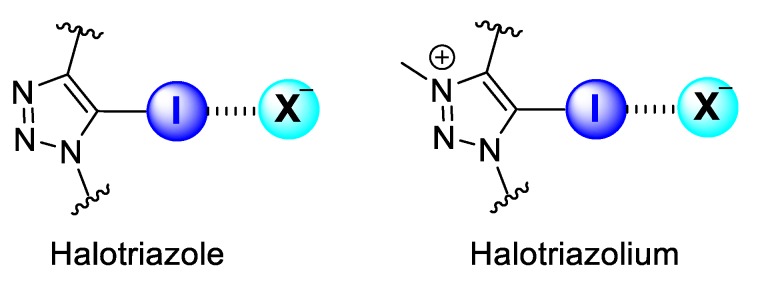
Common neutral (halotriazole) and cationic (halotriazolium) XB motifs for anion binding.

Halogen-bonding host molecules for anion recognition contain XB-donor motifs, which present a heavy halogen donor atom covalently bonded to a highly electron-deficient group. These factors account for the dominance of aromatic XB-donor groups substituted on aromatic motifs for anion binding over aliphatic scaffolds, as the latter is often susceptible to nucleophilic substitution in the presence of anions. Two of the most common neutral and cationic XB motifs that fulfil these criteria in general are collected in Figure 1.

Herein, in this work, neutral and cationic XB-donor motifs are studied considering the receptor design features required for optimizing XB-anion binding strength. The anion recognition properties of XB-anion receptors of varying structural complexities are discussed, and optimization of XB-anion binding geometries is highlighted. It is worth noting that experimental work on those anion transporters has been carried out in different solvent environments. It is well-known that gas-phase calculations can be greatly affected by the inclusion of the solvent. Noncovalent interactions such as chalcogen or halogen bonds, particularly in charged systems where the charges are shielded by the solvent, can be very affected or compete with other interactions due to the presence of a solvent [9], even when using raw or very approximative models [7,8]. For this reason, all the structures were obtained under solvent conditions mimicking the experimental conditions reported.

## 2. Results

**Figure 2:**
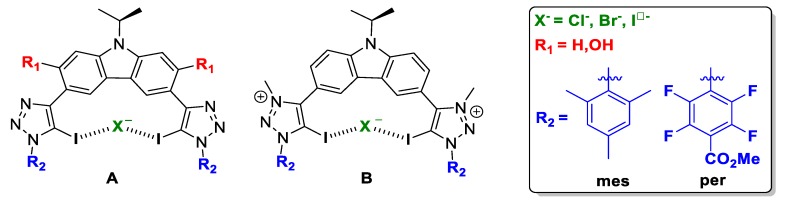
Schematic representation of the compound subject to study: (**A**) neutral XB donor motif and (**B**) cationic XB donor motif.

In order to explore a wide range of XB donor motifs, two scaffolds were considered: firstly bis-iodotriazole carbazole-based XB donor motif (Figure 2), and secondly bis-iodotriazole pyridinium-based XB donor motif (Figure 5). In both cases, the neutral and cationic XB-donor groups, halotriazole and halotriazolium respectively, were taken into account.

### 2.1. Bis-Iodotriazole Carbazole-Based XB Donor Motif

It has been experimentally shown by Schubert et al. [12] that bidentated carbazole-based XB donor scaffolds present the ideal space and angle to act as a host for anions. They found that forbidding the flexibility of the XB donor part (halotriazole in that particular case) by an intramolecular hydrogen bond increases the halogen-bonding interaction between the host molecule and the different halogen anions.

Herein, a thorough computational study has been performed in order to rationalise the experimental outcome, and to enable the prediction and better design of the XB donor motif increasing the halogen-bonding interaction involved, and therefore, enhancing the anion transporter ability. Two scaffolds have been proposed, **A** and **B** (Figure 2). For host molecule **A**, we different possibilities have been studied, named **A_R_1__R_2_:X**, where **R_1_** can be H or OH. These groups will account for the flexibility/rigidity of the scaffold respectively due to the absence or presence of an intramolecular hydrogen bond; **R_2_** substituents were selected in terms of the electrodonating (mes), or electrowithdrawing (per) capacity; and finally, **X** stands for the halogen anion to be trapped, i.e., Cl^-^, Br^-^ and I^-^. For host molecule **B**, no R1 groups but H were studied due to methyl groups on the pyridinium motif which prevents intramolecular hydrogen-bonding interactions. Therefore the nomenclature followed was: **B_H_R_2_:X**.

#### 2.1.1. MEPs Monomers

**Figure 3:**
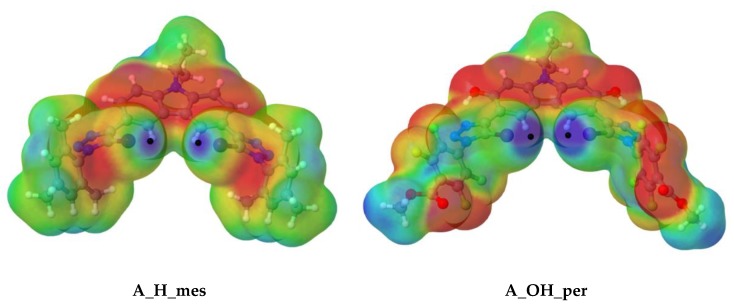
Molecular electrostatic potential on the 0.001 a.u electron density isosurface for **A_H_mes** and **A_OH_per** scaffolds at the m062x/aug–cc–pVDZ computational level. Colour scheme range: Red (−0.015 a.u.) to Blue (+0.05 a.u.).

We began by calculating the molecular electrostatic potential (MEP) surfaces for the four neutral different monomers (**A**) to analyse the areas susceptible of halogen-bonding. The maxima of the MEP on the 0.001 a.u. electron density isosurface are plotted in Figure 3 (and Figure S1) and summarised in Table 1.

Two MEP maxima values (black dots) were found for the halogen atoms, (V_max,I_), corresponding to a σ-hole on the C-I bond axis.

As observed, the depth of V_max,I_ increases with the electro-withdrawing substituent (from **mes** to **per**), as well as with the rigidity of structure, in which an intermolecular hydrogen bond is presented (H to OH). Therefore, the deepest σ-hole of the four (0.705 au) was found for **A_OH_per**, so, in principle, one would expect that the interaction between **A_OH_per** and the corresponding anion would be the strongest.

#### 2.1.2. Structural Analysis

We began by performing a conformational analysis for two monomers, **A_H_mes** and **A_OH_per**, studying the corresponding stability and barriers between the two different conformations, *cis* and *trans* (Figure S2 and Table S2). In both cases, the *cis* conformer is more stable than the *trans* one. When the scaffold does not exhibit an intramolecular hydrogen bond, **A_H_mes**, the difference between both conformers is only 1 kJ/mol, while when the intramolecular HB takes place, **A_OH_per**, the difference between both conformations increases 12 kJ/mol, favouring isomer *cis*. This is in agreement with what was found in crystal structures in which the scaffold interacts with the corresponding anion using the *cis* conformer.

**Figure 4:**
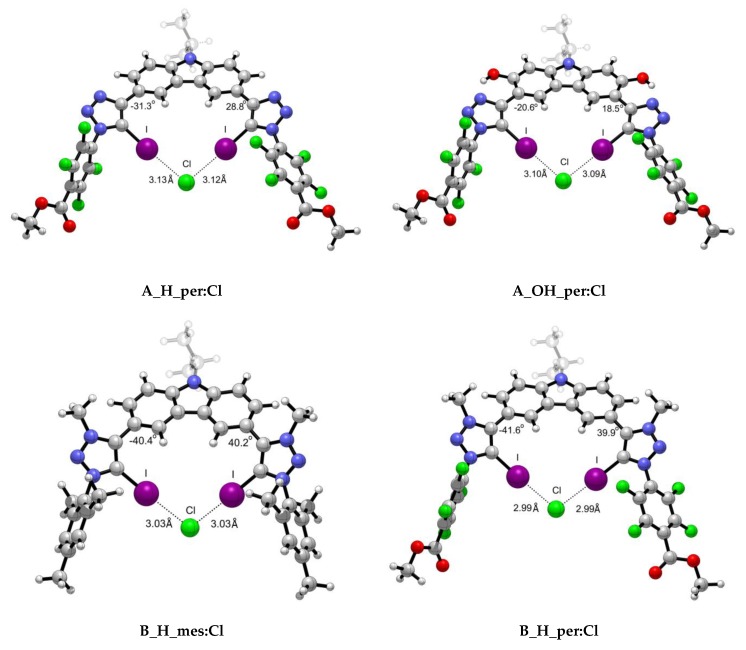
Representative optimised structures for the interaction between scaffolds **A** and **B** with Cl^−^ anion.

A total of 18 complexes were found due to the interaction between scaffolds **A** and **B** with three different halides, i.e., Cl^−^, Br^−^, and I^−^. Two of the optimised geometries obtained for each **A** and **B** scaffolds are depicted in Figure 4 for illustrative purposes, while the rest have been included in the ESI (Table S1).

As observed from the structures in Figure 4 and the collected data in Table 2, the intermolecular distance between the X^−^ anion and each of the iodine atoms is almost identical for both X-⋯I interactions, despite the slight variations on the MEP maxima values on the σ-hole. The highest degree of asymmetry regarding the dihedral angle formed between the scaffold and the halide atom (Figure 4) is associated to the iodine anion and the cationic XB motif. The rigid structures in which an intramolecular hydrogen bond is involved exhibit the highest planarity. The X⋯I intermolecular distances were found to be shorter in the *per* than in the *mes* complexes in alignment with the MEP maxima found for the σ-holes. Also, shorter distances were found for complexes with intramolecular hydrogen bonds (H vs. OH) concomitantly with more planarity shown by the dihedral angles between both rings (as per Figure 4). Finally, the shortest intermolecular X⋯I distances were found for the cationic XB (**B**) complexes, in agreement with the electrostatic nature of the interaction. As expected, the distances correspond to halide radii: Cl < Br < I. Tepper et al.’s [12] experimental data indicate that **A_H_mes:Cl** complex exhibits a I⋯Cl- intermolecular distance of 3.11 Å, which is in fair agreement with our computational value 3.17 Å. In the same work, the authors studied the influence of the OH group in the arrangement and I⋯Cl- distance. Their crystallographic data shows a trimer complex (**A_OH_mes)_2_:Cl** in which four simultaneous XB are taking place. The intermolecular distances reported vary from 3.09 to 3.23 Å due to the orientation of the scaffolds. Our theoretical distance, i.e., 3.14 Å, is in good agreement with one of the distances reported (3.13 Å) and with the average of all of them (3.135 Å).

The geometric patterns of Table 2 are reflected in the trend of the electron density values at the bond critical points (BCPs) corresponding to the halogen-bonding interactions (Table 2). Molecular graphs for all the complexes are summarised in Table S1, and the values of the Laplacian (∇^2^ρ) in Table 2 indicate that all the interaction considered are within the closed shell range. Therefore, from the geometric perspective, complex **B_H_per** scaffold manifest the strongest electron density value, as well as the shortest X-I distance, revealing halotriazolium as a highly potent anion-binding XB motif, and therefore, allowing strong coordination to occur with the halide ions.

#### 2.1.3. Energetic Analysis

The binding energies (E_b_) reported in Table 3 obey a systematic trend. These complexes are strongest for Cl and weakest for I, presenting almost the same gap between Cl and Br atoms and Br and I atoms. This pattern mirrors the noncovalent bond lengths in Table 2.

It is commonly found that binding energies are substantially diminished when vibrational and entropic factors are included in the formulation. The Gibbs free energies of the association reactions reported in Table 3 do indeed indicate a weakening by less negative quantities. But this weakening is relatively modest, and the binding free energies are still quite substantial. In fact, all quantities in Table 3 are negative, indicating spontaneous association processes. The evolution of the free energy values in Table 3 closely mimics those of E_b_, except in one case, **B_H_mes**, in which the trend is the opposite, obtaining the lowest ΔG_b_ when the iodide ion is interacting with the host.

The anion-scaffold binding has important implications for the selectivity of these receptors. The selectivity for Cl^-^ over the other two anions can be evaluated by the equilibrium constant, k = exp $\left\{\frac{\left\{\Delta G(X^{-})-\Delta G(Cl^{-})\right\}}{RT}\right\}$, and the resulting quantities are reported in the last column of Table 3. In all cases, the values > 1 indicate that the binding of the/a chloride ion to the receptor is favoured over the other two, i.e., bromide and iodine, ions. The only exception is the **B_H_mes** complex, in which the opposite was found. Additionally, the equilibrium constant for the halogen-bonding interaction upon complexation for all complexes **A** and **B** was calculated and gathered in Table S3, ranging from a minimum of 84.7 for **A_H_mes:I** up to 4.73 × 10^16^ for **B_H_per:Cl.** It is observed that the largest equilibrium constant corresponds to the complexes **B**, in which ks range on the order of 10^13^–10^16^ according to the binding energy trend.

Finally, an NBO analysis of the wave function in order to characterise the intramolecular halogen–bonding was performed. To describe these bonds, a charge transfer from the lone pair(s) of the halide to σ*(X-C) antibonding orbital has to be localised. The energetic component of these charge transfers, E(2), is displayed in Table 4. All the E(2) are quite large and, as expected, diminish with the size of the halide: Cl > Br > I. A nice and clear correlation is found between the binding energies (E_b_) and the magnitude of E(2). The only exception to this trend is the complex **A_H_mes**, presenting a higher values of E(2) than expected. However, it is well-known that the E(2) charge-transfer energies are not the only factor in the binding of the halides.

### 2.2. Bis-Iodotriazole Pyridinium-Based XB Donor Motif

In the second part of this work, bis-iodotriazole-pyridinium motif based receptors were studied, since those molecules are capable of binding anions by two bifurcated halogen bonds. Beer et al. showed, from an experimental perspective, the evidence of the halogen bond interactions involving those pyridinium based scaffolds and halide atoms [11].

**Figure 5:**
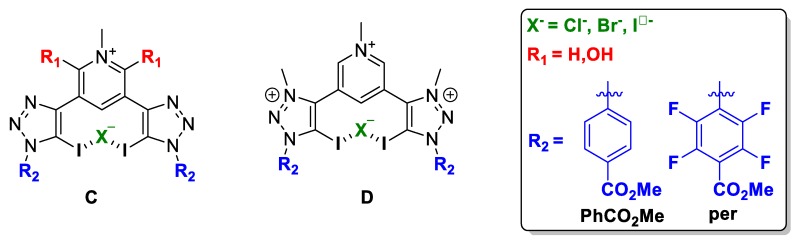
Schematic representation of the compound subject to study: (**C**) neutral XB donor motif and (**D**) cationic XB donor motif.

Two different XB donor motif were studied, i.e., halotriazole (neutral) and halotriazolium (cationic), and therefore, two families of compounds were optimised, **C** and **D** (Figure 5).

#### 2.2.1. Structural Analysis

**Figure 6:**
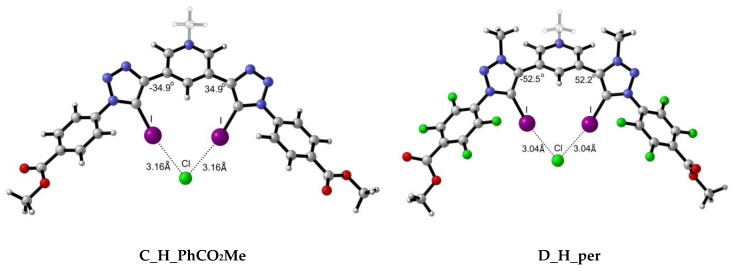
Representative optimised structures for the interaction between scaffolds **C** and **D** with Cl^−^ anion.

A total of 18 complexes were found due to the interaction between scaffolds **C** and **D** with three different halides, Cl^−^, Br^−^ and. Two of the optimised complexes obtained, one for each **C** and **D,** are depicted in Figure 6 for illustrative purposes, while the rest are included in the ESI (Table S1).

Intermolecular X⋯I distances (Table 5) for **C** complexes are in the same range as those for **A** complexes, with slightly shorter distances for **A_H_per** complexes with respect to the **C_H_per** ones. Despite the fact that **C** complexes present a positive formal charge, the larger scaffold presented in complexes **A** allow for a better orientation of the XB donor motifs in **C** complexes. This can be noticed by the I⋯I intramolecular distance, for example in **A_OH_per:Cl** (4.69 Å) and **C_OH_per:Cl** (3.85 Å) complexes. The larger scaffold in the former allows longer I⋯I separation, resulting in less electronic repulsion between the two I atoms and, therefore, better accommodation of the halide. Also, as observed by the dihedral angles, intramolecular hydrogen bond-driven rigid systems allow for better orientations and, therefore, shorter X⋯I distances. Bunchuay et al. [16] provided structural information about the mono cationic acyclic XB bis-iodotriazole pyridinium (**C_H_per**) receptor with Cl^−^ and I^−^. The intramolecular distances reported for **C_H_per:Cl** are 3.007 and 3.142 Å, since neither donor motif is symmetric. Our theoretical I⋯Cl^−^ of 3.14 is in good agreement with the experimental ones. In case of **C_H_per:I**, slightly larger discrepancies are found in the I⋯I^−^ distances between Bunchuay’s work (3.401–3.357 Å) and the computed distances in the present work (3.50 Å), although they are still within the same range.

Triply charged complexes, **D**, present shorter distances than **C** and **A** complexes, but slightly longer than **B** ones, where, intuitively, one would expect the opposite. Again, the larger the scaffold, the better the orientation; the lower electronic repulsion explains why **B** complexes exhibit shorter X⋯I than **D** complexes. Electron density and Laplacian values (Table 5) are consistent with the intermolecular distances pattern found as well.

#### 2.2.2. Energetic Analysis 

The binding and free energies for **C** and **D** complexes are gathered in Table 6. Value analysis indicated that the general trend on both ΔE_b_ and ΔG_b_ is as follows: **A**< **C** < **D** < **B**. In spite of binding energies for **C** complexes being stronger than **A** complexes, both families have very close binding energy values. Again, this can be explained in terms of the size of the scaffold which accommodates the halide better in **A** than in **C**. The positive charge in **C** complexes will increase the interaction with the halide, but somehow, it suffers for some penalty due to the electronic repulsion between I atoms in the XB donor motif. This explains why the interaction energy in **C** complexes is stronger than **A**, but not as strong as one should expect. When moving to more charged systems, the binding energies are much larger (see **C** and **D** complexes in Table 6). As with the **A** and **C** complexes, the **D** < **B** difference can be explained in the same scaffold-size/electronic repulsion terms.

Regarding the equilibrium constants with respect to the chloride anion, bromide is more competitive in **C** and **D** complexes than in **B** or **A** ones. Chloride is in almost all cases favoured over iodine. Finally, and similar to what was observed for the **A** and **B** complexes, the NBO E(2) values trend (Table 7) is in agreement with the binding energies found across the halide anion.

## 3. Discussion

**Figure 7:**
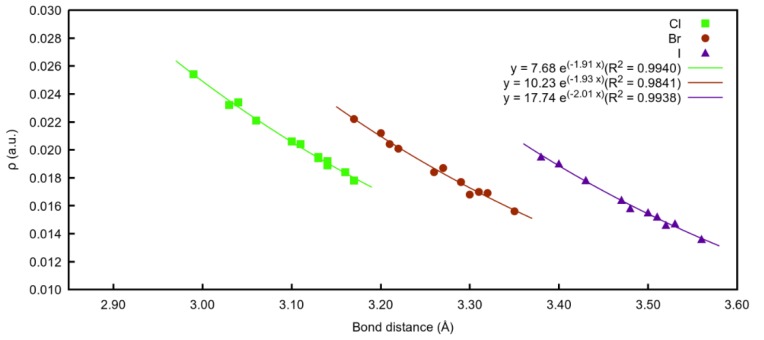
Exponential correlation density at BCPs vs. bond distances (X⋯I) regarding the three different halide atoms involved.

A total of 36 compounds were characterised, four different organic scaffolds interacting with three different halide atoms. Very good exponential relationships between the electron density values at the BCPs and the intramolecular X⋯I distance (X=Cl^−^, Br^−^ or I^−^) were found (Figure 7). As observed, when the anion involved in the interaction is chloride, all the distances are shorter, and therefore, present the highest density values for all the families.

When the same relationship between density and X⋯I distances is made regarding the 12 different families (Figure S4), the same trend was found for the three halide atoms. Additionally, **B** and **D** complexes with electro withdrawing substituent, *per*, appear to be the best options in order to get the strongest halogen bonding interaction, following the scaffolds that establish an intramolecular hydrogen bond (**A_OH_per** and **C_OH_per**). 

In terms of binding energies, chloride-based complexes are the strongest ones, and the binding energies evolve with the electron donor capacity of the anion as Cl^−^ > Br^−^ > I^−^.

It is clear that the scaffold plays an important role in establishing halogen bond interactions. Larger scaffolds accommodate the halide better than smaller scaffolds. This reduces the electronic repulsion between iodine atoms (XB donors) and increases the overlap between the halide electron lone pairs and the I-C σ* antibonding orbital, as evidenced by the larger E(2) values for **A_per** complexes with respect to the **C_per** ones.

Also, the presence of OH groups and the possibility of forming intramolecular hydrogen bonds increases the planarity of the molecule, particularly between the scaffold and XB donor motifs, increasing the rigidity and enhancing the halogen bond.

## 4. Computational Methods

Structures of the complexes were optimized at the m062x/aug–cc–pVDZ [17,18] computational level. Harmonic vibrational frequencies were computed at the same level used for the geometry optimizations in order to confirm that the stationary points are local minima. For the heavy atom I, the aug-cc-pVDZ-PP pseudopotential [19,20] was used to incorporate relativistic effects. Calculations were performed using the Gaussian16 software [21]. Single point energies for lowest energy small basis set calculations were computed using m062x/aug–cc–pVTZ. Binding energies (E_b_) were calculated as a difference of the energy of the optimised complex minus the energy of each monomer in their optimised geometry. The free energies reported in the document were obtained by adding the free energy correction from the small basis set calculations to the potential energy obtained from the high-level single-point energy calculations. 

Solvent effects (THF for **A** and **B** complexes and DMSO for **C** and **D** complexes) were included in the optimization by means of a continuum method, the Solvation Model based on Density (SMD) approach [22], and the refined SMD18 [23] version for Br and I atoms, implemented in Gaussian16.

The molecular electrostatic potential (MEP) of the isolated monomers were calculated on the electron density isosurface of 0.001 au. This isosurface has been shown to resemble the van der Waals surface [24]. These calculations were carried out with the Gaussian-16 software and the numerical results analysed using the Multiwfn [25] and plotted using Jmol [26].

The Atoms in Molecules (AIM) methodology [27,28] was used to analyse the electron density of the systems with the AIMAll program [29]. The Natural Bond Orbital (NBO) method [30] was employed to evaluate atomic charges using the NBO-3 program, and to analyse charge-transfer interactions between the occupied and unoccupied orbitals.

## Figures and Tables

**Table 1:** Maxima (V_max,I_) values of the molecular electrostatic potential (in a.u.) on the 0.001 a.u. electron density isosurface for all the monomers (**A**) calculated at m062x/aug–cc–pVDZ level. Due to the lack of symmetry slight variations are found the two different iodine atoms (1 and 2).

Monomers	1	2
**A_H_mes**	0.0512	0.0512
**A_H_per**	0.0614	0.0618
**A_OH_mes**	0.0605	0.0605
**A_OH_per**	0.0705	0.0711

**Table 2:** Electron density at the BCP (ρ), Laplacian (∇^2^ρ), and interaction distance (Å) for all the complexes studied at m062x/aug–cc–pVDZ computational level. Dihedral angles (Figure 4).

	ρ			∇^2^ρ			d(I⋯X)			Dihedral		
Complexes	Cl	Br	I	Cl	Br	I	Cl	Br	I	Cl	Br	I
**A_H_mes**	0.0178	0.0156	0.0136	0.0521	0.0409	0.0329	3.17	3.35	3.56	−29.36	−31.10	−32.17
**A_H_per**	0.0195	0.0170	0.0152	0.0564	0.0441	0.0358	3.13	3.31	3.51	−31.26	−32.40	−32.81
**A_OH_mes**	0.0189	0.0168	0.0146	0.0548	0.0437	0.0347	3.14	3.30	3.52	−14.07	−14.92	−16.27
**A_OH_per**	0.0206	0.0184	0.0158	0.0589	0.0470	0.0369	3.10	3.26	3.48	−20.59	−20.02	−24.22
**B_H_mes**	0.0232	0.0204	0.0178	0.0643	0.0501	0.0394	3.03	3.21	3.43	−40.36	−43.14	−42.88
**B_H_per**	0.0254	0.0222	0.0195	0.0683	0.0529	0.0414	2.99	3.17	3.38	−41.56	−44.34	43.68

**Table 3:** Binding energies (E_b_) and binding free energy (ΔG_b,_ at 298K) (kJ/mol) upon complexation at m062x/aug–cc–pVTZ computational level. Equilibrium constants for the halogen-bonding interaction.

	E_b_ (kJ/mol)			ΔG_b_ (kJ/mol)			K	
Complexes	Cl	Br	I	Cl	Br	I	Cl^−^/Br-^−^	Cl^−^/I-^−^
**A_H_mes**	−49.28	−46.90	−44.92	−15.91	−13.30	−11.00	2.86	7.24
**A_H_per**	−58.73	−56.40	−52.83	−24.82	−24.07	−19.54	1.36	8.45
**A_OH_mes**	−57.06	−53.84	−50.78	−19.47	−19.37	−17.57	1.04	2.16
**A_OH_per**	−66.39	−62.59	−58.69	−35.14	−30.53	−29.95	6.42	8.13
**B_H_mes**	−113.91	−109.10	−104.30	−77.92	−79.48	−81.98	0.53	0.19
**B_H_per**	−125.23	−120.85	−114.57	−95.14	−92.42	−86.63	2.99	3.09

**Table 4:** NBO values of charge transfer energy E(2), in kJ/mol, from lone pairs of halide to σ*(X-C) antibonding orbital.

	E(2) (kJ/mol)		
Complexes	Cl	Br	I
**A_H_mes**	122.67	125.56	116.94
**A_H_per**	109.04	97.61	90.96
**A_OH_mes**	79.04	73.55	70.37
**A_OH_per**	119.24	111.34	98.24
**B_H_mes**	167.03	141.63	72.01
**B_H_per**	170.37	154.56	144.81

**Table 5:** Electron density at the BCP (ρ), Laplacian (∇^2^ρ), and interaction distance (Å) for all the complexes studied at m062x/aug–cc–pVDZ computational level. Dihedral angles (Figure 6).

	ρ			∇^2^ρ			d(X⋯I)			Dihedral		
Complexes	Cl	Br	I	Cl	Br	I	Cl	Br	I	Cl	Br	I
**C_H_PhCO_2_Me**	0.0184	0.0169	0.0147	0.0537	0.0438	0.0351	3.16	3.32	3.53	−34.88	−36.34	−37.14
**C_H_per**	0.0192	0.0177	0.0155	0.0554	0.0453	0.0363	3.14	3.29	3.50	−34.45	−35.81	−37.24
**C_OH_PhCO_2_Me**	0.0194	0.0177	0.0155	0.0560	0.0454	0.0364	3.13	3.29	3.50	−8.21	−8.76	−7.30
**C_OH_per**	0.0204	0.0187	0.0164	0.0582	0.0471	0.0377	3.11	3.27	3.47	−1.60	−1.49	−1.17
**D_H_PhCO_2_Me**	0.0221	0.0201	0.0178	0.0618	0.0497	0.0396	3.06	3.22	3.43	−52.24	−52.25	−52.57
**D_H_per**	0.0234	0.0212	0.0190	0.0642	0.0512	0.0408	3.04	3.20	3.40	−52.46	−52.81	−53.01

**Table 6:** Binding energies (E_b_) and binding free energy (ΔG_b,_ at 298K) (kJ/mol) upon complexation at m062x/aug–cc–pVTZ computational level. Equilibrium constants for the halogen-bonding interaction.

	ΔE_b_ (kJ/mol)			ΔG_b_ (kJ/mol)			K	
Complexes	Cl	Br	I	Cl	Br	I	Cl^−^/Br^−^	Cl^−^/I^−^
**C_H_PhCO_2_Me**	−59.42	−58.21	−56.10	−28.80	−27.51	−28.16	1.68	1.29
**C_H_per**	−64.34	−62.79	−60.67	−33.75	−34.47	−30.58	0.75	3.60
**C_OH_PhCO_2_Me**	−62.67	−60.91	−58.18	−28.31	−29.06	−33.90	0.74	2.70
**C_OH_per**	−69.11	−67.24	−64.13	−35.48	−34.06	−33.02	1.78	2.70
**D_H_PhCO_2_Me**	−83.64	−81.56	−78.05	−49.20	−54.26	−49.65	0.13	0.83
**D_H_per**	−90.66	−88.32	−84.36	−53.14	−55.26	−51.07	0.42	2.30

**Table 7:** NBO values of charge transfer energy E(2), in kJ/mol, from lone pairs of halide to σ*(X-C) antibonding orbital.

	E(2) (kJ/mol)		
Complexes	Cl	Br	I
**C_H_PhCO_2_Me**	94.81	91.34	82.93
**C_H_per**	101.42	98.58	89.79
**C_OH_PhCO_2_Me**	103.01	99.16	90.50
**C_OH_per**	111.92	108.24	99.50
**D_H_PhCO_2_Me**	128.74	124.35	115.06
**D_H_per**	140.71	135.31	127.24

## Supplementary Materials

The following are available online, Table S1: Molecular graphs and optimized geometries for all complexes, Figure S1: Molecular electrostatic potential for selected monomers (A), Figure S2: Optimised *trans* isomer for scaffolds **A_H_mes** and **A_OH_per**, Table2: Relative energies (ΔE) and binding free energy (ΔG_,_) for *cis* and *trans* conformers, Table S3: Equilibrium constants for the halogen-bonding interaction for complexes **A** and **B**, Figure S3: Molecular electrostatic potential for selected monomers(**C**), **Table S4**. Maxima (V_max,X_) values of the molecular electrostatic potential for all the monomers (**C**), Table S5. Equilibrium constants for the halogen-bonding interaction for complexes **C** and **D**, Figure S4. Relationship density at BCPs *vs* bond distances (I-X) for all the families under study.
