# Supplementary material for: Anion Recognition by Neutral and Cationic Iodotriazole Halogen Bonding Scaffolds
Source: Molecules. 2020 Feb 12;25(4):798. doi: 10.3390/molecules25040798 (PMC7070532; doi:10.3390/molecules25040798)
Supplement: Supplementary file 1 [file molecules-25-00798-s001.pdf]

Halogen Bond Interactions in Halogen-Bonding Receptors for Anion Recognition

Iñigo Iribarren [1], Goar Sánchez-Sanz [2] and Cristina Trujillo,[1*]

[1] *School of Chemistry, Trinity Biomedical Sciences Institute, Trinity College Dublin, 152-160 Pearse St., Dublin 2, Ireland*

[2]*Irish Centre of High–End Computing, Grand Canal Quay, Dublin 2, Ireland*

Supporting Information

- **Table S1.** Molecular graphs (AIM) and optimized geometries (Cartesian Coordinates in Å) for all the complexes under study. Green dots indicate bond critical (BCP)
- **Figure S1.** Molecular electrostatic potential for selected monomers (**A)**
- **Table S2.** Equilibrium constants for the halogen-bonding interaction for complexes **A** and **B**
- **Figure S2.** Molecular electrostatic potential for selected monomers (**C)**
- **Table S3.** Maxima ($V_{max,X}$) values of the molecular electrostatic potential for all the monomers (**C**)
- **Table S4.** Equilibrium constants for the halogen-bonding interaction for complexes **C** and **D**
- **Figure S3**. Relationship density at BCPs *vs* bond distances (X···I) for all the families under study

Table S1. Molecular graphs (AIM) and optimized geometries (Cartesian Coordinates in Å) for all the complexes under study at m062x/aug-cc-pVDZ computational level. Green dots indicate bond critical (BCP).

Compound	Coordinates			
	Atom	X	Y	Z
**A_H_mes_Cl**	I	2.45165	-1.02458	-0.50736
Img. Freq. = 0	I	-2.35552	-1.14389	-0.49684
SCF Energy= -2865.84834800	Cl	0.09043	-2.85435	-1.57219
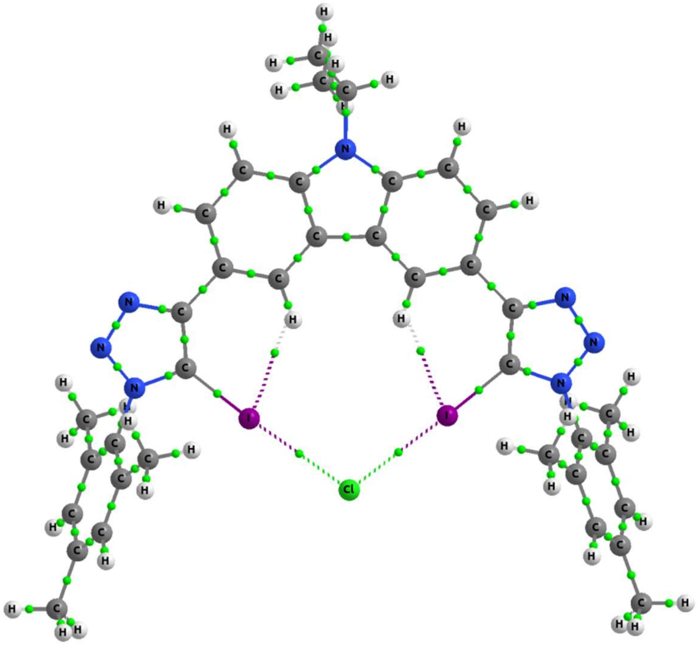	H	4.41199	4.20869	0.02718
	H	-4.52431	4.01356	0.0073
	N	-0.08123	5.42127	-0.06994
	N	5.28765	1.86795	0.9318
	N	5.99886	0.781	0.91543
	N	5.20908	-0.21527	0.49967
	N	-5.30211	1.65021	0.91714
	N	-5.97169	0.53745	0.90681
	N	-5.14395	-0.43129	0.50009
	C	-0.76072	3.29302	0.39382
	C	-1.19247	4.60574	0.0882
	C	1.05832	4.64872	0.09438
	C	0.68038	3.32104	0.39843
	C	1.63628	2.32313	0.57867
	H	1.31984	1.31475	0.8355
	C	2.98415	2.6374	0.42284
	C	3.35489	3.9725	0.13811
	C	2.4164	4.98548	-0.01967
	H	2.74567	5.99723	-0.24627
	C	4.0149	1.59268	0.52679
	C	3.95547	0.23297	0.23741
	C	5.72023	-1.54948	0.37585
	C	6.30442	-1.92731	-0.83864
	C	6.78437	-3.23233	-0.94022
	H	7.24538	-3.55537	-1.87466
	C	6.68205	-4.13639	0.12473
	C	6.08561	-3.71354	1.31468
	H	5.99849	-4.40984	2.14922
	C	5.58958	-2.41466	1.46346
	C	6.39094	-0.95241	-1.98078
	H	6.9179	-1.40101	-2.82843
	H	6.92017	-0.03978	-1.67859
	H	5.38741	-0.65614	-2.31629
	C	7.2072	-5.5396	-0.02721
	H	6.68829	-6.05877	-0.84351
	H	7.06713	-6.1166	0.8931
	H	8.27734	-5.52886	-0.27174
	C	4.92682	-1.95612	2.7335
	H	3.85974	-1.75532	2.56415

	H	5.3831	-1.029	3.10278
	H	5.01129	-2.72391	3.50869
	C	-0.074	6.84273	-0.42334
	H	0.98387	7.12151	-0.43488
	C	-0.63759	7.06918	-1.8237
	H	-0.10405	6.45182	-2.5559
	H	-0.5154	8.12325	-2.10057
	H	-1.70509	6.82519	-1.87218
	C	-0.76697	7.68748	0.64207
	H	-0.33981	7.484	1.63092
	H	-1.8445	7.49232	0.67838
	H	-0.62281	8.74995	0.41207
	C	-2.56599	4.8778	-0.03142
	H	-2.94388	5.87041	-0.25951
	C	-3.45837	3.82369	0.12264
	C	-3.03147	2.50627	0.4097
	C	-1.67175	2.2529	0.56918
	H	-1.31058	1.25945	0.8258
	C	-4.01873	1.42051	0.51687
	C	-3.90717	0.06168	0.23793
	C	-5.60854	-1.78273	0.38135
	C	-6.18374	-2.18288	-0.83029
	C	-6.62478	-3.50204	-0.92543
	H	-7.07792	-3.84263	-1.85748
	C	-6.49386	-4.39784	0.14341
	C	-5.90772	-3.95235	1.33022
	H	-5.79875	-4.6421	2.16761
	C	-5.45003	-2.63868	1.47248
	C	-6.30221	-1.2152	-1.97569
	H	-6.81204	-1.68437	-2.82267
	H	-5.309	-0.88523	-2.31045
	H	-6.86323	-0.32054	-1.67705
	C	-6.97851	-5.81638	-0.00074
	H	-6.44855	-6.32351	-0.81749
	H	-8.04964	-5.83782	-0.24024
	H	-6.81752	-6.38537	0.92113
	C	-4.79993	-2.15472	2.73963
	H	-3.73893	-1.92522	2.56865
	H	-4.86275	-2.92016	3.51917
	H	-5.28186	-1.23865	3.10387

Compound	Coordinates			
	Atom	X	Y	Z
**A_H_mes_Br**	I	2.53197	-0.90788	-0.49135
Img. Freq. = 0	I	-2.43935	-1.03231	-0.47358
SCF Energy= -4979.89134708	Br	0.0862	-2.84127	-1.72587
	H	4.40628	4.32505	0.03396
	H	-4.53523	4.12468	0.01386

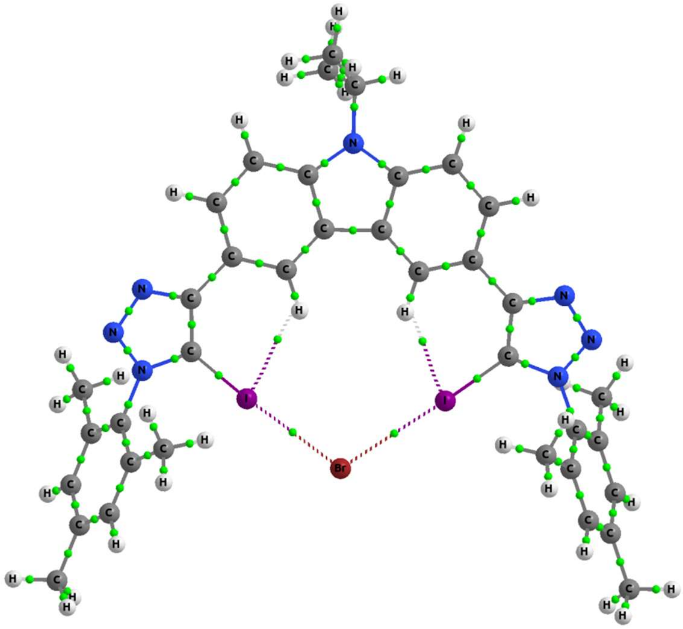

N	-0.08969	5.51436	-0.13098
N	5.27037	2.01598	1.05609
N	5.99315	0.93663	1.07908
N	5.23373	-0.07121	0.63587
N	-5.30134	1.79188	1.03687
N	-5.98005	0.68474	1.06509
N	-5.17807	-0.2954	0.63493
C	-0.76855	3.39485	0.37431
C	-1.20016	4.70248	0.04554
C	1.04995	4.74701	0.05224
C	0.67342	3.42365	0.37878
C	1.63345	2.43471	0.58868
H	1.32197	1.4291	0.86325
C	2.98151	2.75546	0.44535
C	3.34898	4.08554	0.1357
C	2.40705	5.08898	-0.05558
H	2.73288	6.09794	-0.29884
C	4.0197	1.72409	0.59712
C	3.98888	0.36268	0.31547
C	5.76011	-1.40248	0.5506
C	6.4089	-1.787	-0.62846
C	6.90015	-3.09012	-0.69344
H	7.4108	-3.41841	-1.59984
C	6.74707	-3.9857	0.37265
C	6.08687	-3.55641	1.52612
H	5.95945	-4.24633	2.36075
C	5.57663	-2.25932	1.63731
C	6.54932	-0.82135	-1.77307
H	7.1152	-1.27685	-2.59141
H	7.06439	0.09356	-1.45391
H	5.56274	-0.5275	-2.15737
C	7.28761	-5.38684	0.26086
H	6.8263	-5.91109	-0.58613
H	7.08999	-5.96141	1.17209
H	8.37158	-5.37151	0.08765
C	4.84389	-1.79453	2.86599
H	3.78497	-1.60779	2.63899
H	5.27035	-0.85851	3.24823
H	4.89595	-2.55306	3.65305
C	-0.08404	6.92975	-0.50808
H	0.97406	7.20604	-0.53739
C	-0.66295	7.13429	-1.90557
H	-0.13987	6.50262	-2.63306
H	-0.5398	8.18305	-2.20144
H	-1.73181	6.89389	-1.93845
C	-0.76268	7.79334	0.55157
H	-0.32204	7.60711	1.53792
H	-1.83956	7.59867	0.60609

	H	-0.62181	8.85162	0.30115
	C	-2.57318	4.97838	-0.06845
	H	-2.94802	5.96778	-0.31456
	C	-3.4686	3.93289	0.11995
	C	-3.04366	2.6212	0.43314
	C	-1.68319	2.36287	0.58049
	H	-1.3259	1.37284	0.85619
	C	-4.03715	1.54702	0.58732
	C	-3.94952	0.18546	0.31816
	C	-5.6536	-1.64597	0.55479
	C	-6.28392	-2.05995	-0.62436
	C	-6.73036	-3.37942	-0.68276
	H	-7.22564	-3.7308	-1.58901
	C	-6.55166	-4.2622	0.39003
	C	-5.9116	-3.80317	1.54339
	H	-5.76493	-4.48285	2.38324
	C	-5.44647	-2.48866	1.64829
	C	-6.45351	-1.10642	-1.77515
	H	-6.99299	-1.58867	-2.59599
	H	-5.47653	-0.77385	-2.15233
	H	-7.00873	-0.2121	-1.46496
	C	-7.04421	-5.68148	0.28577
	H	-6.56235	-6.19568	-0.55592
	H	-8.1274	-5.70377	0.10843
	H	-6.83103	-6.24291	1.20168
	C	-4.73814	-1.98986	2.87793
	H	-3.68784	-1.75702	2.65364
	H	-4.75953	-2.74805	3.66671
	H	-5.20649	-1.0725	3.25648

Compound	Coordinates			
	Atom	X	Y	Z
**A_H_mes_I**	I	2.59901	-0.79699	-0.42351
Img. Freq. = 0	I	-2.51254	-0.9279	-0.39937
SCF Energy= -2701.27424338	I	0.0873	-2.94901	-1.76473
	H	4.40479	4.45409	-0.00263
	H	-4.53938	4.24757	-0.01096
	N	-0.0932	5.61926	-0.22739
	N	5.26583	2.19029	1.1193
	N	5.99943	1.11997	1.18423
	N	5.26176	0.09333	0.74821
	N	-5.29886	1.96282	1.11793
	N	-5.98975	0.86507	1.18807
	N	-5.21096	-0.13455	0.76079
	C	-0.77062	3.51335	0.336
	C	-1.20252	4.81254	-0.02596
	C	1.04677	4.85901	-0.0215
	C	0.67215	3.54333	0.33864

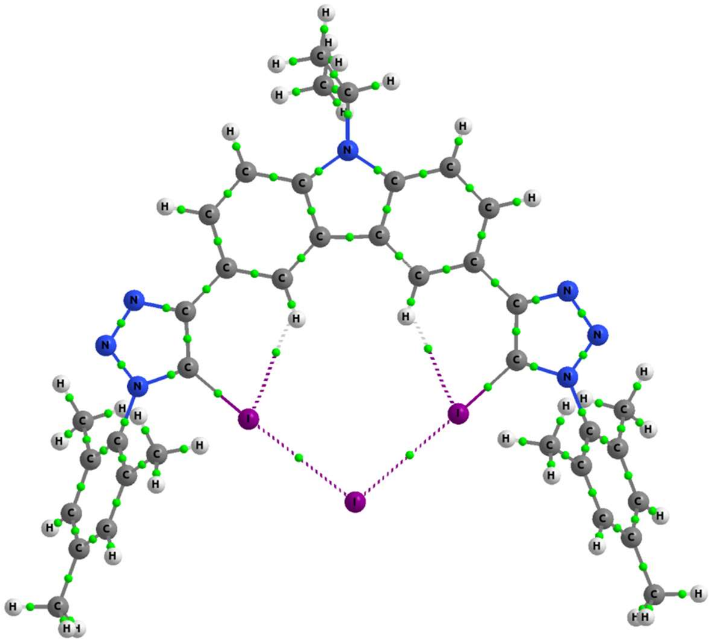

C	1.63617	2.56514	0.58149
H	1.3293	1.56536	0.88218
C	2.98379	2.89027	0.44169
C	3.34778	4.21239	0.09673
C	2.40269	5.2046	-0.13062
H	2.72504	6.2081	-0.39951
C	4.02891	1.87396	0.63935
C	4.02023	0.50668	0.39031
C	5.80253	-1.23455	0.71128
C	6.48455	-1.64402	-0.44033
C	6.98982	-2.94334	-0.45727
H	7.52648	-3.29073	-1.34121
C	6.81832	-3.81126	0.62876
C	6.1247	-3.35789	1.75296
H	5.98285	-4.02625	2.60265
C	5.5994	-2.06356	1.816
C	6.64403	-0.70845	-1.60721
H	7.23433	-1.18031	-2.39857
H	7.14209	0.2202	-1.30105
H	5.66441	-0.43572	-2.02359
C	7.37434	-5.20949	0.56888
H	6.93149	-5.76418	-0.26856
H	7.16757	-5.75778	1.49417
H	8.46066	-5.1885	0.41209
C	4.83166	-1.57356	3.01308
H	3.77512	-1.41216	2.75723
H	5.23401	-0.61913	3.37539
H	4.87812	-2.3065	3.82433
C	-0.08948	7.02559	-0.63749
H	0.96856	7.29935	-0.68464
C	-0.68162	7.19899	-2.03366
H	-0.16908	6.54707	-2.75068
H	-0.55566	8.23958	-2.35594
H	-1.75207	6.96425	-2.05066
C	-0.75562	7.91454	0.40917
H	-0.30291	7.75195	1.39424
H	-1.83166	7.72094	0.48157
H	-0.61815	8.96655	0.13182
C	-2.57508	5.09027	-0.13834
H	-2.94777	6.07362	-0.41044
C	-3.47241	4.05501	0.09001
C	-3.04888	2.75212	0.43904
C	-1.68792	2.49095	0.57892
H	-1.33337	1.50727	0.88069
C	-4.04916	1.69265	0.64262
C	-3.98542	0.32494	0.40352
C	-5.70522	-1.48035	0.71954
C	-6.36455	-1.91081	-0.43768

	C	-6.82921	-3.22513	-0.4584
	H	-7.34758	-3.58909	-1.34658
	C	-6.64005	-4.08711	0.62942
	C	-5.97019	-3.61243	1.75924
	H	-5.81488	-4.27598	2.61035
	C	-5.48602	-2.30237	1.8263
	C	-6.54426	-0.98039	-1.60576
	H	-7.11167	-1.471	-2.40244
	H	-5.57066	-0.67499	-2.01342
	H	-7.07549	-0.0689	-1.30374
	C	-7.15291	-5.50155	0.56575
	H	-6.69015	-6.04161	-0.27047
	H	-8.23875	-5.51323	0.40453
	H	-6.93356	-6.04434	1.49138
	C	-4.74532	-1.78739	3.0298
	H	-3.69623	-1.57684	2.77927
	H	-4.76244	-2.52707	3.83605
	H	-5.19134	-0.85426	3.39655

	Atom	X	Y	Z
**A_H_per_Cl**	I	2.39541	-0.197	-0.49039
Img. Freq. = 0	I	-2.36082	-0.21149	-0.64621
SCF Energy= -3879.67374149	Cl	0.06294	-1.89738	-1.67764
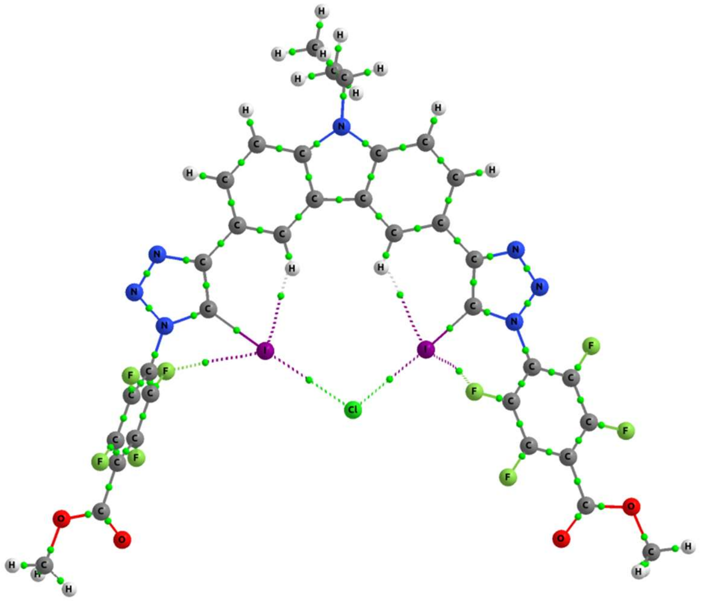	H	4.44153	4.98338	0.14322
	H	-4.49313	4.92903	-0.03976
	N	-0.02886	6.26951	-0.00672
	N	5.27317	2.62897	1.01711
	N	5.97476	1.54637	0.99977
	N	5.16513	0.55484	0.58047
	N	-5.3083	2.54363	0.86219
	N	-5.9898	1.44831	0.82557
	N	-5.16794	0.4841	0.36853
	C	-0.74915	4.14793	0.4212
	C	-1.15525	5.4702	0.12158
	C	1.09524	5.47758	0.16851
	C	0.69136	4.15324	0.45359
	C	1.62715	3.13815	0.63927
	H	1.28991	2.13377	0.88488
	C	2.98162	3.43256	0.50515
	C	3.37944	4.76356	0.23815
	C	2.4602	5.79313	0.07733
	H	2.80937	6.80117	-0.13529
	C	3.99268	2.36988	0.60989
	C	3.90928	1.01578	0.31524
	C	5.67101	-0.75691	0.4594
	C	6.67557	-1.04136	-0.4595
	C	7.18837	-2.32663	-0.56415
	C	6.70272	-3.36542	0.22628

	C	5.68744	-3.07159	1.13462
	C	5.18598	-1.78516	1.2623
	C	0.00705	7.6964	-0.33756
	H	1.06909	7.95845	-0.32357
	C	-0.52542	7.95361	-1.74467
	H	0.01018	7.33702	-2.47599
	H	-0.3777	9.00908	-2.00301
	H	-1.59636	7.73159	-1.81704
	C	-0.69377	8.53474	0.72768
	H	-0.29153	8.30705	1.72168
	H	-1.77508	8.35821	0.73763
	H	-0.52626	9.59813	0.51897
	C	-2.52184	5.76482	-0.02272
	H	-2.88029	6.76603	-0.24407
	C	-3.43277	4.72207	0.09661
	C	-3.02882	3.39556	0.37256
	C	-1.67783	3.11936	0.56234
	H	-1.33765	2.11821	0.81753
	C	-4.02763	2.31798	0.43754
	C	-3.92329	0.97427	0.10495
	C	-5.64484	-0.83882	0.24181
	C	-5.80035	-1.42623	-1.0098
	C	-5.97661	-1.57432	1.37454
	C	-6.26749	-2.7275	-1.1206
	C	-6.46474	-2.86757	1.25391
	C	-6.6158	-3.46862	0.00672
	F	-5.51581	-0.7323	-2.1094
	F	-6.41944	-3.2374	-2.34246
	F	-6.7433	-3.53555	2.37469
	F	-5.81345	-1.04533	2.58515
	F	4.24467	-1.53305	2.16929
	F	7.14043	-0.08431	-1.25977
	F	8.13342	-2.54587	-1.47999
	F	5.20393	-4.01508	1.94203
	C	-7.10627	-4.87971	-0.14826
	O	-6.60426	-5.67335	-0.90378
	O	-8.1504	-5.12211	0.63643
	C	7.21079	-4.77304	0.10412
	O	6.48758	-5.73739	0.10726
	O	8.53515	-4.80839	0.0049
	C	-8.66318	-6.46578	0.57176
	H	-7.88322	-7.17665	0.86323
	H	-9.49413	-6.49299	1.27874
	H	-9.00986	-6.68512	-0.44319
	C	9.10057	-6.12384	-0.14395
	H	8.70253	-6.60075	-1.04541
	H	10.1771	-5.96805	-0.23272
	H	8.86789	-6.73232	0.73594

	Atom	X	Y	Z
**A_H_per_Br**	I	2.49525	-0.12494	-0.59267
Img. Freq. = 0	I	-2.44509	-0.15252	-0.55396
SCF Energy= -5993.71675860	Br	0.02602	-1.91232	-1.86892
	H	4.47147	5.04696	0.04554
	H	-4.47024	4.99223	0.15833
	N	-0.00507	6.31449	-0.00497
	N	5.31888	2.70991	0.98311
	N	6.03108	1.63409	0.9753
	N	5.24017	0.63627	0.5354
	N	-5.25036	2.6311	1.1456
	N	-5.94875	1.54562	1.14859
	N	-5.17072	0.5709	0.64076
	C	-0.71132	4.19309	0.45012
	C	-1.12614	5.51633	0.16477
	C	1.12395	5.52364	0.13655
	C	0.73047	4.19806	0.43327
	C	1.67609	3.18803	0.60071
	H	1.35171	2.18181	0.85744
	C	3.0263	3.49133	0.44201
	C	3.41229	4.82229	0.16022
	C	2.4846	5.84564	0.01249
	H	2.82361	6.85473	-0.2108
	C	4.04895	2.44022	0.55015
	C	3.98606	1.08771	0.24771
	C	5.7575	-0.67269	0.43194
	C	6.79501	-0.95295	-0.45163
	C	7.31153	-2.23746	-0.54137
	C	6.81605	-3.27474	0.24528
	C	5.77681	-2.9831	1.12615
	C	5.25409	-1.70192	1.22207
	C	0.01917	7.742	-0.33505
	H	1.08092	8.00407	-0.3602
	C	-0.56387	8.00018	-1.72168
	H	-0.05138	7.38793	-2.47292
	H	-0.43056	9.05698	-1.98237
	H	-1.6355	7.77258	-1.75679
	C	-0.64104	8.57976	0.75638
	H	-0.20295	8.34971	1.73456
	H	-1.72159	8.40574	0.80591
	H	-0.4788	9.6431	0.54333
	C	-2.49597	5.81721	0.07718
	H	-2.85868	6.81888	-0.13479
	C	-3.40563	4.78075	0.24589
	C	-2.99594	3.45439	0.51294
	C	-1.63871	3.16966	0.63683
	H	-1.29374	2.16701	0.88048

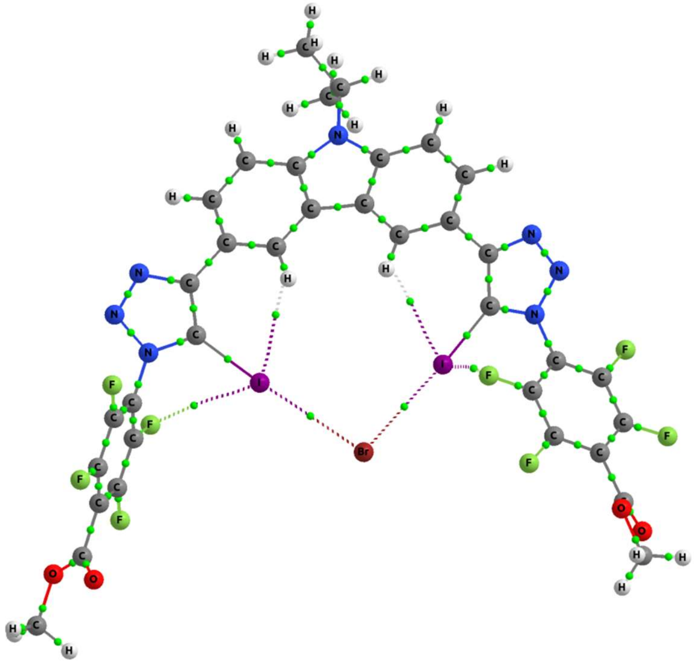

	C	-4.00174	2.38893	0.6415
	C	-3.93846	1.0451	0.3023
	C	-5.67624	-0.74268	0.5254
	C	-5.92411	-1.30331	-0.72345
	C	-5.94321	-1.49529	1.66422
	C	-6.42741	-2.59186	-0.8281
	C	-6.44259	-2.7846	1.55076
	C	-6.69594	-3.35531	0.30565
	F	-5.68268	-0.59955	-1.82726
	F	-6.62713	-3.08618	-2.05053
	F	-6.69776	-3.45881	2.67199
	F	-5.71606	-0.98288	2.87137
	F	4.26763	-1.45892	2.08276
	F	7.29646	0.00929	-1.22257
	F	8.31904	-2.44833	-1.38856
	F	5.24017	-3.93301	1.89292
	C	-7.21219	-4.76326	0.22153
	O	-6.7841	-5.66357	0.89924
	O	-8.1876	-4.87254	-0.67303
	C	7.38535	-4.65717	0.10321
	O	7.64422	-5.15753	-0.96262
	O	7.56907	-5.23276	1.28585
	C	8.08107	-6.5774	1.23909
	H	9.06798	-6.58543	0.76541
	H	8.14935	-6.89742	2.28025
	H	7.39167	-7.21956	0.68143
	C	-8.71774	-6.20058	-0.84035
	H	-9.48257	-6.11142	-1.61373
	H	-7.92217	-6.88167	-1.15931
	H	-9.15602	-6.54952	0.10018

	Atom	X	Y	Z
**A_H_per_I**	I	2.53808	-0.09033	-0.45775
Img. Freq. = 0	I	-2.5117	-0.10928	-0.56574
SCF Energy= -3715.09905157	I	0.0492	-2.12414	-1.86511
	H	4.45024	5.11264	0.16448
	H	-4.49328	5.06445	0.04841
	N	-0.02306	6.36913	-0.04076
	N	5.27771	2.80776	1.15061
	N	6.00323	1.7414	1.17576
	N	5.23715	0.72751	0.72961
	N	-5.30081	2.73309	1.06055
	N	-6.01184	1.6561	1.06799
	N	-5.23983	0.66606	0.58137
	C	-0.74264	4.25249	0.41788
	C	-1.1482	5.57403	0.11053
	C	1.10094	5.58016	0.14058
	C	0.69978	4.25683	0.43896

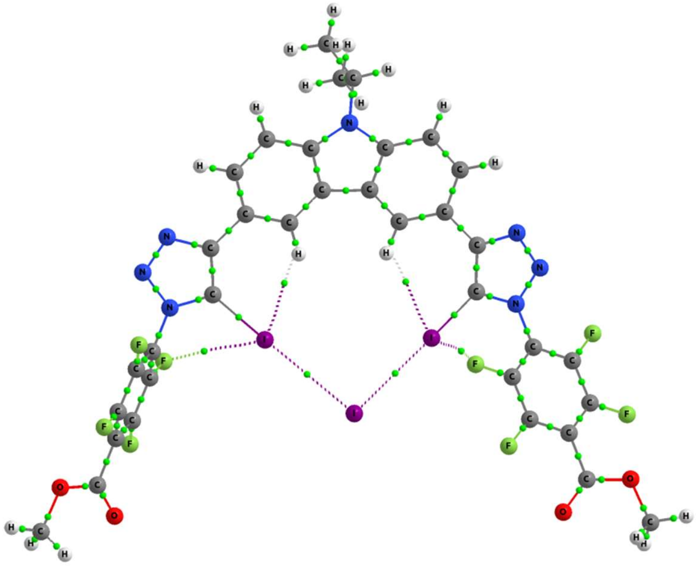

C	1.64263	3.25109	0.64738
H	1.31346	2.24647	0.90472
C	2.99656	3.55782	0.53103
C	3.38837	4.88644	0.24604
C	2.46377	5.90493	0.0561
H	2.80695	6.91285	-0.16618
C	4.02298	2.51664	0.68731
C	3.98547	1.15972	0.40338
C	5.7794	-0.57261	0.6432
C	6.84129	-0.83507	-0.21637
C	7.39378	-2.10639	-0.28186
C	6.89174	-3.15316	0.48695
C	5.81865	-2.88261	1.33352
C	5.27682	-1.60941	1.4242
C	0.0104	7.79274	-0.38657
H	1.07336	8.05025	-0.4019
C	-0.55358	8.03812	-1.78341
H	-0.03739	7.41203	-2.52054
H	-0.40697	9.09032	-2.05529
H	-1.62677	7.82055	-1.82965
C	-0.66058	8.64484	0.68703
H	-0.23433	8.42573	1.67294
H	-1.74195	8.47279	0.72596
H	-0.49412	9.70534	0.46328
C	-2.51436	5.88001	-0.00727
H	-2.86787	6.88133	-0.23558
C	-3.43122	4.84978	0.15635
C	-3.03184	3.52507	0.44572
C	-1.67838	3.23454	0.59653
H	-1.34364	2.23264	0.8577
C	-4.04894	2.47062	0.57426
C	-3.99836	1.12275	0.25229
C	-5.76253	-0.64134	0.47286
C	-5.99846	-1.21337	-0.77333
C	-6.06245	-1.37442	1.61572
C	-6.51292	-2.49768	-0.86959
C	-6.59772	-2.65043	1.51089
C	-6.83025	-3.23633	0.26873
F	-5.74463	-0.52014	-1.88086
F	-6.74095	-2.99104	-2.08624
F	-6.84329	-3.31641	2.64026
F	-5.82437	-0.85979	2.82003
F	4.27992	-1.37807	2.27565
F	7.32559	0.12943	-0.99566
F	8.39617	-2.30596	-1.13946
F	5.31562	-3.83518	2.11794
C	7.4459	-4.54644	0.405
O	6.75126	-5.53013	0.3542

	O	8.77429	-4.54409	0.40655
	C	-7.37721	-4.62793	0.12678
	O	-6.95297	-5.42519	-0.67142
	O	-8.37578	-4.84875	0.97462
	C	-8.93716	-6.17343	0.92403
	H	-8.16271	-6.9151	1.14485
	H	-9.71493	-6.1878	1.68948
	H	-9.3642	-6.36073	-0.06649
	C	9.38665	-5.84279	0.30156
	H	9.0711	-6.32966	-0.62682
	H	10.4618	-5.65623	0.29366
	H	9.10628	-6.45878	1.16211

Compound	Coordinates			
	Atom	X	Y	Z
**A_OH_mes_Cl**	I	2.42097	-1.28732	-0.26636
Img. Freq. = 0	I	-2.31617	-1.3979	-0.27687
SCF Energy= -3016.32974369	Cl	0.09754	-3.32022	-0.84083
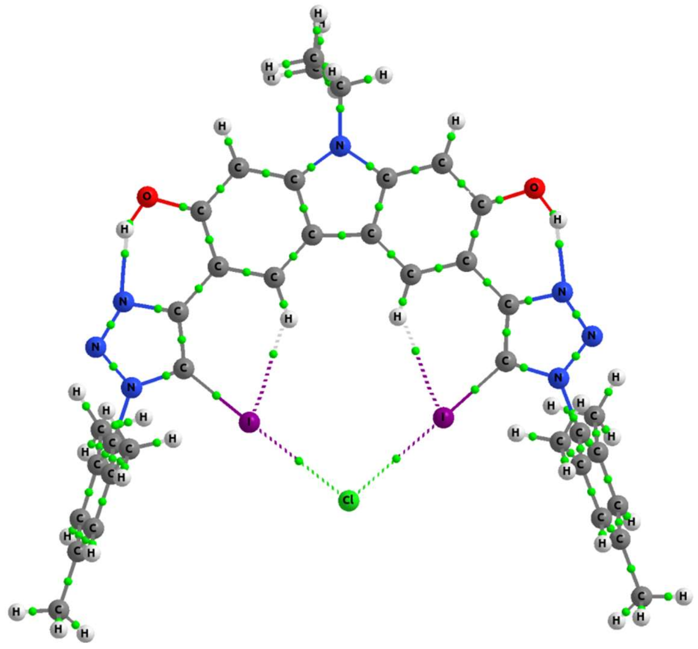	O	4.65439	4.31019	0.06577
	O	-4.79375	4.10226	-0.00521
	H	-5.34016	3.30634	0.16412
	N	-0.09043	5.33938	-0.0683
	N	5.33669	1.78706	0.4795
	N	6.06434	0.712	0.46972
	N	5.24139	-0.31459	0.26607
	N	-5.36849	1.55407	0.42441
	N	-6.04732	0.44769	0.42507
	N	-5.17837	-0.5437	0.23874
	C	-0.77105	3.17559	0.18203
	C	-1.20351	4.51278	0.01413
	C	1.05104	4.55645	0.03328
	C	0.6731	3.20382	0.19396
	C	1.63814	2.21248	0.29842
	H	1.32367	1.18185	0.43541
	C	2.99633	2.53399	0.23268
	C	3.36039	3.90708	0.10349
	C	2.40008	4.91638	0.00043
	H	2.74365	5.94295	-0.10187
	C	4.02119	1.48022	0.28062
	C	3.95221	0.09403	0.13283
	C	5.75899	-1.65245	0.20123
	C	6.16776	-2.14529	-1.04309
	C	6.65539	-3.45069	-1.08387
	H	6.98226	-3.86331	-2.0393
	C	6.7294	-4.24264	0.06903
	C	6.30456	-3.70556	1.28639
	H	6.35554	-4.31432	2.18947
	C	5.8076	-2.40192	1.37745

C	6.0662	-1.29071	-2.27661
H	6.47892	-1.81782	-3.14207
H	6.61009	-0.34644	-2.14658
H	5.01808	-1.04152	-2.49291
C	7.25703	-5.65028	-0.01869
H	6.63575	-6.25299	-0.69386
H	7.26579	-6.13197	0.96488
H	8.27935	-5.65668	-0.4186
C	5.32858	-1.82006	2.67905
H	4.25091	-1.60944	2.63782
H	5.84176	-0.8755	2.90003
H	5.50961	-2.51925	3.50101
C	-0.08417	6.79359	-0.23999
H	0.97482	7.06649	-0.27102
C	-0.70994	7.20316	-1.57076
H	-0.23172	6.6678	-2.39933
H	-0.56672	8.27972	-1.72276
H	-1.7858	6.99715	-1.59495
C	-0.71541	7.49735	0.95843
H	-0.22723	7.18211	1.88808
H	-1.78707	7.28145	1.03544
H	-0.59308	8.58179	0.85054
C	-2.56886	4.80773	-0.03956
H	-2.96169	5.81418	-0.14831
C	-3.48388	3.75613	0.05229
C	-3.06195	2.40131	0.19099
C	-1.69204	2.14224	0.27554
H	-1.33319	1.12722	0.42031
C	-4.03947	1.30348	0.23719
C	-3.90777	-0.08	0.1064
C	-5.6354	-1.90449	0.20668
C	-6.02825	-2.44553	-1.02244
C	-6.46062	-3.77098	-1.02758
H	-6.77371	-4.22132	-1.9705
C	-6.49563	-4.53583	0.1453
C	-6.08913	-3.95041	1.34647
H	-6.11081	-4.53767	2.26484
C	-5.64736	-2.62508	1.40186
C	-5.96951	-1.61853	-2.27732
H	-6.36069	-2.18551	-3.12748
H	-4.93449	-1.32765	-2.50411
H	-6.55526	-0.69684	-2.1686
C	-6.96379	-5.9662	0.09645
H	-6.32471	-6.55814	-0.57154
H	-7.98912	-6.02531	-0.29127
H	-6.94175	-6.42393	1.0912
C	-5.18863	-1.9907	2.68613
H	-4.11807	-1.74738	2.63919

	H	-5.34818	-2.67142	3.52781
	H	-5.73175	-1.05697	2.8798
	H	5.23307	3.53735	0.23592

Compound	Coordinates			
	Atom	X	Y	Z
**A_OH_mes_Br**	I	2.48404	-1.16121	-0.25831
Img. Freq. = 0	I	-2.38315	-1.27734	-0.26038
SCF Energy= -5130.37241981	Br	0.09596	-3.36488	-0.89927
	O	4.64757	4.45584	0.07075
	O	-4.79886	4.24275	-0.00946
	H	-5.34844	3.45449	0.18432
	N	-0.09608	5.45965	-0.1133
	N	5.33978	1.95071	0.54174
	N	6.08018	0.88451	0.5545
	N	5.27465	-0.15277	0.33803
	N	-5.38284	1.71296	0.48227
	N	-6.07376	0.61444	0.50553
	N	-5.22022	-0.38802	0.30934
	C	-0.77681	3.29759	0.16102
	C	-1.20803	4.63418	-0.02041
	C	1.04488	4.67912	0.00093
	C	0.66911	3.32661	0.17396
	C	1.64006	2.3416	0.29655
	H	1.33181	1.31005	0.44336
	C	2.99769	2.67092	0.24019
	C	3.3562	4.04435	0.09902
	C	2.39161	5.04617	-0.02528
	H	2.72996	6.0737	-0.13463
	C	4.03232	1.62791	0.31485
	C	3.98455	0.24088	0.17293
	C	5.80731	-1.48562	0.29541
	C	6.2531	-1.98197	-0.9347
	C	6.75247	-3.28343	-0.95462
	H	7.10778	-3.69893	-1.89857
	C	6.80206	-4.06802	0.20464
	C	6.34107	-3.52735	1.40719
	H	6.37344	-4.13032	2.31499
	C	5.83105	-2.22747	1.4772
	C	6.17663	-1.13515	-2.17536
	H	6.61315	-1.66475	-3.02752
	H	6.71176	-0.18698	-2.03772
	H	5.13273	-0.89295	-2.41844
	C	7.34198	-5.47219	0.13929
	H	6.73837	-6.08509	-0.54274
	H	7.33477	-5.94559	1.12689
	H	8.37188	-5.47426	-0.24063
	C	5.31252	-1.64249	2.76217

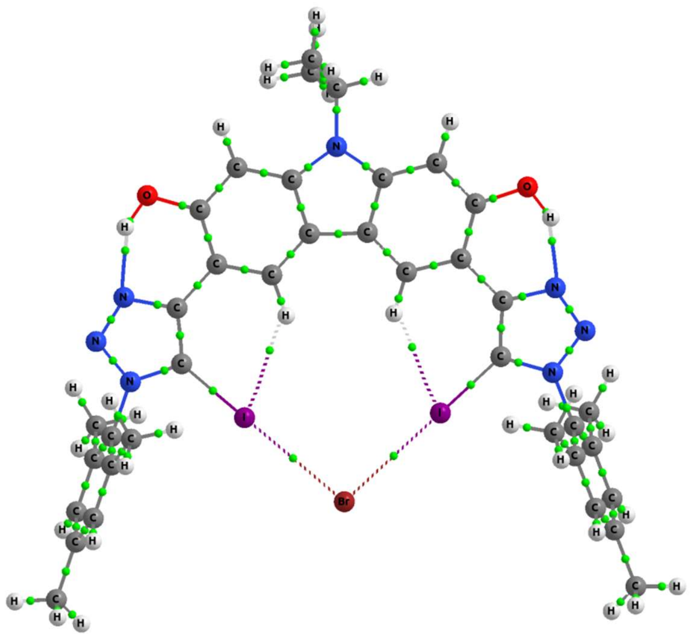

	H	4.23253	-1.45098	2.6949
	H	5.80439	-0.68744	2.9864
	H	5.48643	-2.33152	3.59419
	C	-0.09003	6.91234	-0.29854
	H	0.96888	7.18373	-0.33989
	C	-0.72343	7.31081	-1.62906
	H	-0.25246	6.76567	-2.45538
	H	-0.57687	8.38525	-1.79236
	H	-1.8002	7.10921	-1.64491
	C	-0.71161	7.62808	0.89789
	H	-0.21619	7.32158	1.82667
	H	-1.78274	7.41369	0.98561
	H	-0.58935	8.71132	0.77852
	C	-2.57173	4.93481	-0.07039
	H	-2.96015	5.94186	-0.1881
	C	-3.49077	3.8897	0.0422
	C	-3.07337	2.53505	0.19582
	C	-1.70334	2.26958	0.27259
	H	-1.34996	1.25397	0.42883
	C	-4.06034	1.44684	0.26962
	C	-3.94806	0.06158	0.14739
	C	-5.69147	-1.74439	0.29895
	C	-6.11278	-2.29327	-0.91721
	C	-6.55789	-3.61446	-0.90099
	H	-6.89362	-4.07063	-1.83326
	C	-6.57715	-4.36779	0.27965
	C	-6.14161	-3.77505	1.467
	H	-6.15084	-4.35337	2.39123
	C	-5.68658	-2.45357	1.50105
	C	-6.06825	-1.48	-2.18164
	H	-6.49525	-2.04583	-3.01517
	H	-5.03285	-1.21707	-2.43923
	H	-6.62845	-0.54331	-2.06731
	C	-7.05936	-5.79405	0.25375
	H	-6.43603	-6.39915	-0.41736
	H	-8.09098	-5.84774	-0.11766
	H	-7.02611	-6.2411	1.25302
	C	-5.19647	-1.81181	2.77001
	H	-4.12448	-1.5803	2.7004
	H	-5.34674	-2.48292	3.62105
	H	-5.72612	-0.87069	2.96518
	H	5.2293	3.69078	0.26466

Compound	Coordinates			
	Atom	X	Y	Z
**A_OH_mes_I**	I	2.54343	-1.02244	-0.24153
Img. Freq. = 0	I	-2.44573	-1.14579	-0.23323
SCF Energy= -2851.75490604	I	0.09991	-3.46392	-0.97815

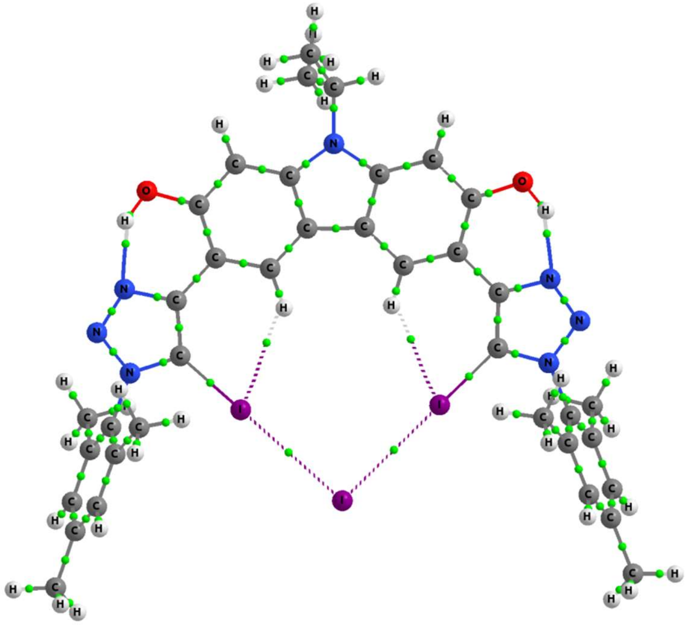

O	4.63519	4.6138	0.02999
O	-4.80936	4.38582	0.00029
H	-5.35875	3.60683	0.22863
N	-0.10942	5.58803	-0.18146
N	5.3376	2.13211	0.59157
N	6.08964	1.07499	0.63359
N	5.30159	0.02571	0.41306
N	-5.39258	1.87531	0.57423
N	-6.09211	0.78317	0.62243
N	-5.25326	-0.2281	0.411
C	-0.78609	3.43081	0.14389
C	-1.21888	4.7639	-0.06278
C	1.03264	4.81248	-0.0535
C	0.66119	3.4622	0.14927
C	1.63874	2.48566	0.29475
H	1.33842	1.45511	0.46588
C	2.99486	2.82282	0.23446
C	3.34659	4.19454	0.06326
C	2.3768	5.18687	-0.08642
H	2.7089	6.214	-0.21623
C	4.03865	1.79181	0.34108
C	4.01126	0.40347	0.21527
C	5.84843	-1.30211	0.40719
C	6.33798	-1.8129	-0.8001
C	6.84976	-3.10962	-0.78326
H	7.23864	-3.53652	-1.70869
C	6.86975	-3.87528	0.38951
C	6.36625	-3.32026	1.5682
H	6.37593	-3.90838	2.48619
C	5.84182	-2.02464	1.60111
C	6.29435	-0.98554	-2.05538
H	6.74958	-1.52986	-2.88823
H	6.82977	-0.03726	-1.91937
H	5.25774	-0.74315	-2.3277
C	7.4232	-5.27543	0.36359
H	6.8405	-5.90633	-0.32025
H	7.39683	-5.73004	1.35964
H	8.46149	-5.27559	0.0074
C	5.27724	-1.42499	2.85966
H	4.19672	-1.25238	2.75817
H	5.74738	-0.45832	3.08063
H	5.43761	-2.09637	3.70865
C	-0.10651	7.03705	-0.39459
H	0.95155	7.30745	-0.45648
C	-0.75763	7.41095	-1.72368
H	-0.30124	6.84665	-2.54527
H	-0.60777	8.48101	-1.91086
H	-1.83551	7.21541	-1.72053

	C	-0.71096	7.7754	0.79693
	H	-0.20004	7.48878	1.72367
	H	-1.77997	7.5603	0.90601
	H	-0.59331	8.85628	0.65387
	C	-2.5817	5.06775	-0.1048
	H	-2.96791	6.07351	-0.23898
	C	-3.50207	4.02902	0.04229
	C	-3.08569	2.67702	0.2198
	C	-1.71526	2.40794	0.28678
	H	-1.36496	1.39405	0.46264
	C	-4.07808	1.59684	0.32884
	C	-3.98236	0.21064	0.21293
	C	-5.73641	-1.58041	0.41484
	C	-6.19968	-2.12342	-0.78869
	C	-6.65366	-3.44134	-0.76106
	H	-7.02147	-3.89318	-1.68324
	C	-6.6417	-4.19685	0.41836
	C	-6.16518	-3.60972	1.59268
	H	-6.14996	-4.18978	2.51572
	C	-5.69966	-2.29162	1.61509
	C	-6.18972	-1.30685	-2.05173
	H	-6.63022	-1.87418	-2.87712
	H	-5.16286	-1.03333	-2.33149
	H	-6.75563	-0.37557	-1.9222
	C	-7.1338	-5.61986	0.40464
	H	-6.53171	-6.22852	-0.28247
	H	-8.17489	-5.6668	0.05978
	H	-7.07749	-6.06757	1.40255
	C	-5.16665	-1.65596	2.86972
	H	-4.09679	-1.42722	2.76688
	H	-5.29165	-2.32969	3.72275
	H	-5.68694	-0.71406	3.08522
	H	5.22057	3.86084	0.25678

Compound	Coordinates			
	Atom	X	Y	Z
**A_OH_per_Cl**	I	2.3603	-0.41862	-0.35417
Img. Freq. = 0	I	-2.32797	-0.46268	-0.48246
SCF Energy= -4030.15279797	Cl	0.05838	-2.28272	-1.24752
	N	-0.05332	6.16285	-0.03064
	N	5.31349	2.5376	0.69323
	N	6.0222	1.45916	0.68251
	N	5.18027	0.4508	0.41567
	N	-5.35988	2.41685	0.56107
	N	-6.04469	1.32301	0.52586
	N	-5.18278	0.3415	0.22618
	C	-0.76161	4.0171	0.28554
	C	-1.17675	5.35262	0.06623

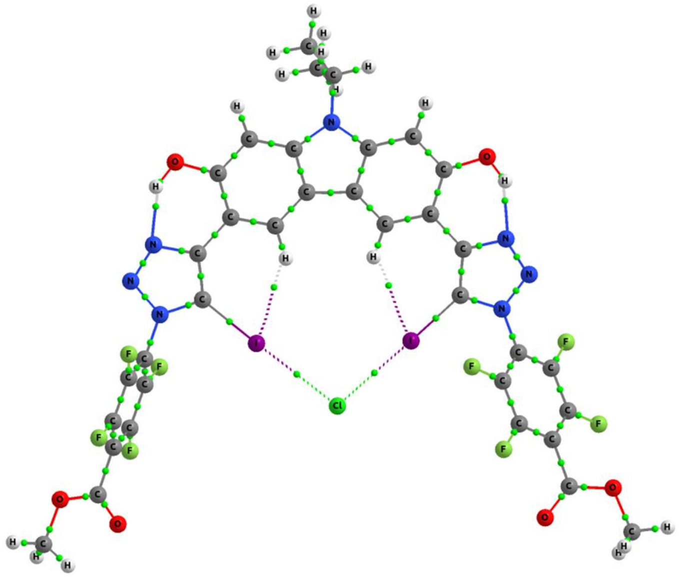

C	1.07757	5.37079	0.10589
C	0.68189	4.02917	0.3124
C	1.63326	3.03	0.452
H	1.30828	2.00883	0.6313
C	2.99449	3.33311	0.36908
C	3.37755	4.69563	0.20067
C	2.43112	5.71361	0.0665
H	2.78743	6.73231	-0.06627
C	3.99821	2.26223	0.43155
C	3.89982	0.88726	0.23553
C	5.68155	-0.86868	0.34446
C	6.5748	-1.22695	-0.65903
C	7.08751	-2.51558	-0.71234
C	6.71077	-3.48206	0.2167
C	5.80331	-3.11518	1.20851
C	5.30249	-1.82422	1.28222
C	-0.02777	7.61063	-0.25226
H	1.03479	7.86913	-0.28148
C	-0.63523	7.98119	-1.60264
H	-0.15712	7.41023	-2.40713
H	-0.47494	9.04956	-1.79075
H	-1.71374	7.78997	-1.63008
C	-0.66203	8.36172	0.9153
H	-0.18499	8.07446	1.85967
H	-1.73647	8.15935	0.99026
H	-0.52775	9.44039	0.77045
C	-2.53793	5.66167	-0.01612
H	-2.91759	6.66904	-0.15815
C	-3.4644	4.62205	0.08694
C	-3.05774	3.26838	0.26197
C	-1.69414	2.99593	0.38925
H	-1.35183	1.9815	0.57563
C	-4.04027	2.17643	0.28871
C	-3.91284	0.80983	0.05583
C	-5.64921	-0.99082	0.13688
C	-5.75517	-1.62609	-1.09587
C	-6.01313	-1.68261	1.2864
C	-6.20949	-2.93444	-1.17121
C	-6.48412	-2.98524	1.20047
C	-6.588	-3.63428	-0.02724
F	-5.42772	-0.97479	-2.20893
F	-6.31622	-3.49365	-2.37564
F	-6.79665	-3.61126	2.33596
F	-5.8986	-1.10147	2.47814
F	4.46564	-1.49604	2.26372
F	6.93358	-0.33884	-1.58291
F	7.92363	-2.81215	-1.70838
F	5.42924	-3.98771	2.14338

	O	-4.7709	4.97871	-0.00326
	H	-5.32997	4.20924	0.21582
	O	4.67727	5.08136	0.15033
	H	5.24796	4.31953	0.36922
	C	-7.05764	-5.0566	-0.14373
	O	-6.51894	-5.87232	-0.84861
	O	-8.12739	-5.27994	0.61093
	C	7.22499	-4.89218	0.1588
	O	6.52053	-5.85771	0.31377
	O	8.5326	-4.92488	-0.07197
	C	-8.62394	-6.6311	0.58172
	H	-7.8492	-7.32017	0.93324
	H	-9.48206	-6.63877	1.25595
	H	-8.92764	-6.89579	-0.43615
	C	9.10072	-6.24371	-0.17641
	H	8.62483	-6.7898	-0.99731
	H	10.1612	-6.0869	-0.38044
	H	8.96261	-6.78588	0.76456

	Atom	X	Y	Z
**A_OH_per_Br**	I	2.41797	-0.36935	-0.31622
Img. Freq. = 0	I	-2.38913	-0.40998	-0.44402
SCF Energy= -6144.19525429	Br	0.05542	-2.41968	-1.24052
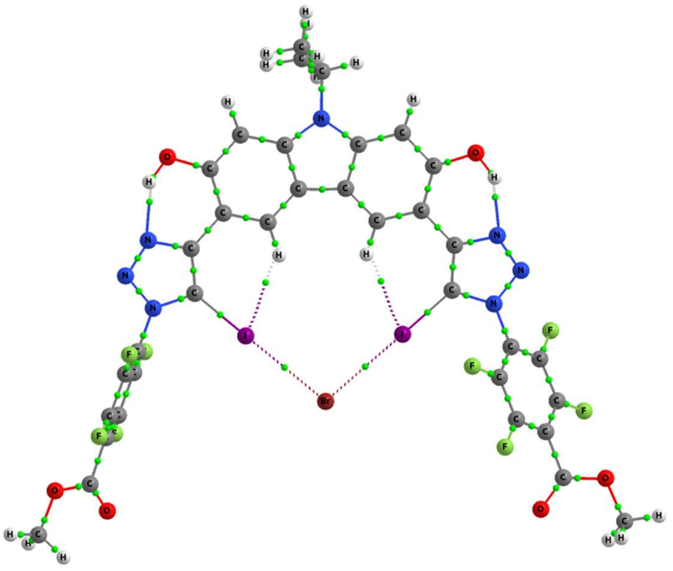	N	-0.05792	6.23432	-0.04318
	N	5.32592	2.64744	0.67498
	N	6.04822	1.57827	0.6824
	N	5.21936	0.55549	0.43315
	N	-5.37063	2.5186	0.60108
	N	-6.06845	1.43291	0.5818
	N	-5.22174	0.4394	0.28047
	C	-0.7638	4.08491	0.26086
	C	-1.17956	5.42324	0.05667
	C	1.07355	5.44259	0.08335
	C	0.68137	4.09772	0.27924
	C	1.63867	3.10269	0.41514
	H	1.31876	2.07846	0.58656
	C	2.99959	3.41384	0.34288
	C	3.37639	4.77926	0.18055
	C	2.42486	5.79202	0.04752
	H	2.77615	6.81327	-0.07832
	C	4.01338	2.35268	0.41883
	C	3.9336	0.97342	0.24759
	C	5.73636	-0.7594	0.38829
	C	6.61935	-1.13289	-0.61845
	C	7.14876	-2.41574	-0.64488
	C	6.79961	-3.36147	0.31602
	C	5.90209	-2.97932	1.31142
	C	5.38385	-1.69396	1.35685

	C	-0.03454	7.68447	-0.24992
	H	1.02761	7.94293	-0.28947
	C	-0.65699	8.06991	-1.58926
	H	-0.18941	7.50625	-2.40499
	H	-0.49602	9.13977	-1.76812
	H	-1.73619	7.88191	-1.60674
	C	-0.65389	8.42383	0.9331
	H	-0.16576	8.12614	1.86855
	H	-1.7276	8.22205	1.01905
	H	-0.51982	9.5038	0.79803
	C	-2.53986	5.73784	-0.00634
	H	-2.91703	6.74764	-0.13661
	C	-3.46922	4.70239	0.10579
	C	-3.06528	3.34612	0.26942
	C	-1.70009	3.06689	0.37158
	H	-1.36003	2.04958	0.54685
	C	-4.05644	2.26218	0.31546
	C	-3.9485	0.89253	0.09305
	C	-5.7056	-0.8874	0.20087
	C	-5.8234	-1.52788	-1.02817
	C	-6.07503	-1.56827	1.35519
	C	-6.29408	-2.83079	-1.09526
	C	-6.56268	-2.86535	1.2774
	C	-6.67797	-3.51973	0.05355
	F	-5.49073	-0.88668	-2.14557
	F	-6.41088	-3.395	-2.29635
	F	-6.88029	-3.48103	2.41707
	F	-5.95026	-0.98228	2.54348
	F	4.55479	-1.35095	2.33984
	F	6.95338	-0.2651	-1.57038
	F	7.9739	-2.7267	-1.64547
	F	5.55324	-3.8301	2.27542
	O	-4.77434	5.0674	0.03717
	H	-5.33506	4.30023	0.26184
	O	4.67294	5.17489	0.13702
	H	5.24881	4.41582	0.35424
	C	-7.1657	-4.93667	-0.05442
	O	-6.63887	-5.7626	-0.75634
	O	-8.23585	-5.14282	0.70451
	C	7.33051	-4.76634	0.28888
	O	6.64272	-5.73473	0.49316
	O	8.63195	-4.79106	0.0246
	C	-8.74941	-6.48778	0.68343
	H	-7.98279	-7.18467	1.03736
	H	-9.60634	-6.48105	1.35917
	H	-9.05821	-6.75406	-0.33247
	C	9.21331	-6.10585	-0.05463
	H	8.72116	-6.68326	-0.84393

	H	10.2656	-5.94335	-0.29398
	H	9.10876	-6.61957	0.90641

Compound	Coordinates			
	Atom	X	Y	Z
**A_OH_per_I**	I	2.4963	-0.25511	-0.40829
Img. Freq. = 0	I	-2.46902	-0.2926	-0.52313
SCF Energy= -3865.57741973	I	0.0565	-2.41394	-1.64691
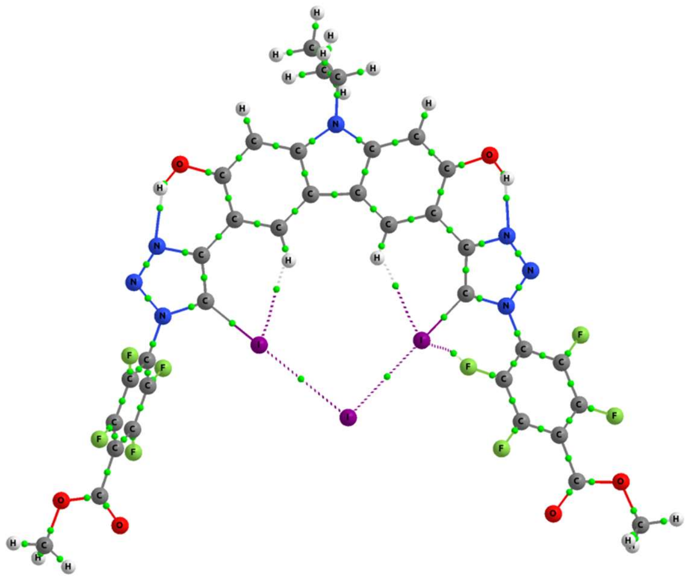	N	-0.04602	6.28003	-0.06373
	N	5.30967	2.71508	0.91548
	N	6.03441	1.64783	0.94475
	N	5.23275	0.62913	0.60332
	N	-5.35866	2.61083	0.79214
	N	-6.06409	1.52964	0.79622
	N	-5.24634	0.53544	0.42343
	C	-0.75932	4.14152	0.29859
	C	-1.16978	5.47599	0.05738
	C	1.08231	5.49037	0.09374
	C	0.68668	4.15111	0.32327
	C	1.6448	3.1617	0.49855
	H	1.32942	2.14036	0.69825
	C	3.00541	3.47651	0.43505
	C	3.38336	4.83668	0.24361
	C	2.43325	5.84282	0.06743
	H	2.78326	6.86193	-0.07766
	C	4.02181	2.42257	0.55289
	C	3.96345	1.05103	0.33255
	C	5.75713	-0.68227	0.55881
	C	6.75828	-1.00829	-0.34956
	C	7.28801	-2.29093	-0.37687
	C	6.82216	-3.28395	0.48109
	C	5.80972	-2.94833	1.37798
	C	5.29164	-1.66322	1.42911
	C	-0.01772	7.72399	-0.31065
	H	1.04516	7.97543	-0.36872
	C	-0.65072	8.07631	-1.65412
	H	-0.19712	7.48476	-2.45786
	H	-0.48071	9.13885	-1.86518
	H	-1.73183	7.89956	-1.65601
	C	-0.62127	8.4987	0.85781
	H	-0.12343	8.22588	1.79575
	H	-1.69454	8.30285	0.96151
	H	-0.48547	9.57397	0.69077
	C	-2.52806	5.79944	-0.00779
	H	-2.89782	6.80857	-0.1618
	C	-3.46293	4.77481	0.14064
	C	-3.06626	3.42187	0.33715
	C	-1.70302	3.13301	0.44034

	H	-1.3733	2.11713	0.64519
	C	-4.06701	2.34974	0.42061
	C	-3.98504	0.98601	0.16434
	C	-5.74032	-0.78837	0.36175
	C	-5.91255	-1.42771	-0.86148
	C	-6.06425	-1.4685	1.5303
	C	-6.38811	-2.72981	-0.90878
	C	-6.55945	-2.76364	1.47251
	C	-6.72616	-3.41783	0.25465
	F	-5.6298	-0.78569	-1.99204
	F	-6.55451	-3.2944	-2.10383
	F	-6.83114	-3.37921	2.62411
	F	-5.88755	-0.88392	2.71269
	F	4.35423	-1.3654	2.32592
	F	7.20408	-0.09648	-1.21015
	F	8.22946	-2.55478	-1.28385
	F	5.34556	-3.84567	2.24621
	O	-4.76747	5.14451	0.07311
	H	-5.3284	4.39869	0.35587
	O	4.68136	5.23091	0.21105
	H	5.25024	4.49113	0.49615
	C	-7.21887	-4.83453	0.16856
	O	-6.71738	-5.66346	-0.54817
	O	-8.26137	-5.03673	0.96591
	C	7.35001	-4.68983	0.4445
	O	6.64019	-5.6616	0.51091
	O	8.67403	-4.71174	0.34232
	C	-8.77409	-6.38221	0.97172
	H	-9.60528	-6.3722	1.67883
	H	-9.12019	-6.65475	-0.03038
	H	-7.99398	-7.07624	1.30087
	C	9.2585	-6.02563	0.27049
	H	8.86494	-6.56116	-0.59945
	H	10.3322	-5.85958	0.1685
	H	9.03732	-6.58352	1.18611

Compound	Coordinates			
	Atom	X	Y	Z
**B_H_mes_Cl**	I	2.33531	-0.95436	-0.70933
Img. Freq. = 0	I	-2.25445	-1.06795	-0.7218
SCF Energy: -2945.34207524	Cl	0.08068	-2.46692	-2.06198
	H	4.38728	4.04866	-0.15808
	H	-4.51889	3.84414	-0.20055
	N	-0.09185	5.24607	-0.28754
	N	5.18696	1.58101	1.19424
	N	5.85808	0.4602	1.17581
	N	5.05265	-0.409	0.61234
	N	-5.21133	1.35087	1.1719

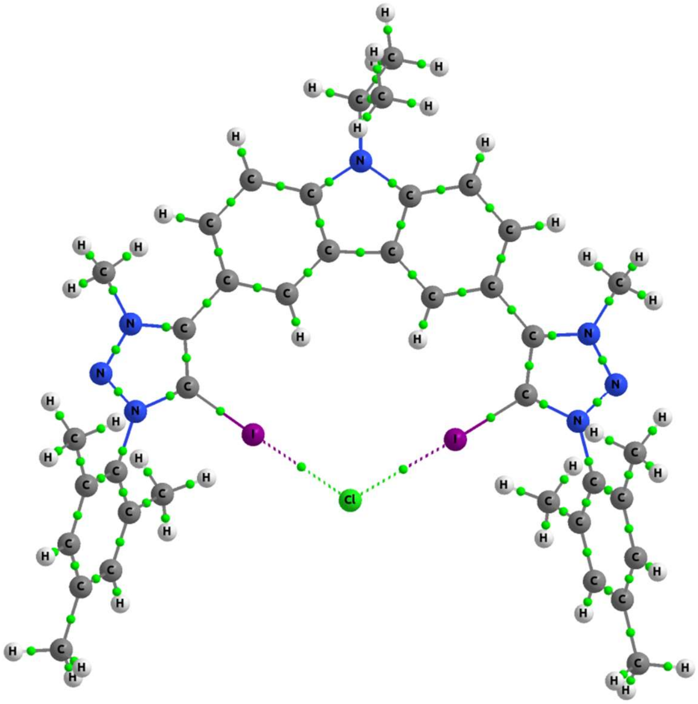

N	-5.83016	0.20047	1.16997
N	-4.9881	-0.63725	0.61241
C	-0.77237	3.1946	0.44148
C	-1.20086	4.45634	-0.0406
C	1.04498	4.50375	-0.02547
C	0.66807	3.22534	0.45129
C	1.6228	2.2542	0.72992
H	1.31933	1.27757	1.10208
C	2.96568	2.54848	0.49975
C	3.33964	3.8322	0.04064
C	2.39959	4.82181	-0.21085
H	2.72327	5.79264	-0.57776
C	3.94546	1.46611	0.64198
C	3.85811	0.13249	0.25517
C	5.48833	-1.77344	0.43276
C	6.11508	-2.10667	-0.77195
C	6.50612	-3.43512	-0.92845
H	7.00084	-3.7337	-1.85331
C	6.27495	-4.39144	0.06899
C	5.63744	-4.00116	1.24943
H	5.45175	-4.73886	2.03001
C	5.22502	-2.68238	1.45713
C	6.34016	-1.07188	-1.83978
H	6.90025	-1.50337	-2.67428
H	6.90301	-0.21551	-1.44664
H	5.38358	-0.69589	-2.22868
C	6.70902	-5.81645	-0.1442
H	6.20862	-6.24183	-1.02364
H	6.47071	-6.43767	0.72532
H	7.79046	-5.86718	-0.32474
C	4.52228	-2.25247	2.71522
H	3.5093	-1.8899	2.49168
H	5.06707	-1.44096	3.21467
H	4.43822	-3.0936	3.40923
C	-0.08492	6.61638	-0.81721
H	0.97297	6.89006	-0.86025
C	-0.64341	6.66573	-2.23613
H	-0.12739	5.94266	-2.87844
H	-0.48996	7.66991	-2.64824
H	-1.71795	6.45178	-2.25629
C	-0.78112	7.58128	0.1372
H	-0.34631	7.51126	1.14093
H	-1.85646	7.38166	0.20415
H	-0.6495	8.60601	-0.22922
C	-2.56878	4.70622	-0.24094
H	-2.93656	5.65712	-0.61395
C	-3.46385	3.67452	0.00534
C	-3.03598	2.41012	0.46956

	C	-1.68305	2.17915	0.71106
	H	-1.33673	1.21803	1.08625
	C	-3.96777	1.28637	0.61599
	C	-3.82173	-0.04555	0.2418
	C	-5.35605	-2.02475	0.46344
	C	-6.02856	-2.40264	-0.7026
	C	-6.35158	-3.75218	-0.83004
	H	-6.87827	-4.08598	-1.72475
	C	-6.01184	-4.6855	0.15815
	C	-5.33397	-4.24995	1.29967
	H	-5.0649	-4.96925	2.07304
	C	-4.98563	-2.90836	1.47702
	C	-6.37117	-1.3904	-1.7608
	H	-6.92699	-1.86517	-2.57436
	H	-5.46068	-0.9431	-2.18326
	H	-6.98241	-0.57831	-1.34642
	C	-6.3737	-6.13477	-0.02462
	H	-5.87663	-6.54373	-0.9138
	H	-7.45543	-6.24701	-0.17204
	H	-6.07562	-6.73088	0.84405
	C	-4.2418	-2.4285	2.69317
	H	-3.24549	-2.05334	2.4204
	H	-4.1126	-3.24762	3.40639
	H	-4.78272	-1.61378	3.19142
	C	5.81501	2.75953	1.7905
	H	6.28348	3.35685	1.00366
	H	5.0426	3.33505	2.30516
	H	6.56542	2.40166	2.49702
	C	-5.88733	2.50667	1.75968
	H	-5.1408	3.11098	2.27975
	H	-6.3695	3.08509	0.96704
	H	-6.63086	2.12308	2.46002

Compound	Coordinates			
	Atom	X	Y	Z
**B_H_mes_Br**	I	2.41201	-0.87808	-0.65622
Img. Freq. = 0	I	-2.34511	-0.98076	-0.6677
SCF Energy: -5059.38415001	Br	0.0684	-2.49529	-2.13744
	H	4.41111	4.12999	-0.1923
	H	-4.50077	3.92411	-0.19777
	N	-0.07054	5.31537	-0.35261
	N	5.20581	1.70774	1.25846
	N	5.87788	0.5877	1.29325
	N	5.087	-0.30081	0.73928
	N	-5.16752	1.50315	1.33439
	N	-5.81096	0.36681	1.37143
	N	-5.01653	-0.49284	0.77745
	C	-0.74846	3.28245	0.42958

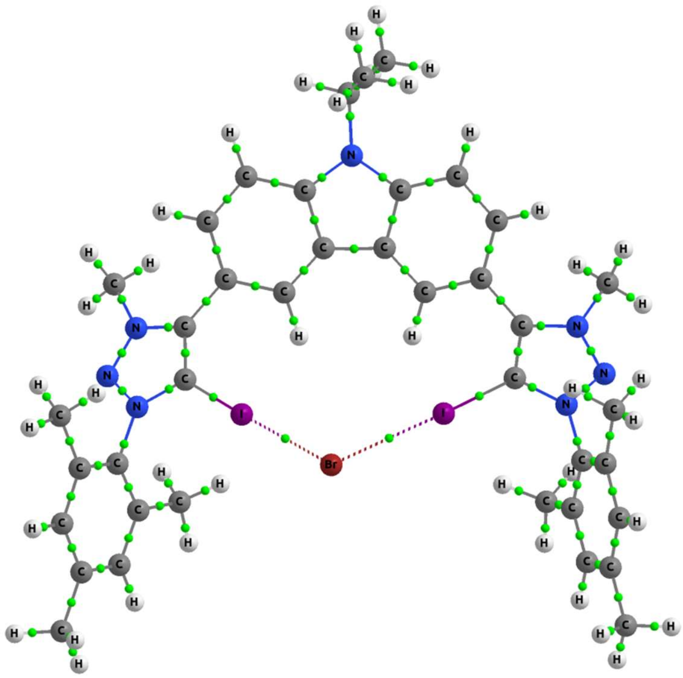

C	-1.17869	4.53294	-0.08049
C	1.06707	4.57957	-0.07749
C	0.69231	3.31194	0.42965
C	1.64971	2.35002	0.73162
H	1.34922	1.38167	1.12732
C	2.99216	2.64408	0.49801
C	3.36321	3.9168	0.008
C	2.42079	4.8965	-0.27073
H	2.74185	5.85896	-0.66106
C	3.97694	1.57264	0.68269
C	3.90223	0.22838	0.33476
C	5.52135	-1.67242	0.62234
C	6.23275	-2.03802	-0.52443
C	6.61786	-3.3736	-0.62371
H	7.17597	-3.69715	-1.50298
C	6.30127	-4.30573	0.37327
C	5.58204	-3.88329	1.49419
H	5.33012	-4.60164	2.2742
C	5.17081	-2.55604	1.64304
C	6.55049	-1.02803	-1.59236
H	7.13626	-1.49174	-2.39111
H	7.12366	-0.18713	-1.18103
H	5.62952	-0.62277	-2.03386
C	6.73173	-5.73973	0.22118
H	6.27424	-6.1836	-0.67248
H	6.44104	-6.33635	1.09202
H	7.82051	-5.80486	0.09978
C	4.38138	-2.09184	2.83619
H	3.37835	-1.75814	2.53575
H	4.87904	-1.25172	3.33737
H	4.26663	-2.90727	3.55606
C	-0.06564	6.67734	-0.90354
H	0.99203	6.95068	-0.95338
C	-0.62793	6.70558	-2.32153
H	-0.11645	5.97022	-2.95338
H	-0.47157	7.70236	-2.75021
H	-1.70339	6.496	-2.33565
C	-0.75905	7.65647	0.03842
H	-0.31954	7.6032	1.04117
H	-1.83381	7.45679	0.1138
H	-0.63026	8.67535	-0.34488
C	-2.54713	4.78192	-0.27911
H	-2.91337	5.72162	-0.68075
C	-3.44394	3.76259	0.00799
C	-3.0151	2.51378	0.51084
C	-1.66087	2.27954	0.73936
H	-1.31762	1.32664	1.13815
C	-3.95718	1.40823	0.71442

	C	-3.85814	0.07213	0.34562
	C	-5.4244	-1.87061	0.64188
	C	-6.13889	-2.22976	-0.5051
	C	-6.49785	-3.57083	-0.62523
	H	-7.05759	-3.88987	-1.50513
	C	-6.15268	-4.51405	0.35171
	C	-5.43182	-4.09734	1.47375
	H	-5.15725	-4.82464	2.23765
	C	-5.04628	-2.76481	1.64311
	C	-6.48708	-1.2066	-1.55097
	H	-7.07915	-1.66478	-2.3483
	H	-5.57872	-0.78151	-2.00019
	H	-7.06478	-0.38065	-1.11619
	C	-6.55571	-5.95345	0.17729
	H	-6.10938	-6.36778	-0.7359
	H	-7.64541	-6.03952	0.07866
	H	-6.23305	-6.56229	1.02817
	C	-4.2544	-2.30529	2.83644
	H	-3.26283	-1.94264	2.53149
	H	-4.11295	-3.13112	3.53944
	H	-4.76557	-1.48639	3.35856
	C	5.82023	2.90917	1.82227
	H	6.29827	3.48059	1.02205
	H	5.03796	3.49929	2.30434
	H	6.56128	2.57972	2.55206
	C	-5.77342	2.67809	1.96008
	H	-4.98165	3.24652	2.45307
	H	-6.26526	3.285	1.19566
	H	-6.50084	2.31609	2.68811

Compound	Coordinates			
	Atom	X	Y	Z
**B_H_mes_I**	I	2.52879	-0.81991	-0.56679
Img. Freq. = 0	I	-2.44678	-0.93044	-0.55345
SCF Energy: -2780.76597171	I	0.07454	-2.65524	-2.09799
	H	4.39856	4.21963	-0.23206
	H	-4.5205	4.03035	-0.23078
	N	-0.08489	5.3812	-0.46403
	N	5.18389	1.89519	1.35607
	N	5.89001	0.79882	1.43831
	N	5.14848	-0.12664	0.87696
	N	-5.20112	1.68257	1.37416
	N	-5.86555	0.56091	1.45915
	N	-5.09214	-0.33627	0.89428
	C	-0.76616	3.36812	0.36698
	C	-1.19351	4.60858	-0.17049
	C	1.05127	4.65188	-0.16866
	C	0.67564	3.39584	0.36752

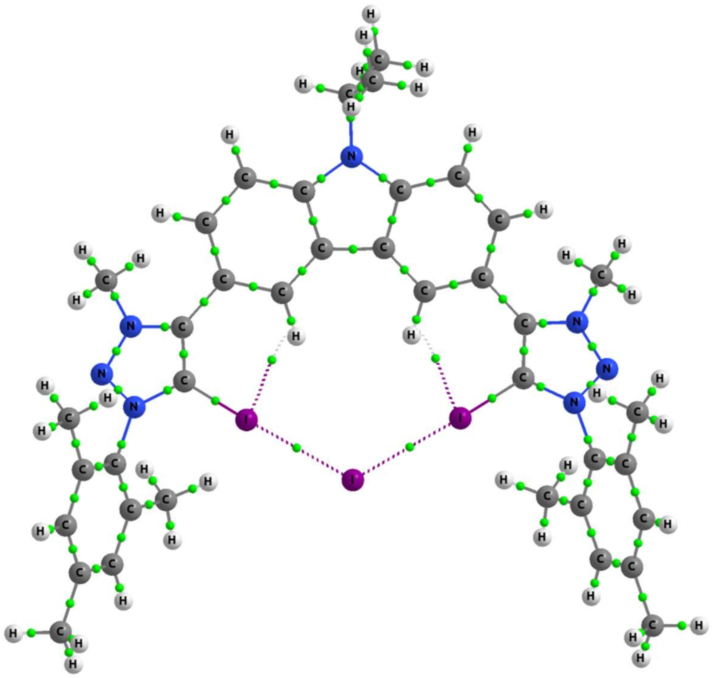

C	1.63578	2.44867	0.70906
H	1.33708	1.48909	1.12822
C	2.97952	2.74973	0.49065
C	3.3492	4.00695	-0.03814
C	2.40452	4.96994	-0.36129
H	2.72357	5.92133	-0.7793
C	3.98186	1.70817	0.73921
C	3.96259	0.35581	0.4217
C	5.63445	-1.4833	0.79311
C	6.36622	-1.84464	-0.34215
C	6.80297	-3.16606	-0.41155
H	7.37853	-3.48606	-1.2808
C	6.51615	-4.0887	0.6032
C	5.77395	-3.67134	1.71106
H	5.54418	-4.38299	2.50392
C	5.3116	-2.35804	1.83032
C	6.65054	-0.84495	-1.42902
H	7.26737	-1.29832	-2.21009
H	7.17844	0.03108	-1.03032
H	5.71746	-0.49325	-1.89083
C	7.00206	-5.50792	0.48353
H	6.56506	-5.98831	-0.40147
H	6.73157	-6.09624	1.36645
H	8.09293	-5.53338	0.36637
C	4.49814	-1.89813	3.0088
H	3.49739	-1.57424	2.69052
H	4.97997	-1.0515	3.51468
H	4.37929	-2.71179	3.72997
C	-0.07829	6.73447	-1.03645
H	0.97978	7.00424	-1.09396
C	-0.6454	6.74267	-2.4528
H	-0.14067	5.99382	-3.0741
H	-0.4841	7.73123	-2.89836
H	-1.72221	6.54009	-2.46031
C	-0.76486	7.73057	-0.10724
H	-0.3203	7.69275	0.89402
H	-1.83976	7.53555	-0.02289
H	-0.63488	8.74264	-0.50783
C	-2.56133	4.86289	-0.36378
H	-2.92402	5.79385	-0.78799
C	-3.4633	3.86114	-0.03644
C	-3.03962	2.62299	0.49604
C	-1.68424	2.38099	0.71179
H	-1.34469	1.43618	1.1322
C	-3.99614	1.54092	0.7516
C	-3.92623	0.18995	0.43501
C	-5.53126	-1.70888	0.80913
C	-6.25736	-2.09197	-0.32287

	C	-6.65163	-3.42668	-0.39221
	H	-7.22191	-3.76336	-1.25864
	C	-6.32993	-4.34137	0.61919
	C	-5.59561	-3.90204	1.72371
	H	-5.33919	-4.60701	2.51434
	C	-5.17522	-2.57467	1.843
	C	-6.58226	-1.10109	-1.4065
	H	-7.19604	-1.57218	-2.17947
	H	-5.6653	-0.7243	-1.88049
	H	-7.12929	-0.23963	-1.00204
	C	-6.77236	-5.77485	0.50061
	H	-6.33146	-6.23892	-0.39109
	H	-7.8633	-5.83398	0.39655
	H	-6.47333	-6.35696	1.37844
	C	-4.37304	-2.09102	3.01971
	H	-3.38472	-1.7336	2.69894
	H	-4.22534	-2.90192	3.7386
	H	-4.88073	-1.2618	3.52907
	C	5.73336	3.12661	1.92352
	H	6.24162	3.69204	1.13802
	H	4.9093	3.70669	2.34412
	H	6.43892	2.83559	2.70323
	C	-5.78929	2.89254	1.94818
	H	-4.9845	3.49095	2.38056
	H	-6.30706	3.45179	1.16455
	H	-6.49231	2.57434	2.71938

Compound	Coordinates			
	Atom	X	Y	Z
**B_H_per_Cl**	I	2.30507	-0.10196	-0.71389
Img. Freq. = 0	I	-2.24175	-0.12709	-0.80768
SCF Energy: -3959.15292259	Cl	0.07038	-1.49334	-2.12621
	H	4.47701	4.82983	-0.06043
	H	-4.43073	4.79449	-0.2232
	N	0.02367	6.11173	-0.2466
	N	5.20503	2.32321	1.2834
	N	5.84511	1.19081	1.2796
	N	5.01717	0.34511	0.70039
	N	-5.1755	2.29838	1.13458
	N	-5.82311	1.17094	1.10782
	N	-4.99574	0.32835	0.52314
	C	-0.70526	4.07064	0.46256
	C	-1.1033	5.34254	-0.01847
	C	1.14246	5.34739	0.02803
	C	0.73468	4.0743	0.49384
	C	1.6655	3.08346	0.78093
	H	1.33784	2.1122	1.14657
	C	3.01657	3.35235	0.56757

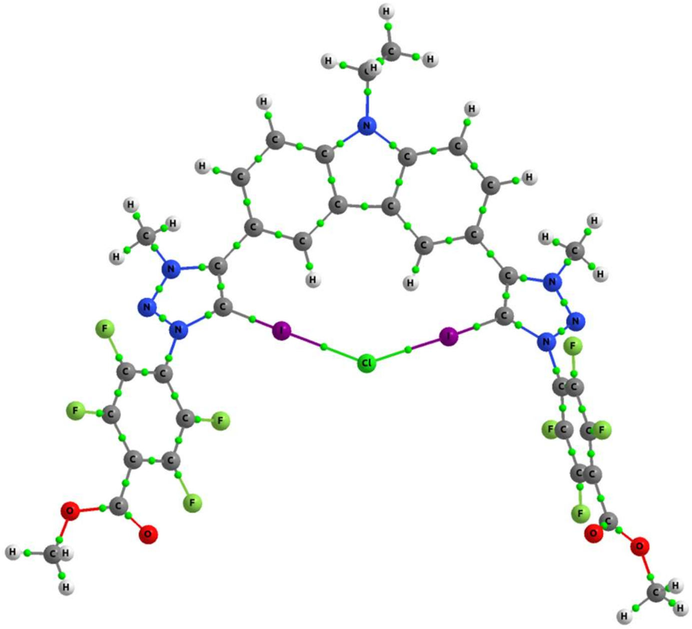

C	3.42284	4.63117	0.12189
C	2.50565	5.64024	-0.13592
H	2.85301	6.60705	-0.4918
C	3.96623	2.24453	0.71315
C	3.83982	0.91729	0.31746
C	5.43105	-1.00385	0.52891
C	6.50242	-1.30112	-0.30474
C	6.90548	-2.61922	-0.46798
C	6.24148	-3.66035	0.17503
C	5.16223	-3.34539	0.9977
C	4.76372	-2.03045	1.18729
C	0.06189	7.48358	-0.77212
H	1.1252	7.73657	-0.80505
C	-0.48372	7.54689	-2.19538
H	0.02537	6.81749	-2.83603
H	-0.30991	8.54975	-2.6025
H	-1.56143	7.35116	-2.2254
C	-0.62527	8.45769	0.17934
H	-0.20131	8.37637	1.18684
H	-1.70462	8.27697	0.23507
H	-0.47187	9.48108	-0.18224
C	-2.46362	5.6204	-0.23438
H	-2.80858	6.58115	-0.60393
C	-3.38115	4.60444	-0.00743
C	-2.98128	3.32885	0.45075
C	-1.63716	3.06974	0.70978
H	-1.31351	2.10022	1.08347
C	-3.93443	2.22074	0.56981
C	-3.81104	0.89764	0.16057
C	-5.41247	-1.01952	0.34668
C	-5.6762	-1.5085	-0.92719
C	-5.56613	-1.84925	1.45035
C	-6.08676	-2.82386	-1.08933
C	-5.99516	-3.15774	1.27683
C	-6.26163	-3.66565	0.00747
F	-5.54781	-0.71261	-1.98397
F	-6.35193	-3.24752	-2.32133
F	-6.11209	-3.921	2.36127
F	-5.29657	-1.39248	2.66948
F	3.75333	-1.75046	2.00518
F	7.13232	-0.32711	-0.95398
F	7.92418	-2.86097	-1.29041
F	4.51022	-4.29861	1.6573
C	-6.69619	-5.09008	-0.20917
O	-6.24518	-5.78594	-1.08277
O	-7.62305	-5.45189	0.66733
C	6.63327	-5.10033	-0.02121
O	5.82913	-5.97276	-0.22854

	O	7.9459	-5.2623	0.07236
	C	-8.07589	-6.81542	0.55063
	H	-7.23051	-7.49912	0.6774
	H	-8.80428	-6.9474	1.35227
	H	-8.54182	-6.9703	-0.42768
	C	8.41223	-6.61036	-0.13651
	H	8.1289	-6.94926	-1.13804
	H	9.49757	-6.55872	-0.03791
	H	7.98402	-7.27414	0.62107
	C	5.8555	3.4874	1.88695
	H	6.33087	4.07778	1.0991
	H	5.09164	4.07169	2.40445
	H	6.60084	3.11123	2.58918
	C	-5.82286	3.4593	1.74794
	H	-5.05808	4.03374	2.27505
	H	-6.29145	4.06096	0.96458
	H	-6.57287	3.07896	2.443

Compound	Coordinates			
	Atom	X	Y	Z
**B_H_per_Br**	I	2.40091	-0.08264	-0.752
Img. Freq. = 0	I	-2.3312	-0.06734	-0.72461
SCF Energy: -6073.19515894	Br	0.02266	-1.56127	-2.22877
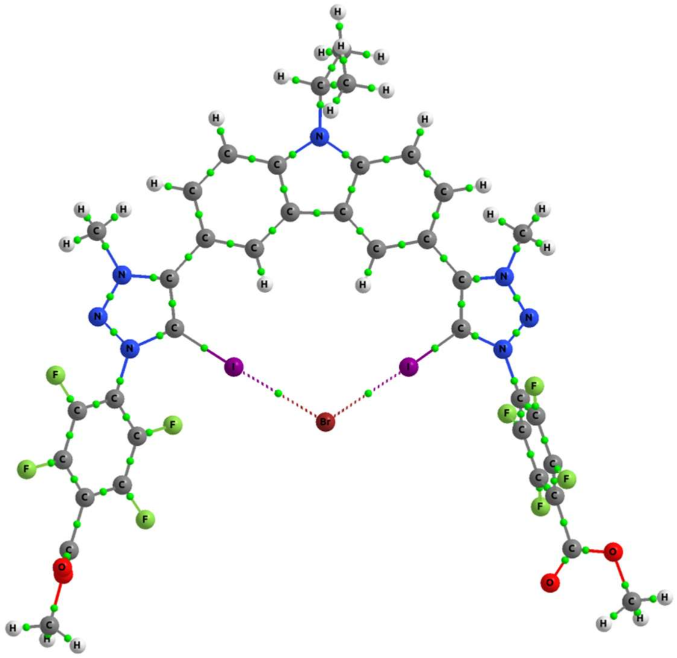	H	4.52452	4.86002	-0.21513
	H	-4.38881	4.84272	-0.00837
	N	0.06576	6.13884	-0.24764
	N	5.29266	2.39058	1.18825
	N	5.93913	1.26186	1.2064
	N	5.11369	0.39856	0.65022
	N	-5.06397	2.37992	1.45762
	N	-5.74245	1.27044	1.45742
	N	-4.98769	0.42178	0.78822
	C	-0.63683	4.10308	0.50451
	C	-1.05178	5.37448	0.03556
	C	1.19352	5.37366	-0.01726
	C	0.80345	4.10308	0.47096
	C	1.74604	3.11624	0.73278
	H	1.43393	2.14821	1.12015
	C	3.08904	3.38835	0.47638
	C	3.47709	4.66234	0.00274
	C	2.54922	5.6663	-0.23485
	H	2.882	6.63018	-0.61173
	C	4.05115	2.29209	0.62658
	C	3.93234	0.95653	0.26015
	C	5.52888	-0.95457	0.51789
	C	6.59679	-1.27797	-0.31072
	C	6.9923	-2.6018	-0.44021
	C	6.34233	-3.62061	0.25226

	C	5.27481	-3.2785	1.07895
	C	4.86737	-1.95914	1.21447
	C	0.08393	7.51124	-0.77311
	H	1.14527	7.76323	-0.84968
	C	-0.51861	7.5781	-2.1733
	H	-0.04212	6.84387	-2.83319
	H	-0.3518	8.57909	-2.58793
	H	-1.59851	7.39309	-2.16067
	C	-0.56145	8.4851	0.20762
	H	-0.09106	8.40571	1.19448
	H	-1.63657	8.30201	0.31395
	H	-0.42693	9.50833	-0.16177
	C	-2.41886	5.65935	-0.11911
	H	-2.77393	6.61841	-0.48316
	C	-3.33032	4.65192	0.16181
	C	-2.91508	3.379	0.61337
	C	-1.56191	3.10953	0.80584
	H	-1.22835	2.13877	1.16912
	C	-3.87672	2.28609	0.79024
	C	-3.82076	0.97155	0.34605
	C	-5.44905	-0.91165	0.61373
	C	-5.77763	-1.37683	-0.65404
	C	-5.57479	-1.75355	1.71238
	C	-6.23256	-2.67793	-0.8159
	C	-6.02643	-3.05366	1.53652
	C	-6.36552	-3.53463	0.27413
	F	-5.65954	-0.57478	-1.70761
	F	-6.51869	-3.08851	-2.04895
	F	-6.15155	-3.82346	2.6142
	F	-5.26305	-1.31439	2.92769
	F	3.84696	-1.65543	2.01153
	F	7.23626	-0.32323	-0.97834
	F	8.02902	-2.86899	-1.22942
	F	4.59913	-4.20942	1.74806
	C	-6.8238	-4.95994	0.11901
	O	-6.27679	-5.88409	0.66474
	O	-7.88457	-5.04442	-0.67126
	C	6.78215	-5.04742	0.06301
	O	7.03989	-5.51342	-1.0175
	O	6.84996	-5.69109	1.21998
	C	7.22979	-7.07865	1.12652
	H	8.23372	-7.16177	0.69849
	H	7.21313	-7.45221	2.15151
	H	6.50891	-7.61873	0.50452
	C	-8.37151	-6.38168	-0.90186
	H	-9.22238	-6.26676	-1.5751
	H	-7.58695	-6.98652	-1.36753
	H	-8.68276	-6.83309	0.04542

	C	-5.61153	3.54152	2.16045
	H	-4.78535	4.05252	2.65971
	H	-6.09641	4.20208	1.43754
	H	-6.33457	3.16646	2.8862
	C	5.93979	3.57272	1.76035
	H	6.40288	4.14995	0.95566
	H	5.17607	4.16177	2.27272
	H	6.69468	3.21741	2.46315

Compound	Coordinates			
	Atom	X	Y	Z
**B_H_per_I**	I	-2.49699	-0.03173	0.67191
Img. Freq. = 0	I	2.4538	-0.05803	0.78981
SCF Energy: -3794.57641568	I	-0.07168	-1.62455	2.3999
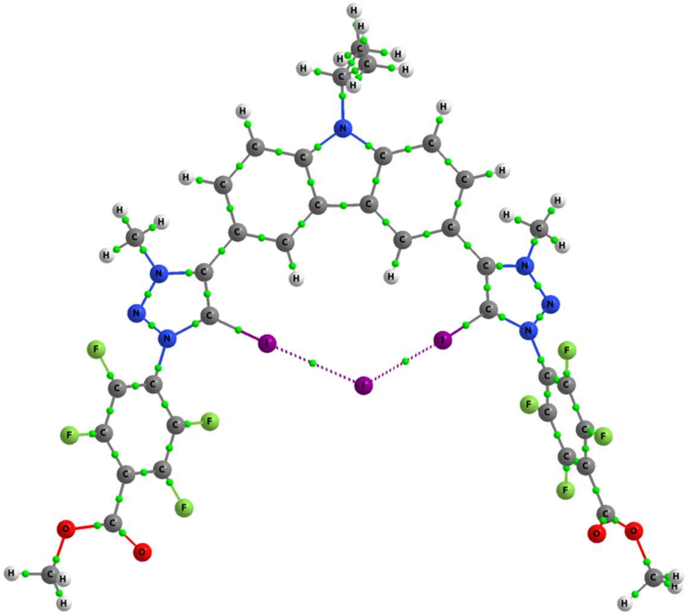	H	-4.46686	4.89708	-0.06087
	H	4.45498	4.86624	0.17889
	N	-0.00997	6.15035	0.24243
	N	-5.146	2.4576	-1.56426
	N	-5.82641	1.34966	-1.60585
	N	-5.08501	0.48166	-0.94708
	N	5.17332	2.43493	-1.3529
	N	5.8548	1.32771	-1.37981
	N	5.10346	0.46084	-0.73031
	C	0.72663	4.11454	-0.47563
	C	1.1193	5.38548	0.01399
	C	-1.12562	5.38922	-0.05122
	C	-0.71367	4.11724	-0.51836
	C	-1.64528	3.13663	-0.84066
	H	-1.31818	2.16493	-1.20737
	C	-2.99896	3.42176	-0.67048
	C	-3.40817	4.69558	-0.21477
	C	-2.48985	5.69074	0.08665
	H	-2.83729	6.65509	0.44892
	C	-3.96726	2.34176	-0.88458
	C	-3.92564	1.01746	-0.46998
	C	-5.5627	-0.84806	-0.79018
	C	-6.69488	-1.0922	-0.02233
	C	-7.16062	-2.39122	0.127
	C	-6.50053	-3.46484	-0.46468
	C	-5.36038	-3.20301	-1.22124
	C	-4.89623	-1.90749	-1.39492
	C	-0.05282	7.52334	0.76504
	H	-1.11659	7.77443	0.79371
	C	0.48797	7.59068	2.18983
	H	-0.02309	6.86289	2.83068
	H	0.31251	8.5946	2.59361
	H	1.56559	7.39529	2.22421
	C	0.63453	8.49765	-0.18628

	H	0.21616	8.41068	-1.19568
	H	1.71515	8.32324	-0.23576
	H	0.47326	9.52137	0.1709
	C	2.47803	5.67328	0.22665
	H	2.81556	6.63182	0.60845
	C	3.40391	4.67221	-0.02837
	C	3.01126	3.40204	-0.50599
	C	1.6668	3.12615	-0.74541
	H	1.35174	2.15712	-1.12937
	C	3.98626	2.32161	-0.68773
	C	3.93671	0.99781	-0.27341
	C	5.56463	-0.8776	-0.59847
	C	5.90858	-1.37948	0.6512
	C	5.67702	-1.68665	-1.72268
	C	6.35731	-2.68705	0.76917
	C	6.14484	-2.98691	-1.59379
	C	6.49086	-3.50735	-0.34921
	F	5.82012	-0.60274	1.72619
	F	6.69783	-3.12436	1.97764
	F	6.21936	-3.73215	-2.69445
	F	5.33149	-1.2183	-2.9181
	F	-3.82292	-1.67657	-2.145
	F	-7.32285	-0.08619	0.57796
	F	-8.23677	-2.58286	0.88677
	F	-4.70827	-4.18868	-1.83065
	C	-6.95799	-4.88636	-0.27693
	O	-6.20017	-5.78517	-0.01507
	O	-8.26848	-5.00064	-0.44206
	C	6.96721	-4.92494	-0.17972
	O	6.5652	-5.64932	0.69451
	O	7.86935	-5.24513	-1.09709
	C	8.35869	-6.59934	-1.02682
	H	7.52603	-7.30071	-1.14055
	H	9.06193	-6.69549	-1.85552
	H	8.86154	-6.76396	-0.0686
	C	-8.79559	-6.32797	-0.24433
	H	-8.58091	-6.66341	0.77532
	H	-9.87115	-6.23811	-0.40383
	H	-8.3509	-7.01689	-0.96933
	C	5.71901	3.61604	-2.0245
	H	4.89519	4.12845	-2.52632
	H	6.18566	4.26615	-1.28026
	H	6.4566	3.26266	-2.74628
	C	-5.68246	3.6399	-2.24108
	H	-6.17262	4.28165	-1.50477
	H	-4.84985	4.16142	-2.7184
	H	-6.39987	3.28735	-2.98331

Compound	Coordinates

	Atom	X	Y	Z
**C_H_PhCO2Me_Cl**	I	1.89645	-0.29641	-0.73253
Img. Freq. = 0	I	-1.99923	-0.2498	-0.74849
SCF Energy= -2738.40315457	Cl	-0.07224	-2.38248	-2.0552
	H	6.52653	1.41881	-0.7781
	H	4.04053	-1.13742	1.63739
	H	-8.3433	-0.18555	-1.00371
	H	8.23586	-0.38753	-0.93568
	H	5.73847	-2.95934	1.44876
	H	-4.1823	-1.04376	1.58536
	H	-6.58928	1.59113	-0.82591
	H	-5.90895	-2.82077	1.37675
	O	-8.00668	-3.78229	0.64824
	O	-9.32786	-2.46189	-0.59672
	N	4.61237	2.47872	0.98777
	N	4.23541	1.27551	0.52431
	N	-4.30916	1.38028	0.48756
	O	9.15482	-2.59255	-0.54437
	N	-3.60627	3.34053	0.93753
	N	0.01407	5.22521	-0.09642
	O	7.91205	-3.97968	0.70891
	N	-4.66063	2.59309	0.94596
	N	3.5766	3.25166	0.97151
	C	-0.02202	2.55127	0.50092
	H	-0.0361	1.5065	0.80455
	C	-8.30167	-2.6409	0.02221
	C	-1.22368	3.24396	0.32625
	C	-5.087	-0.87112	1.00545
	C	2.91864	1.27052	0.19895
	C	-7.42847	-0.36722	-0.44367
	C	-7.22948	-1.60829	0.1677
	C	-5.29817	0.35429	0.37778
	C	-1.16765	4.60113	0.02683
	H	-2.05648	5.20987	-0.11703
	C	1.19799	3.21415	0.33844
	C	5.90302	-2.00083	0.96139
	C	7.31817	-0.55564	-0.37768
	C	5.19861	0.22397	0.4261
	C	1.17869	4.57224	0.03855
	H	2.0844	5.15778	-0.09741
	C	4.95263	-0.99146	1.06185
	C	6.36874	0.45845	-0.29131
	C	7.08341	-1.78598	0.24358
	C	2.5019	2.55929	0.4991
	C	-2.99025	1.34282	0.17307
	C	-2.54475	2.62156	0.47511
	C	-6.05984	-1.85956	0.89205
	C	8.07319	-2.90509	0.17282

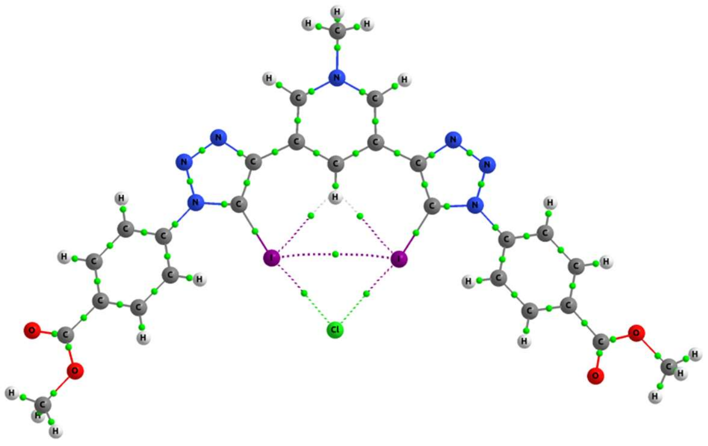

	C	-6.45813	0.62375	-0.34519
	C	-8.99952	-4.81456	0.5458
	H	-8.60051	-5.66223	1.10606
	H	-9.15361	-5.08674	-0.5037
	H	-9.94347	-4.47447	0.98472
	C	0.03265	6.6778	-0.35643
	H	-0.85897	6.93649	-0.92893
	H	0.9346	6.91555	-0.92182
	H	0.03495	7.19772	0.60613
	C	10.1455	-3.627	-0.64578
	H	10.5149	-3.89377	0.3501
	H	10.9496	-3.20504	-1.25159
	H	9.72009	-4.51001	-1.13431

Compound	Coordinates			
	Atom	X	Y	Z
**C_H_PhCO2Me_Br**	I	1.9677	-0.29084	-0.38406
Img. Freq. = 0	I	-1.97335	-0.28682	-0.4605
SCF Energy= -4852.44555333	Br	0.01781	-2.68847	-1.58432
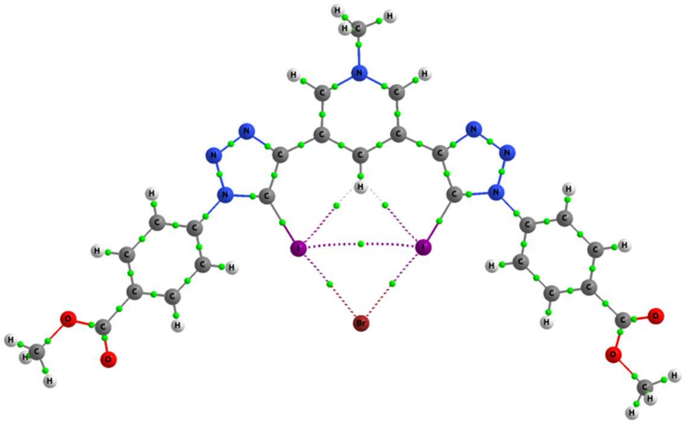	H	6.57635	1.47259	-0.50131
	H	4.05304	-0.83858	2.11432
	H	-8.3238	-0.28648	-0.70216
	H	8.30829	-0.31748	-0.4165
	H	5.77424	-2.64689	2.16992
	H	-4.14572	-0.85021	1.93916
	H	-6.58047	1.50879	-0.71795
	H	-5.86096	-2.64893	1.92286
	O	-7.95303	-3.69677	1.29997
	O	-9.29357	-2.51263	-0.05638
	N	4.60538	2.69329	1.08718
	N	4.25298	1.44179	0.74977
	N	-4.29329	1.44882	0.59822
	O	9.24214	-2.45042	0.24196
	N	-3.60426	3.45089	0.83774
	N	0.00066	5.233	-0.41539
	O	7.97572	-3.71532	1.59673
	N	-4.65327	2.70078	0.92595
	N	3.56203	3.44613	0.9623
	C	-0.01641	2.6531	0.51135
	H	-0.02402	1.65544	0.94695
	C	-8.26121	-2.62382	0.56821
	C	-1.22054	3.30525	0.23795
	C	-5.05373	-0.74299	1.3488
	C	2.9445	1.38424	0.39706
	C	-7.405	-0.40454	-0.13174
	C	-7.19516	-1.57526	0.60235
	C	-5.27537	0.41019	0.59949
	C	-1.17473	4.61594	-0.2299
	H	-2.06981	5.18808	-0.45873

	C	1.19971	3.30594	0.28136
	C	5.94199	-1.74518	1.58497
	C	7.37765	-0.4357	0.13282
	C	5.22908	0.3984	0.79161
	C	1.17062	4.61371	-0.18611
	H	2.07137	5.18981	-0.38437
	C	4.97871	-0.74372	1.54986
	C	6.4156	0.56893	0.08316
	C	7.13914	-1.59392	0.87874
	C	2.5068	2.69194	0.5441
	C	-2.97381	1.38838	0.28919
	C	-2.5377	2.69453	0.45366
	C	-6.02056	-1.7437	1.34253
	C	8.14221	-2.70073	0.95569
	C	-6.44054	0.59686	-0.14108
	C	-8.93925	-4.74041	1.30678
	H	-8.52802	-5.52883	1.93995
	H	-9.1024	-5.11128	0.28939
	H	-9.88084	-4.36631	1.72253
	C	0.03351	6.62066	-0.91707
	H	-0.98438	7.01028	-0.92767
	H	0.45307	6.6101	-1.92664
	H	0.66418	7.20942	-0.24708
	C	10.245	-3.47755	0.27672
	H	10.591	-3.63163	1.30429
	H	11.0603	-3.11293	-0.35089
	H	9.84059	-4.41287	-0.1245

Compound	Coordinates			
	Atom	X	Y	Z
**C_H_PhCO2Me_I**	I	1.94756	-0.10988	-0.68704
Img. Freq. = 0	I	-2.05248	-0.05386	-0.73405
SCF Energy= -2573.82758280	I	-0.07016	-2.58197	-2.19354
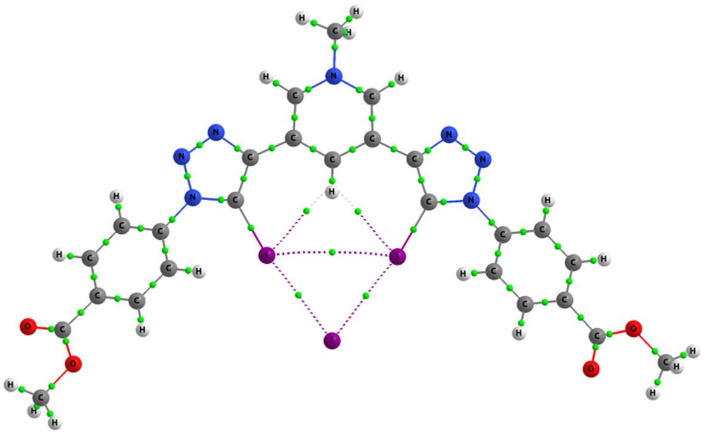	H	6.56486	1.59367	-0.615
	H	3.97578	-0.82209	1.83628
	H	-8.40436	-0.02108	-0.68334
	H	8.27803	-0.21718	-0.59465
	H	5.67851	-2.64952	1.82688
	H	-4.11698	-0.71799	1.7424
	H	-6.64839	1.76307	-0.70464
	H	-5.84659	-2.50323	1.73242
	O	-7.97842	-3.50375	1.17859
	O	-9.36318	-2.26571	-0.0819
	N	4.58272	2.74807	1.01386
	N	4.22276	1.52339	0.59695
	N	-4.30802	1.63588	0.51245
	O	9.17893	-2.38931	-0.03575
	N	-3.58964	3.6148	0.84249
	N	0.01717	5.41652	-0.40048

	O	7.88062	-3.71029	1.23263
	N	-4.64336	2.86989	0.92271
	N	3.54995	3.5197	0.91665
	C	-0.02692	2.79528	0.40295
	H	-0.04438	1.77767	0.79005
	C	-8.30658	-2.40579	0.4944
	C	-1.22375	3.47612	0.1713
	C	-5.04933	-0.5818	1.19762
	C	2.92016	1.50324	0.21978
	C	-7.46169	-0.16813	-0.16057
	C	-7.2295	-1.36817	0.5172
	C	-5.29558	0.60256	0.50695
	C	-1.16495	4.80652	-0.23503
	H	-2.05446	5.39965	-0.42984
	C	1.19528	3.44458	0.19902
	C	5.8645	-1.72112	1.29125
	C	7.33689	-0.35363	-0.06806
	C	5.1861	0.46745	0.59767
	C	1.18022	4.77302	-0.20563
	H	2.08747	5.34695	-0.37957
	C	4.91142	-0.70919	1.29179
	C	6.38566	0.66232	-0.08159
	C	7.0747	-1.54608	0.61321
	C	2.49412	2.80442	0.43692
	C	-3.00021	1.58231	0.15701
	C	-2.54451	2.87212	0.38125
	C	-6.02393	-1.57474	1.1956
	C	8.06625	-2.66543	0.64802
	C	-6.49033	0.82686	-0.17317
	C	-8.97313	-4.53915	1.196
	H	-8.54173	-5.35057	1.78524
	H	-9.18426	-4.87599	0.1756
	H	-9.89204	-4.17116	1.66464
	C	0.06556	6.82463	-0.83972
	H	-0.94602	7.22969	-0.81694
	H	0.46989	6.85318	-1.85518
	H	0.71533	7.37247	-0.15358
	C	10.1699	-3.42839	-0.04475
	H	10.5043	-3.6396	0.97649
	H	10.9953	-3.04117	-0.64511
	H	9.75901	-4.33696	-0.49745

Compound	Coordinates			
	Atom	X	Y	Z
**C_H_per_Cl**	I	1.93191	-0.2077	-0.68096
Img. Freq. = 0	I	-1.95507	-0.19587	-0.72168
SCF Energy= -3532.32415083	Cl	-0.00638	-2.32947	-1.94048
	F	6.48937	1.56955	-1.00719
	F	3.9276	-0.94351	2.05439

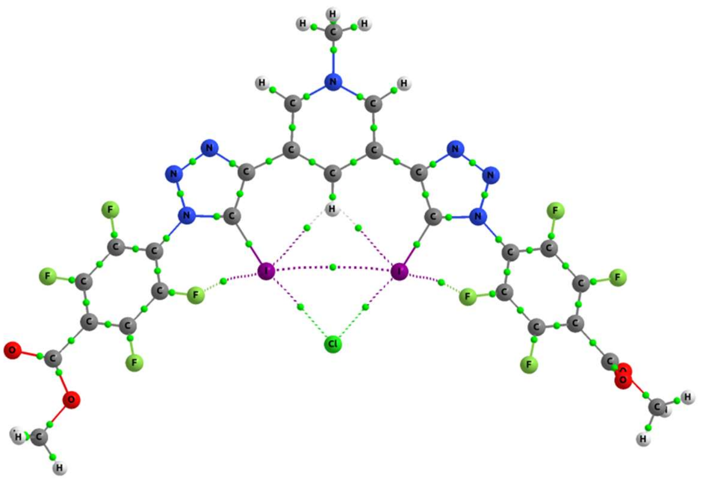

F	-8.3292	-0.34485	-1.26644
F	8.30549	-0.381	-1.10637
F	5.71299	-2.9286	1.92943
F	-4.01109	-0.89947	1.97271
F	-6.50438	1.5987	-1.15664
F	-5.80057	-2.87259	1.8412
O	-7.64005	-3.95422	0.10422
O	-9.35817	-2.50652	0.25351
N	4.59979	2.60675	1.06578
N	4.22799	1.40086	0.58845
N	-4.27222	1.43271	0.48298
O	8.46001	-3.125	-0.84989
N	-3.60927	3.40391	0.9175
N	0.00088	5.33313	-0.11124
O	8.4648	-3.38238	1.3872
N	-4.64821	2.6441	0.94334
N	3.56712	3.37412	1.01634
C	-0.01676	2.65975	0.48442
H	-0.02373	1.61512	0.78804
C	-8.17682	-2.74698	0.21405
C	-1.22229	3.34238	0.30233
C	-5.05809	-0.77256	1.15919
C	2.91868	1.3952	0.22368
C	-7.28092	-0.50576	-0.45921
C	-7.1237	-1.67727	0.27719
C	-5.21795	0.38615	0.40602
C	-1.17694	4.69982	0.00458
H	-2.06983	5.30159	-0.14368
C	1.19797	3.33346	0.33367
C	5.93315	-1.82967	1.20822
C	7.23025	-0.55673	-0.33735
C	5.17006	0.34993	0.52163
C	1.17062	4.69109	0.0343
H	2.07177	5.28569	-0.09214
C	4.9957	-0.81016	1.27008
C	6.2984	0.4697	-0.28236
C	7.06426	-1.72368	0.40304
C	2.50118	2.6831	0.51354
C	-2.95339	1.4158	0.15429
C	-2.53475	2.70235	0.44843
C	-5.99357	-1.79351	1.08211
C	8.07557	-2.83509	0.3854
C	-6.33753	0.50971	-0.4099
C	-8.58305	-5.04397	0.06907
H	-7.97714	-5.94858	-0.00174
H	-9.23453	-4.94475	-0.80484
H	-9.17952	-5.04849	0.98693
C	0.0092	6.7855	-0.37561

	H	-0.87716	7.03357	-0.96086
	H	0.91616	7.0289	-0.93051
	H	-0.00568	7.30794	0.58552
	C	9.45332	-4.16455	-0.95511
	H	10.3523	-3.87698	-0.4006
	H	9.66916	-4.25182	-2.02111
	H	9.05148	-5.10427	-0.56325

Compound	Coordinates			
	Atom	X	Y	Z
**C_H_per_Br**	I	1.95526	-0.12957	-0.61528
Img. Freq. = 0	I	-1.97935	-0.12413	-0.6439
SCF Energy= -5646.36642322	Br	-0.00562	-2.43846	-1.90331
	F	6.51356	1.65366	-0.9431
	F	3.91524	-0.77259	2.158
	F	-8.35378	-0.25454	-1.11863
	F	8.33023	-0.2987	-0.96531
	F	5.70041	-2.76027	2.10899
	F	-4.00024	-0.74305	2.0833
	F	-6.52628	1.68823	-1.073
	F	-5.7913	-2.7176	2.01281
	O	-7.64831	-3.83452	0.31146
	O	-9.36539	-2.3852	0.45787
	N	4.59539	2.75074	1.06387
	N	4.23063	1.52772	0.62681
	N	-4.27195	1.55323	0.53804
	O	8.47693	-3.03923	-0.62962
	N	-3.59927	3.53754	0.8908
	N	0.00037	5.41156	-0.28382
	O	8.46136	-3.2253	1.61445
	N	-4.63936	2.78164	0.9578
	N	3.56171	3.51372	0.97725
	C	-0.01491	2.77556	0.46497
	H	-0.02038	1.74969	0.82828
	C	-8.1845	-2.62585	0.40772
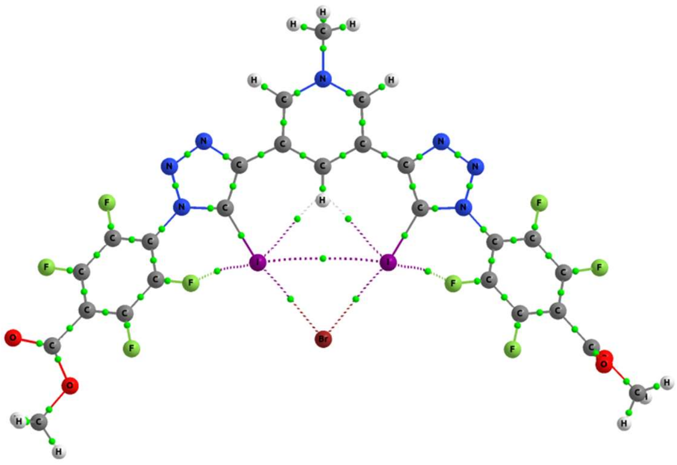	C	-1.22049	3.44707	0.24695
	C	-5.05588	-0.63267	1.27855
	C	2.92468	1.50652	0.25028
	C	-7.29659	-0.39894	-0.31994
	C	-7.13128	-1.55491	0.43869
	C	-5.22271	0.50911	0.50141
	C	-1.17646	4.78499	-0.12941
	H	-2.07045	5.37605	-0.31168
	C	1.19858	3.44116	0.27412
	C	5.92964	-1.68206	1.35978
	C	7.24573	-0.4527	-0.20465
	C	5.17548	0.4775	0.60375
	C	1.17032	4.77899	-0.10318
	H	2.07156	5.3648	-0.26632

	C	4.99218	-0.66106	1.38243
	C	6.31374	0.57493	-0.18941
	C	7.07047	-1.59854	0.56587
	C	2.50208	2.80251	0.48975
	C	-2.95732	1.52153	0.19313
	C	-2.53242	2.81657	0.43399
	C	-5.99242	-1.65455	1.2334
	C	8.08153	-2.70997	0.5923
	C	-6.35206	0.6164	-0.30346
	C	-8.59112	-4.92499	0.3039
	H	-7.98548	-5.83045	0.24209
	H	-9.25114	-4.84031	-0.56514
	H	-9.17861	-4.91445	1.22747
	C	0.00837	6.84646	-0.63005
	H	-0.88704	7.06455	-1.21335
	H	0.90685	7.05514	-1.21218
	H	0.0109	7.42237	0.30007
	C	9.46966	-4.08285	-0.69215
	H	10.3656	-3.77658	-0.14285
	H	9.69163	-4.20806	-1.7531
	H	9.06443	-5.00766	-0.26952

Compound	Coordinates			
	Atom	X	Y	Z
**C_H_per_I**	I	1.98287	-0.03419	-0.57351
Img. Freq. = 0	I	-2.00771	-0.02959	-0.60395
SCF Energy= -3367.74844975	I	-0.00525	-2.5815	-1.92566
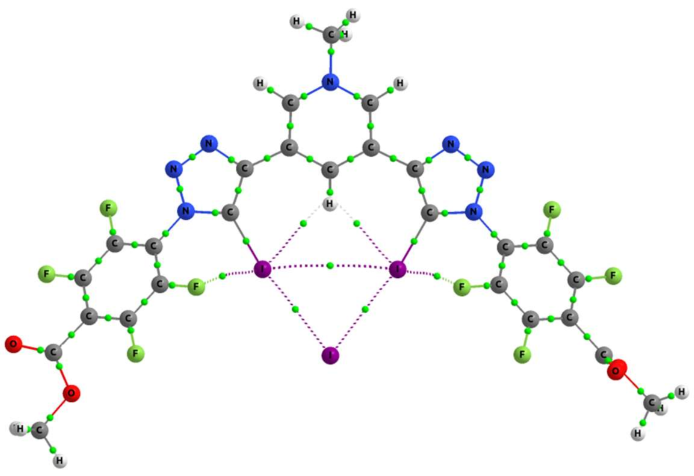	F	6.53966	1.7256	-0.88534
	F	3.88129	-0.56662	2.26589
	F	-8.38376	-0.17472	-0.93631
	F	8.35061	-0.22846	-0.7964
	F	5.6614	-2.5564	2.33227
	F	-3.96318	-0.55531	2.18696
	F	-6.55762	1.7697	-0.99381
	F	-5.7539	-2.53125	2.22077
	O	-7.64847	-3.70363	0.59159
	O	-9.36029	-2.24822	0.74189
	N	4.58648	2.90569	1.05181
	N	4.22921	1.66822	0.65188
	N	-4.26881	1.68759	0.57484
	O	8.50016	-2.94225	-0.34162
	N	-3.58638	3.68033	0.85497
	N	-0.00332	5.50161	-0.45554
	O	8.42274	-3.05403	1.90605
	N	-4.62614	2.92851	0.96434
	N	3.55365	3.66439	0.92382
	C	-0.01427	2.90298	0.41853
	H	-0.01821	1.89701	0.83561
	C	-8.18146	-2.49205	0.66634

	C	-1.21886	3.56286	0.17147
	C	-5.03581	-0.47216	1.40162
	C	2.92878	1.63347	0.25774
	C	-7.3096	-0.29187	-0.15611
	C	-7.12732	-1.42192	0.63662
	C	-5.22007	0.64334	0.5911
	C	-1.17768	4.88218	-0.27049
	H	-2.07458	5.45979	-0.47826
	C	1.19874	3.56135	0.19345
	C	5.90741	-1.51169	1.54217
	C	7.25314	-0.35034	-0.04884
	C	5.1722	0.61653	0.68531
	C	1.1683	4.87771	-0.24712
	H	2.06801	5.45774	-0.43906
	C	4.97233	-0.48905	1.50604
	C	6.32368	0.67893	-0.09189
	C	7.06248	-1.46302	0.76583
	C	2.50171	2.93569	0.44602
	C	-2.96083	1.64385	0.20664
	C	-2.5295	2.94395	0.39998
	C	-5.97205	-1.49512	1.41024
	C	8.07128	-2.57308	0.8573
	C	-6.36599	0.72383	-0.19327
	C	-8.5914	-4.79294	0.64103
	H	-7.98761	-5.70041	0.59181
	H	-9.27275	-4.73412	-0.21357
	H	-9.15589	-4.75372	1.57806
	C	0.02622	6.89895	-0.93053
	H	-0.99054	7.29141	-0.91776
	H	0.43041	6.90626	-1.94637
	H	0.66911	7.47215	-0.25873
	C	9.49417	-3.98638	-0.34311
	H	10.3743	-3.66214	0.22124
	H	9.74574	-4.14451	-1.39304
	H	9.07732	-4.89787	0.09682

Compound	Coordinates			
	Atom	X	Y	Z
**C_OH_PhCO2Me_Cl**	I	-1.91418	-0.43699	0.08908
Img. Freq. = 0	I	1.94725	-0.41832	0.1361
SCF Energy= -2888.89388578	Cl	0.02345	-2.86735	0.47124
	H	-6.21357	1.06542	1.54825
	H	-4.49448	-0.43111	-2.10582
	H	7.92731	-0.61214	1.75647
	H	-7.92499	-0.74964	1.48986
	H	-6.19051	-2.26702	-2.14759
	H	4.61825	-0.42163	-1.9566
	H	6.17556	1.17898	1.72595
	H	6.33956	-2.21812	-1.90974

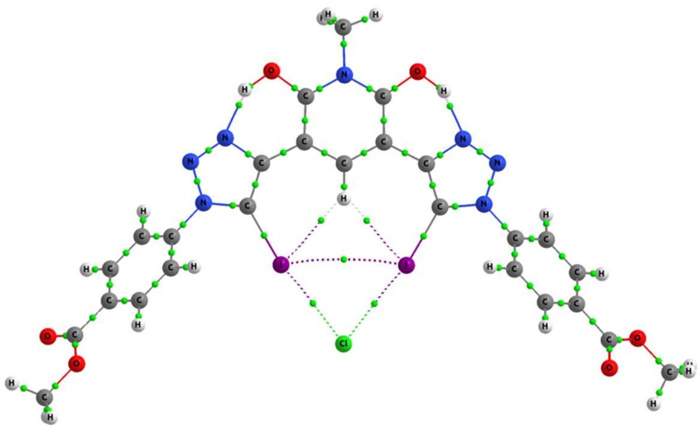

O	8.23393	-3.43163	-1.02461
O	9.12025	-2.64377	0.88146
N	-4.71989	2.71481	-0.27168
N	-4.30393	1.45033	-0.23332
N	4.32665	1.49514	-0.12724
O	-9.0291	-2.70536	0.59734
N	3.65728	3.49232	-0.15514
N	-0.01224	5.42107	0.11245
O	-8.16173	-3.56729	-1.28525
N	4.72638	2.76543	-0.16153
N	-3.66059	3.45517	-0.23311
C	0.00098	2.69737	-0.15825
H	0.00521	1.62023	-0.29579
C	8.29499	-2.5754	-0.00284
C	1.22127	3.36499	-0.07162
C	5.33582	-0.48165	-1.1402
C	-2.94876	1.36397	-0.15783
C	7.19777	-0.5663	0.95073
C	7.23429	-1.52196	-0.06825
C	5.31204	0.45449	-0.11023
C	1.17708	4.76533	0.05346
C	-1.22398	3.35914	-0.09546
C	-6.188	-1.53752	-1.34063
C	-7.17445	-0.6845	0.70618
C	-5.27152	0.39371	-0.26801
C	-1.1989	4.76225	0.03051
C	-5.23933	-0.52193	-1.31706
C	-6.22395	0.33212	0.74407
C	-7.15294	-1.62028	-0.33208
C	-2.53287	2.69186	-0.16629
C	2.97117	1.39155	-0.08708
C	2.53869	2.71363	-0.11302
C	6.30479	-1.48005	-1.11249
C	-8.15119	-2.73244	-0.40746
C	6.22893	0.43123	0.93699
C	9.22634	-4.46964	-1.01803
H	9.03215	-5.06675	-1.91092
H	9.12414	-5.08299	-0.11656
H	10.2296	-4.03258	-1.05913
C	0.02889	6.89108	0.24575
H	0.63467	7.13841	1.11975
H	-0.98455	7.2608	0.37125
H	0.48187	7.31022	-0.65647
C	-10.012	-3.75224	0.58407
H	-10.6079	-3.69839	-0.33303
H	-10.6398	-3.57624	1.45951
H	-9.52219	-4.72932	0.65329
O	-2.26788	5.52196	0.06427

	H	-3.07879	4.92416	-0.05477
	O	2.22873	5.54791	0.1106
	H	3.05229	4.96703	0.01033

Compound	Coordinates			
	Atom	X	Y	Z
**C_OH_PhCO2Me_Br**	I	1.92908	-0.59858	-0.21477
Img. Freq. = 0	I	-1.97038	-0.58096	-0.23834
SCF Energy= -5002.93607793	Br	-0.03036	-3.18997	-0.74169
	H	6.23336	0.95303	-1.57687
	H	4.47558	-0.64758	2.01413
	H	-7.95624	-0.7628	-1.73194
	H	7.94506	-0.86237	-1.55143
	H	6.17267	-2.48332	2.02247
	H	-4.59216	-0.59899	1.93238
	H	-6.20825	1.03292	-1.71044
	H	-6.3099	-2.40009	1.8946
	O	-8.21385	-3.61101	1.02388
	O	-9.13077	-2.80506	-0.85996
	N	4.71668	2.54862	0.27333
	N	4.3033	1.28542	0.19445
	N	-4.33092	1.33306	0.12023
	O	9.03698	-2.84564	-0.70689
	N	-3.65821	3.32787	0.18585
	N	0.00938	5.25984	-0.06433
	O	8.15543	-3.75635	1.14596
	N	-4.72828	2.60227	0.19013
	N	3.65657	3.28833	0.24795
	C	-0.00338	2.52963	0.14091
	H	-0.0073	1.44922	0.25346
	C	-8.29248	-2.744	0.01252
	C	-1.22355	3.19986	0.07389
	C	-5.32224	-0.65372	1.12666
	C	2.94879	1.19989	0.1052
	C	-7.2148	-0.72273	-0.93683
	C	-7.23361	-1.68835	0.0732
	C	-5.3161	0.29209	0.10529
	C	-1.17966	4.6029	-0.01732
	C	1.22119	3.19341	0.09225
	C	6.1783	-1.73109	1.23663
	C	7.186	-0.81988	-0.77446
	C	5.27201	0.22931	0.20918
	C	1.19594	4.59931	0.00088
	C	5.22924	-0.71564	1.23151
	C	6.23534	0.19715	-0.79383
	C	7.15389	-1.78491	0.23638
	C	2.53042	2.52592	0.14996
	C	-2.97631	1.22916	0.05844
	C	-2.54121	2.54924	0.1105

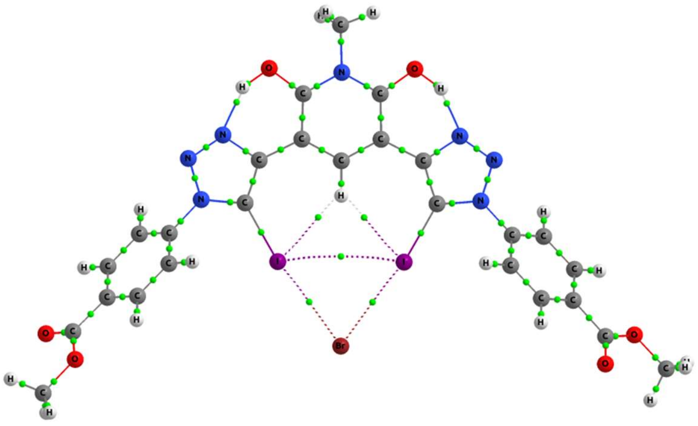

C	-6.2889	-1.65427	1.10408
C	8.15164	-2.89879	0.2903
C	-6.2483	0.27731	-0.92818
C	-9.20339	-4.65177	1.02061
H	-8.99418	-5.25821	1.90376
H	-9.11303	-5.25454	0.11081
H	-10.2071	-4.21812	1.08154
C	-0.03178	6.73273	-0.16109
H	-0.6439	7.00159	-1.0242
H	0.98094	7.10496	-0.28506
H	-0.47777	7.12982	0.75454
C	10.0196	-3.89281	-0.71427
H	10.6098	-3.86285	0.20765
H	10.6531	-3.6944	-1.58081
H	9.53015	-4.86763	-0.81182
O	2.26452	5.36006	-0.01345
H	3.07531	4.76059	0.09731
O	-2.23123	5.38681	-0.05044
H	-3.05439	4.80432	0.04455

Compound	Coordinates			
	Atom	X	Y	Z
**C_OH_PhCO2Me_I**	I	1.95595	-0.45245	-0.066
Img. Freq. = 0	I	-1.98829	-0.43245	-0.14386
SCF Energy= -2724.31787212	I	-0.02145	-3.30615	-0.51474
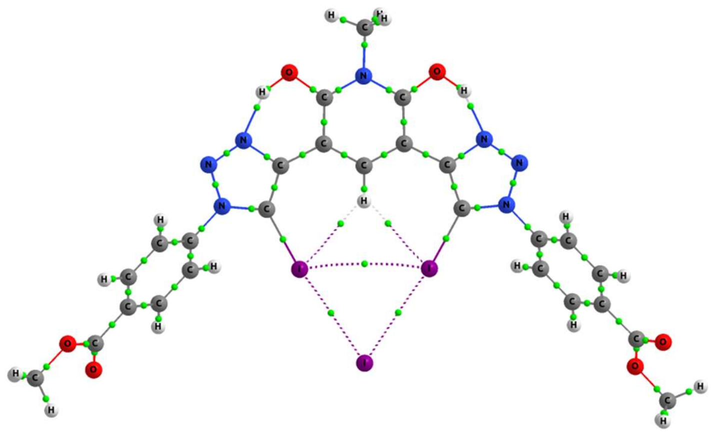	H	6.18325	1.04545	-1.56594
	H	4.54378	-0.38066	2.15188
	H	-7.89454	-0.66272	-1.79629
	H	7.89699	-0.76753	-1.50949
	H	6.24485	-2.2137	2.19485
	H	-4.6623	-0.35064	1.97568
	H	-6.14872	1.13546	-1.78265
	H	-6.37795	-2.15507	1.94765
	O	-8.24179	-3.4101	1.0498
	O	-9.09728	-2.67191	-0.88978
	N	4.7205	2.72761	0.28296
	N	4.31053	1.46208	0.24797
	N	-4.33464	1.50646	0.09834
	O	9.00703	-2.7188	-0.61612
	N	-3.65707	3.49981	0.11949
	N	0.0123	5.4257	-0.08852
	O	8.20917	-3.51885	1.32365
	N	-4.72924	2.77742	0.12513
	N	3.65798	3.46284	0.23901
	C	-0.00194	2.69666	0.13812
	H	-0.00679	1.61631	0.2547
	C	-8.28918	-2.57789	0.00786
	C	-1.22155	3.3665	0.05581
	C	-5.36445	-0.4378	1.14826

	C	2.95574	1.37046	0.17014
	C	-7.18178	-0.59059	-0.97759
	C	-7.23566	-1.51666	0.0676
	C	-5.32194	0.46678	0.09114
	C	-1.17698	4.76907	-0.04768
	C	1.22292	3.36066	0.09687
	C	6.22383	-1.50007	1.37407
	C	7.16367	-0.68754	-0.71109
	C	5.28239	0.40865	0.28359
	C	1.19838	4.7661	-0.00845
	C	5.27355	-0.48592	1.3509
	C	6.21209	0.32817	-0.74787
	C	7.16577	-1.60255	0.34571
	C	2.53276	2.69564	0.17118
	C	-2.97928	1.39824	0.06463
	C	-2.54039	2.71764	0.08563
	C	-6.32918	-1.44033	1.13014
	C	8.16651	-2.71272	0.42042
	C	-6.21657	0.41076	-0.9735
	C	-9.22675	-4.4553	1.04763
	H	-9.04336	-5.03303	1.95541
	H	-9.1052	-5.08535	0.16022
	H	-10.2336	-4.02483	1.06333
	C	-0.02849	6.89775	-0.19797
	H	-0.6271	7.15933	-1.07275
	H	0.98572	7.26999	-0.30846
	H	-0.48935	7.30141	0.70733
	C	9.99268	-3.76312	-0.60319
	H	10.6229	-3.67529	0.28804
	H	10.5861	-3.61746	-1.50769
	H	9.50367	-4.74291	-0.61864
	O	2.26621	5.52699	-0.02481
	H	3.07831	4.92692	0.08387
	O	-2.2274	5.55277	-0.10069
	H	-3.05281	4.96994	-0.0189

Compound	Coordinates			
	Atom	X	Y	Z
**C_OH_per_Cl**	I	-1.91423	-0.58608	-0.05458
Img. Freq. = 0	I	1.93213	-0.58374	-0.01119
SCF Energy= -3682.81366956	Cl	0.00908	-3.02466	0.01835
	F	-5.87523	0.84002	2.12422
	F	-4.65452	-0.36302	-2.26535
	F	7.65521	-1.12257	2.34368
	F	-7.67932	-1.13162	2.17801
	F	-6.43805	-2.3602	-2.22409
	F	4.71562	-0.35192	-2.15367
	F	5.85127	0.85128	2.2586
	F	6.49876	-2.34379	-2.08376

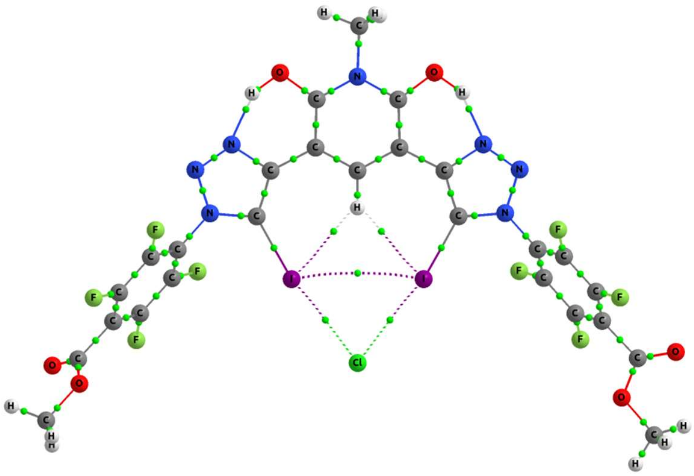

O	7.70264	-4.03358	-0.26963
O	9.2797	-2.70461	0.63485
N	-4.71767	2.594	-0.10034
N	-4.29103	1.32732	-0.09413
N	4.30669	1.33519	0.0101
O	-8.09694	-3.6298	1.07804
N	3.66929	3.33615	-0.01581
N	0.00576	5.30518	-0.16608
O	-8.88128	-3.10094	-0.96599
N	4.72848	2.60391	0.01763
N	-3.66025	3.32946	-0.10971
C	0.00448	2.57058	-0.08039
H	0.00252	1.48566	-0.0434
C	8.1696	-2.88528	0.19949
C	1.22791	3.23661	-0.08407
C	5.42987	-0.55048	-1.04799
C	-2.93107	1.24038	-0.09805
C	6.95451	-0.9491	1.2238
C	7.13188	-1.79978	0.13588
C	5.25327	0.28305	0.05057
C	1.19149	4.64182	-0.12495
C	-1.21648	3.24004	-0.11614
C	-6.32728	-1.58275	-1.1478
C	-6.94075	-0.97722	1.07907
C	-5.23531	0.27208	-0.07165
C	-1.1844	4.64794	-0.15557
C	-5.39689	-0.55538	-1.17739
C	-6.01647	0.05758	1.05752
C	-7.11277	-1.81249	-0.02094
C	-2.52489	2.56909	-0.10902
C	2.94754	1.24364	-0.02732
C	2.53743	2.57064	-0.04496
C	6.3513	-1.58564	-0.99726
C	-8.13615	-2.91368	-0.03649
C	6.02099	0.07631	1.1907
C	8.64545	-5.12432	-0.27098
H	8.10757	-5.97108	-0.70012
H	8.96101	-5.34451	0.75368
H	9.51245	-4.86428	-0.88646
C	0.0554	6.7809	-0.21206
H	0.52943	7.1403	0.70461
H	-0.95734	7.16431	-0.29288
H	0.64544	7.07687	-1.08208
C	-9.06454	-4.69582	1.15776
H	-10.0769	-4.28459	1.09267
H	-8.89989	-5.16367	2.12972
H	-8.89479	-5.41332	0.34892
O	-2.24849	5.4149	-0.181

	H	-3.06553	4.82116	-0.15789
	O	2.24547	5.42364	-0.12548
	H	3.06738	4.84127	-0.08066

Compound	Coordinates			
	Atom	X	Y	Z
**C_OH_per_Br**	I	-1.93296	-0.46853	-0.05074
Img. Freq. = 0	I	1.94982	-0.46612	-0.00918
SCF Energy= -5796.85581742	Br	0.00794	-3.09114	0.02523
	F	-5.87984	0.96872	2.1241
	F	-4.66381	-0.22902	-2.26809
	F	7.66367	-0.98637	2.3406
	F	-7.68838	-0.99924	2.1751
	F	-6.45216	-2.22223	-2.2299
	F	4.72429	-0.22045	-2.15769
	F	5.85461	0.98298	2.25578
	F	6.51273	-2.20787	-2.08817
	O	7.72001	-3.89558	-0.27422
	O	9.29342	-2.56267	0.63088
	N	-4.71909	2.72353	-0.10136
	N	-4.29533	1.45621	-0.0941
	N	4.30994	1.46421	0.00691
	O	-8.11096	-3.49527	1.07006
	N	3.66799	3.46338	-0.01959
	N	0.0051	5.42913	-0.16446
	O	-8.89681	-2.95916	-0.97152
	N	4.72876	2.73358	0.01312
	N	-3.66016	3.45664	-0.11015
	C	0.00386	2.69364	-0.08121
	H	0.00193	1.60837	-0.04526
	C	8.18382	-2.74626	0.19548
	C	1.22712	3.36023	-0.08495
	C	5.43883	-0.41777	-1.05199
	C	-2.93536	1.3668	-0.09668
	C	6.96302	-0.81404	1.22053
	C	7.14319	-1.66349	0.13213
	C	5.25937	0.41443	0.04712
	C	1.19069	4.76572	-0.12472
	C	-1.21694	3.36371	-0.11555
	C	-6.33906	-1.447	-1.15228
	C	-6.94991	-0.84435	1.07617
	C	-5.24231	0.40318	-0.07304
	C	-1.1849	4.77188	-0.15395
	C	-5.40644	-0.42167	-1.18038
	C	-6.02334	0.18847	1.05615
	C	-7.12431	-1.67712	-0.02533
	C	-2.52602	2.69425	-0.10839
	C	2.95075	1.37009	-0.0288
	C	2.53736	2.69577	-0.0469

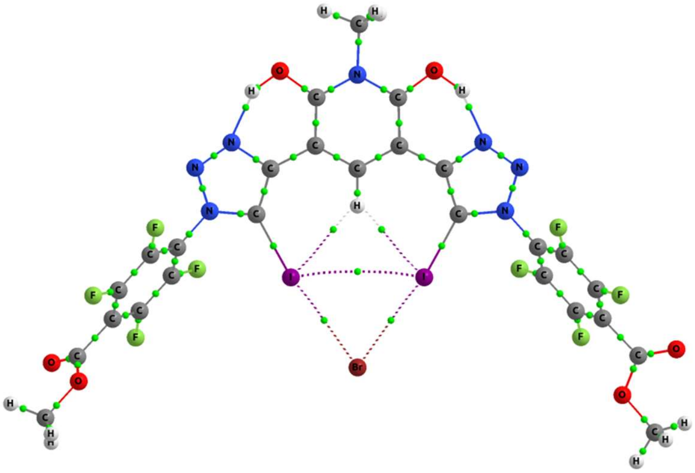

	C	6.36281	-1.45064	-1.0014
	C	-8.15014	-2.77604	-0.04245
	C	6.02688	0.20902	1.1876
	C	8.66591	-4.98366	-0.27636
	H	8.13037	-5.83166	-0.70598
	H	8.9822	-5.20361	0.74813
	H	9.53208	-4.72077	-0.89178
	C	0.05477	6.90492	-0.20905
	H	0.53008	7.26327	0.70737
	H	-0.95804	7.28849	-0.28809
	H	0.64371	7.20171	-1.07952
	C	-9.08049	-4.55969	1.14796
	H	-10.0922	-4.14636	1.08541
	H	-8.91543	-5.03071	2.11832
	H	-8.91314	-5.27512	0.33679
	O	-2.24857	5.53913	-0.17846
	H	-3.06609	4.94541	-0.15634
	O	2.24426	5.54778	-0.12555
	H	3.06666	4.96534	-0.08247

Compound	Coordinates			
	Atom	X	Y	Z
**C_OH_per_I**	I	-1.95682	-0.34247	-0.04899
Img. Freq. = 0	I	1.97244	-0.3397	-0.00324
SCF Energy= -3518.23746891	I	0.00804	-3.2035	0.0432
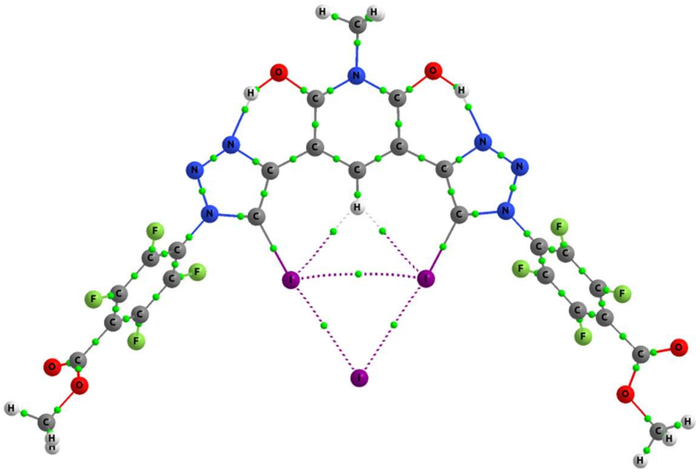	F	-5.88756	1.11171	2.11708
	F	-4.67514	-0.08697	-2.27569
	F	7.67871	-0.83723	2.33115
	F	-7.70212	-0.85094	2.16691
	F	-6.47	-2.07465	-2.23902
	F	4.73319	-0.07719	-2.1641
	F	5.86287	1.12626	2.24935
	F	6.52898	-2.05849	-2.09795
	O	7.74329	-3.74366	-0.28618
	O	9.31284	-2.40587	0.61847
	N	-4.72109	2.86354	-0.10937
	N	-4.30136	1.59537	-0.10059
	N	4.31481	1.60397	0.00217
	O	-8.13181	-3.34513	1.0603
	N	3.66652	3.60068	-0.02464
	N	0.00418	5.56221	-0.15574
	O	-8.91641	-2.8054	-0.98082
	N	4.72947	2.87424	0.0063
	N	-3.66011	3.59345	-0.11647
	C	0.0032	2.82549	-0.08445
	H	0.00142	1.7397	-0.05401
	C	8.20352	-2.59313	0.18396
	C	1.22624	3.49281	-0.08458
	C	5.44975	-0.27296	-1.05946

	C	-2.94141	1.50281	-0.10026
	C	6.97665	-0.66624	1.21182
	C	7.15899	-1.51394	0.12242
	C	5.26809	0.55734	0.04073
	C	1.18967	4.89879	-0.1187
	C	-1.21754	3.49616	-0.1163
	C	-6.35456	-1.30045	-1.16099
	C	-6.96327	-0.69753	1.0681
	C	-5.25199	0.54535	-0.08037
	C	-1.1856	4.90483	-0.14922
	C	-5.41883	-0.27795	-1.18844
	C	-6.03357	0.33252	1.04879
	C	-7.14021	-1.529	-0.03398
	C	-2.52752	2.82847	-0.11213
	C	2.9556	1.5065	-0.03003
	C	2.53751	2.83032	-0.04887
	C	6.37729	-1.30269	-1.0105
	C	-8.16915	-2.62506	-0.05169
	C	6.03702	0.35371	1.18056
	C	8.69302	-4.82842	-0.29011
	H	8.15997	-5.67802	-0.71966
	H	9.01127	-5.04789	0.73386
	H	9.55753	-4.56208	-0.90638
	C	0.05389	7.03825	-0.19385
	H	0.53357	7.39207	0.722
	H	-0.95916	7.4224	-0.26622
	H	0.63891	7.33901	-1.06563
	C	-9.10415	-4.40706	1.13747
	H	-10.1147	-3.99105	1.07498
	H	-8.94046	-4.87905	2.10758
	H	-8.93853	-5.12245	0.32591
	O	-2.24864	5.67245	-0.17254
	H	-3.06683	5.07874	-0.15529
	O	2.24248	5.68134	-0.11685
	H	3.06572	5.09894	-0.07888

Compound	Coordinates			
	Atom	X	Y	Z
**D_OH_per_Cl**	I	1.90922	-0.23273	-0.89416
Img. Freq. = 0	I	-1.93369	-0.22142	-0.9295
SCF Energy: -3611.78272309	Cl	-0.00644	-2.15501	-2.259
	F	6.51436	1.35381	-0.8251
	F	3.67691	-1.11146	2.01544
	F	-8.27081	-0.65379	-1.02455
	F	8.2235	-0.7051	-0.92573
	F	5.36804	-3.19093	1.91592
	F	-3.73973	-1.12465	1.92818
	F	-6.55011	1.39614	-0.89137
	F	-5.43531	-3.1935	1.79013

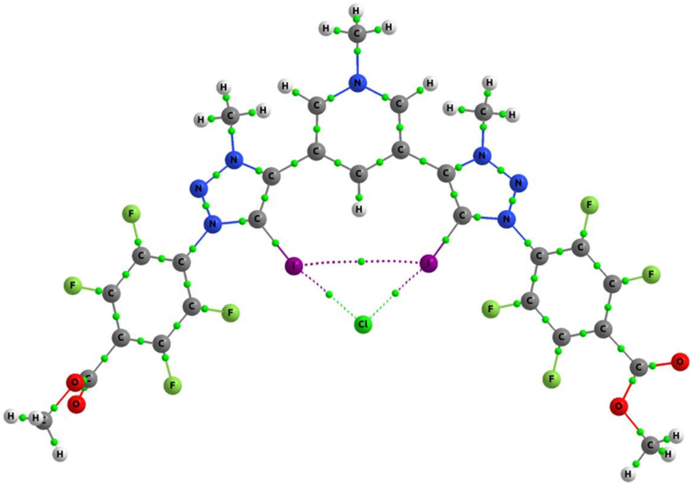

O	-7.30114	-4.27234	0.04983
O	-9.08139	-2.95193	0.45313
N	4.53538	2.36607	1.23168
N	4.16712	1.2418	0.64721
N	-4.2102	1.2505	0.58805
O	8.19819	-3.4697	-0.74578
N	-3.55921	3.15905	1.06951
N	-0.00828	5.02208	-0.80994
O	8.12836	-3.75553	1.48781
N	-4.58274	2.37215	1.17491
N	3.51521	3.15486	1.10842
C	-0.0192	2.60668	0.50301
H	-0.02373	1.66777	1.0565
C	-7.89684	-3.11661	0.30166
C	-1.21983	3.22424	0.14777
C	-4.84555	-1.01927	1.19713
C	2.90338	1.29249	0.14136
C	-7.17163	-0.79224	-0.28695
C	-6.9034	-1.98874	0.37209
C	-5.12341	0.16332	0.52226
C	-1.183	4.44379	-0.51423
H	-2.07817	4.97287	-0.83381
C	1.18722	3.22038	0.16067
C	5.67746	-2.08446	1.24449
C	7.11079	-0.83794	-0.2063
C	5.07625	0.14984	0.6009
C	1.16152	4.43955	-0.50256
H	2.06142	4.96569	-0.81377
C	4.79371	-1.0179	1.29992
C	6.23935	0.24073	-0.15372
C	6.84569	-2.01309	0.49036
C	2.47269	2.56816	0.45432
C	-2.94011	1.30139	0.09791
C	-2.51034	2.57405	0.42412
C	-5.72742	-2.08619	1.11086
C	7.79792	-3.17822	0.48243
C	-6.28887	0.27586	-0.22663
C	-8.17982	-5.41449	-0.01585
H	-7.52945	-6.26806	-0.21285
H	-8.90146	-5.2812	-0.82787
H	-8.70147	-5.53643	0.93865
C	-0.00264	6.34327	-1.4751
H	-0.89857	6.41959	-2.09261
H	0.89564	6.41292	-2.09008
H	-0.00088	7.11201	-0.69664
C	9.13121	-4.56566	-0.84358
H	10.033	-4.33742	-0.26674
H	9.36412	-4.65296	-1.90589

	H	8.66545	-5.48372	-0.4719
	C	-3.63338	4.51061	1.63323
	H	-2.69066	4.71213	2.14754
	H	-3.80082	5.21969	0.81836
	H	-4.46963	4.51783	2.33341
	C	3.58631	4.51125	1.6611
	H	3.77692	5.21109	0.84331
	H	2.63421	4.72474	2.15281
	H	4.40714	4.51949	2.37937

Compound	Coordinates			
	Atom	X	Y	Z
**D_OH_per_Br**	I	1.94381	-0.20988	-0.79749
Img. Freq. = 0	I	-1.96948	-0.1987	-0.84608
SCF Energy: -5725.82469362	Br	-0.00303	-2.33558	-2.18087
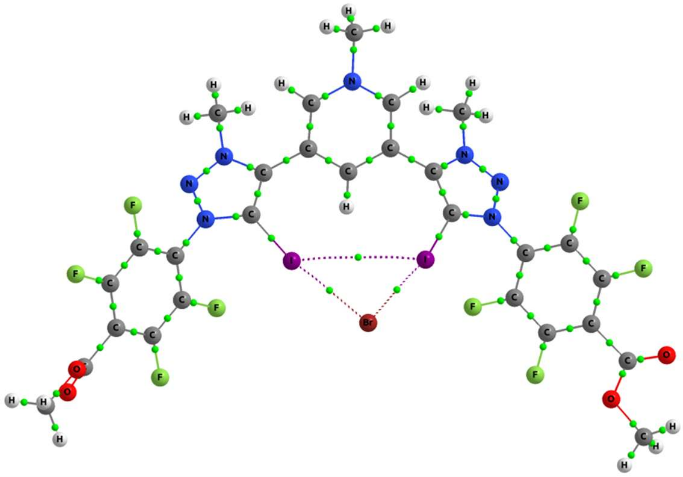	F	6.52691	1.4605	-0.75244
	F	3.71697	-0.98029	2.1362
	F	-8.33987	-0.49402	-0.85616
	F	8.28145	-0.56275	-0.77689
	F	5.45321	-3.02604	2.11044
	F	-3.77129	-1.00138	2.03272
	F	-6.56872	1.51479	-0.8043
	F	-5.51594	-3.03213	1.97193
	O	-7.44101	-4.10719	0.29014
	O	-9.17971	-2.73291	0.69573
	N	4.52911	2.49651	1.24198
	N	4.17478	1.34557	0.70264
	N	-4.21344	1.35544	0.64005
	O	8.30468	-3.32768	-0.51612
	N	-3.53871	3.27624	1.03337
	N	-0.00345	5.04678	-0.94234
	O	8.24246	-3.52928	1.727
	N	-4.56723	2.50388	1.18546
	N	3.50553	3.27241	1.0745
	C	-0.01546	2.6795	0.45551
	H	-0.02068	1.75827	1.03837

	C	-8.00288	-2.93134	0.52555
	C	-1.21508	3.28557	0.07984
	C	-4.88567	-0.88762	1.31626
	C	2.91601	1.36627	0.1823
	C	-7.23172	-0.64136	-0.1339
	C	-6.9806	-1.82738	0.55035
	C	-5.14741	0.28425	0.61701
	C	-1.17874	4.48003	-0.62675
	H	-2.07367	4.99962	-0.96254
	C	1.1907	3.2837	0.09755
	C	5.74464	-1.9326	1.41007
	C	7.16493	-0.69813	-0.06393
	C	5.10211	0.26816	0.69672
	C	1.1663	4.47769	-0.61015
	H	2.06641	4.99574	-0.93454
	C	4.8375	-0.8842	1.42715
	C	6.26978	0.36183	-0.05092
	C	6.91823	-1.85755	0.66496
	C	2.47514	2.65156	0.43302
	C	-2.94988	1.3748	0.13052
	C	-2.50561	2.65588	0.39611
	C	-5.79432	-1.9348	1.27102
	C	7.89779	-2.99936	0.70057
	C	-6.32253	0.40561	-0.11559
	C	-8.34853	-5.22832	0.2689
	H	-7.72357	-6.10222	0.07889
	H	-9.08321	-5.09564	-0.53144
	H	-8.85305	-5.31542	1.23633
	C	0.00438	6.34511	-1.65206
	H	-0.89479	6.40539	-2.26663
	H	0.90001	6.38953	-2.27341
	H	0.01419	7.13945	-0.89984
	C	9.26684	-4.40125	-0.57268
	H	10.1623	-4.12645	-0.00636
	H	9.50155	-4.52339	-1.63114
	H	8.82613	-5.31636	-0.1649
	C	-3.59232	4.65189	1.53924
	H	-2.62882	4.87907	2.00186
	H	-3.79842	5.32207	0.70062
	H	-4.39702	4.68972	2.27462
	C	3.56187	4.65176	1.56996
	H	3.78629	5.31349	0.72936
	H	2.59303	4.89006	2.01546
	H	4.35499	4.68953	2.31783

Compound	Coordinates			
	Atom	X	Y	Z
**D_OH_per_I**	I	1.96911	-0.13161	-0.7305

Img. Freq. = 0

SCF Energy: -3447.20601836

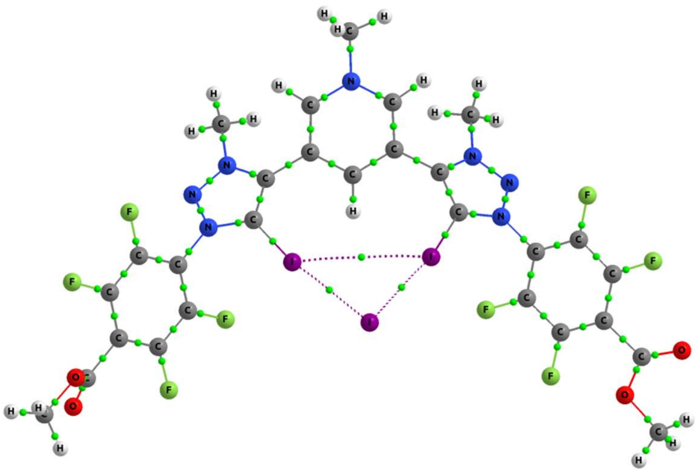

I	-1.99986	-0.12907	-0.78676
I	0.00313	-2.49524	-2.17452
F	6.52474	1.53847	-0.67646
F	3.68995	-0.79457	2.27563
F	-8.33781	-0.4405	-0.71519
F	8.2788	-0.48349	-0.61263
F	5.42597	-2.83912	2.34117
F	-3.75404	-0.78702	2.17273
F	-6.57266	1.57341	-0.77354
F	-5.49332	-2.82223	2.22735
O	-7.42691	-3.98854	0.62507
O	-9.16568	-2.59495	0.95802
N	4.50895	2.64509	1.26688
N	4.16143	1.47718	0.76143
N	-4.20959	1.4929	0.66728
O	8.30557	-3.23379	-0.24859
N	-3.53201	3.42895	0.96869
N	0.00293	5.11366	-1.08265
O	8.21635	-3.3583	1.99923
N	-4.55811	2.66367	1.16565
N	3.49091	3.417	1.05406
C	-0.01849	2.80419	0.40579
H	-0.02704	1.90888	1.02841
C	-7.98934	-2.80155	0.79383
C	-1.2143	3.39413	-0.00073
C	-4.87348	-0.71308	1.45908
C	2.91266	1.48324	0.21788
C	-7.22562	-0.5485	0.00765
C	-6.96863	-1.69688	0.75159
C	-5.14191	0.42059	0.70223
C	-1.17401	4.55995	-0.75672
H	-2.0682	5.06256	-1.11967
C	1.19165	3.39588	0.03603
C	5.72396	-1.77297	1.60257
C	7.15643	-0.59367	0.09526
C	5.08828	0.4001	0.80403
C	1.17171	4.56042	-0.71606
H	2.07292	5.07124	-1.05102
C	4.81697	-0.7246	1.57383
C	6.26142	0.46592	0.06227
C	6.90362	-1.72566	0.86453
C	2.47057	2.777	0.41483
C	-2.95244	1.49121	0.14273
C	-2.50517	2.78204	0.34687
C	-5.779	-1.76381	1.47183
C	7.88355	-2.86454	0.95105
C	-6.31975	0.50084	-0.03134
C	-8.33051	-5.1112	0.68909

	H	-7.70513	-5.99374	0.54659
	H	-9.07771	-5.03281	-0.10668
	H	-8.81984	-5.13687	1.66789
	C	0.04216	6.34812	-1.89756
	H	-0.97504	6.72369	-2.00274
	H	0.46676	6.09453	-2.8724
	H	0.67251	7.07334	-1.37825
	C	9.27328	-4.3037	-0.25691
	H	10.1601	-4.00533	0.31124
	H	9.52192	-4.45977	-1.30774
	H	8.83184	-5.20701	0.17563
	C	-3.58311	4.82387	1.41767
	H	-2.61385	5.0721	1.85654
	H	-3.80383	5.45862	0.55565
	H	-4.3768	4.88986	2.16303
	C	3.54399	4.81146	1.50399
	H	3.78429	5.44459	0.64606
	H	2.56861	5.06637	1.92503
	H	4.32448	4.87195	2.26362

Compound	Coordinates			
	Atom	X	Y	Z
**D_OH_PhCO2Me_Cl**	I	1.90438	-0.44235	-0.8835
Img. Freq. = 0	I	-1.97027	-0.40586	-0.92088
SCF Energy: -2817.87075300	Cl	-0.03658	-2.34718	-2.29511
	H	6.58696	1.08857	-0.39571
	H	3.7669	-1.38024	1.73628
	H	-8.30408	-0.64797	-0.54826
	H	8.22813	-0.78982	-0.40285
	H	5.40347	-3.27004	1.6996
	H	-3.89417	-1.33329	1.66124
	H	-6.627	1.21352	-0.49215
	H	-5.55019	-3.19016	1.57422
	O	-7.64909	-4.24599	0.9948
	O	-9.14682	-2.96174	-0.07644
	N	4.53127	2.164	1.22881
	N	4.17125	1.02587	0.67568
	N	-4.23192	1.0905	0.61818
	O	9.01877	-3.02394	0.07711
	N	-3.54234	3.00329	1.05374
	N	0.00666	4.7913	-0.87177
	O	7.59049	-4.37519	1.16113
	N	-4.57839	2.22919	1.17854
	N	3.5102	2.95445	1.08517
	C	-0.01815	2.39671	0.47678
	H	-0.0288	1.4659	1.04347
	C	-8.05832	-3.10785	0.43389
	C	-1.21541	3.01673	0.11571

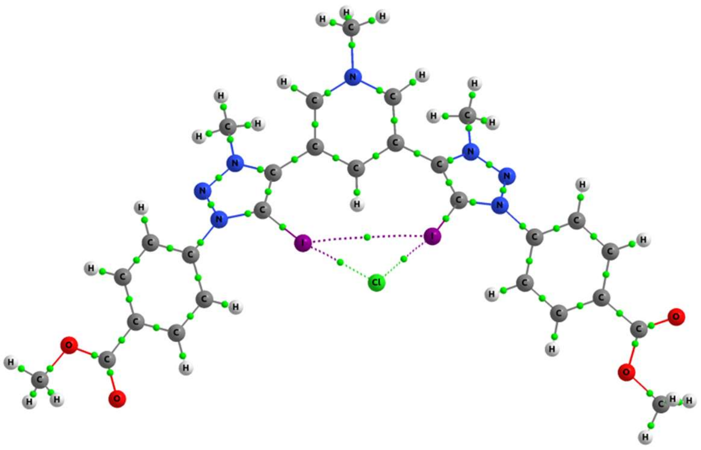

	C	-4.85412	-1.2001	1.1654
	C	2.91007	1.06664	0.16695
	C	-7.33696	-0.7904	-0.0713
	C	-7.0248	-2.02585	0.50236
	C	-5.18841	0.01586	0.57935
	C	-1.17166	4.2256	-0.56545
	H	-2.06367	4.75719	-0.88953
	C	1.19225	2.99774	0.12665
	C	5.65367	-2.31624	1.24057
	C	7.25404	-0.92187	0.06095
	C	5.10689	-0.0675	0.66321
	C	1.17336	4.20651	-0.55582
	H	2.07605	4.72406	-0.87329
	C	4.73799	-1.27037	1.2571
	C	6.34501	0.13218	0.06373
	C	6.90667	-2.14323	0.64563
	C	2.47407	2.3503	0.44325
	C	-2.96467	1.11435	0.12365
	C	-2.51002	2.38776	0.41677
	C	-5.78709	-2.23172	1.11962
	C	7.85466	-3.30283	0.66434
	C	-6.41189	0.24713	-0.04017
	C	-8.5977	-5.32456	0.9605
	H	-8.10525	-6.16388	1.45479
	H	-8.84117	-5.57805	-0.07665
	H	-9.50822	-5.04145	1.49898
	C	0.02024	6.10445	-1.55229
	H	-0.87786	6.18183	-2.16649
	H	0.91631	6.15976	-2.17204
	H	0.03209	6.88227	-0.78297
	C	9.9769	-4.09448	0.06937
	H	10.2266	-4.38236	1.09599
	H	10.8557	-3.69735	-0.4419
	H	9.57389	-4.9567	-0.47212
	C	3.58005	4.32196	1.60664
	H	3.80275	5.00208	0.78032
	H	2.6176	4.55998	2.06594
	H	4.3789	4.34013	2.34927
	C	-3.59518	4.36693	1.58712
	H	-2.63547	4.58346	2.06252
	H	-3.79347	5.05843	0.764
	H	-4.4045	4.39279	2.31805

Compound	Coordinates			
	Atom	X	Y	Z
**D_OH_PhCO2Me_Br**	I	-1.92637	-0.36197	0.76379
Img. Freq. = 0	I	1.99346	-0.32499	0.83168
SCF Energy: -4931.91282187	Br	0.02944	-2.46889	2.2199

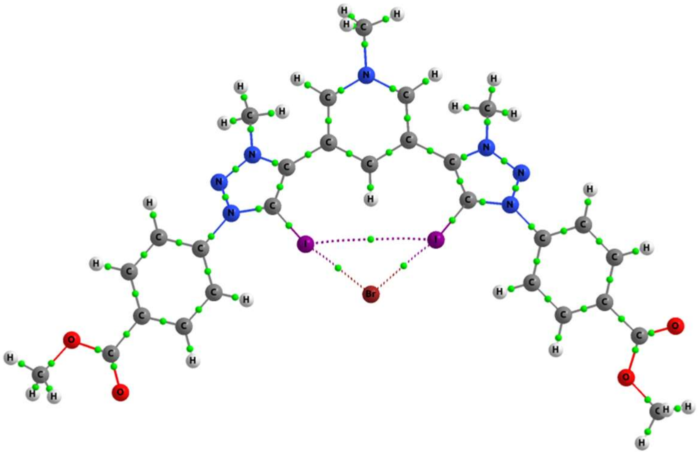

H	-6.58563	1.20232	0.27286
H	-3.75079	-1.20391	-1.91114
H	8.32149	-0.54524	0.34205
H	-8.22569	-0.67566	0.21555
H	-5.38704	-3.09332	-1.94026
H	3.86433	-1.1439	-1.79556
H	6.64998	1.32001	0.38778
H	5.51564	-3.00501	-1.80922
O	7.62304	-4.08533	-1.31305
O	9.14113	-2.84925	-0.21398
N	-4.51263	2.32793	-1.28688
N	-4.16	1.16892	-0.77449
N	4.22855	1.24063	-0.67133
O	-9.00719	-2.89846	-0.31937
N	3.53524	3.17228	-1.0079
N	-0.01861	4.87117	0.98803
O	-7.57669	-4.21612	-1.44135
N	4.56882	2.40433	-1.1817
N	-3.49568	3.11351	-1.09436
C	0.02226	2.52952	-0.44645
H	0.03956	1.6188	-1.04517
C	8.04534	-2.97112	-0.71498
C	1.21407	3.13778	-0.05446
C	4.83469	-1.02913	-1.31604
C	-2.90762	1.19142	-0.24362
C	7.34404	-0.66884	-0.11889
C	7.01593	-1.88309	-0.72778
C	5.18419	0.16466	-0.69403
C	1.16292	4.32105	0.67342
H	2.05227	4.83975	1.02457
C	-1.19408	3.11867	-0.09152
C	-5.6408	-2.15321	-1.45569
C	-7.24882	-0.79386	-0.2461
C	-5.09776	0.07758	-0.80896
C	-1.18307	4.29983	0.63442
H	-2.08804	4.80975	0.95993
C	-4.72525	-1.10723	-1.43612
C	-6.34025	0.25993	-0.21287
C	-6.89695	-1.99825	-0.86243
C	-2.46967	2.48558	-0.45861
C	2.96797	1.24307	-0.15941
C	2.51056	2.52851	-0.38614
C	5.76521	-2.06375	-1.32639
C	-7.84289	-3.15858	-0.91479
C	6.42163	0.37123	-0.09376
C	8.56621	-5.16898	-1.33214
H	8.06336	-5.9859	-1.8528
H	8.82029	-5.46399	-0.3086

	H	9.47184	-4.86955	-1.87003
	C	-0.06821	6.1259	1.76992
	H	0.95228	6.46917	1.93578
	H	-0.56597	5.91182	2.71907
	H	-0.63578	6.85961	1.19265
	C	-9.96267	-3.97119	-0.33873
	H	-10.2133	-4.23244	-1.37224
	H	-10.8417	-3.5899	0.18414
	H	-9.55668	-4.84635	0.17929
	C	-3.56103	4.4998	-1.56419
	H	-3.81052	5.14578	-0.71845
	H	-2.58813	4.76094	-1.98732
	H	-4.33928	4.5421	-2.32739
	C	3.58327	4.5582	-1.481
	H	2.61417	4.79975	-1.92414
	H	3.80416	5.2112	-0.63264
	H	4.37542	4.61311	-2.22894

Compound	Coordinates			
	Atom	X	Y	Z
**D_OH_PhCO2Me_I**	I	-1.95263	-0.25832	0.70503
Img. Freq. = 0	I	2.02445	-0.2301	0.76588
SCF Energy: -2653.29432096	I	0.02928	-2.59476	2.2414
	H	-6.59907	1.30911	0.15507
	H	-3.70615	-1.0021	-2.05428
	H	8.31005	-0.47083	0.14657
	H	-8.2369	-0.56447	-0.02839
	H	-5.33756	-2.8897	-2.2051
	H	3.83498	-0.91204	-1.9917
	H	6.6474	1.39598	0.32235
	H	5.4766	-2.77714	-2.13493
	O	7.58132	-3.89616	-1.72324
	O	9.11974	-2.73295	-0.57391
	N	-4.49549	2.49535	-1.31093
	N	-4.1486	1.3207	-0.83239
	N	4.21878	1.39356	-0.72516
	O	-9.00777	-2.75449	-0.69337
	N	3.52602	3.33927	-0.96832
	N	-0.02252	4.95461	1.11426
	O	-7.53467	-4.03759	-1.79986
	N	4.55442	2.57673	-1.19047
	N	-3.48449	3.27653	-1.07463
	C	0.02248	2.67275	-0.41323
	H	0.04103	1.78756	-1.04973
	C	8.0161	-2.8217	-1.06447
	C	1.21314	3.26503	0.00612
	C	4.80831	-0.83423	-1.511
	C	-2.90575	1.32878	-0.27949
	C	7.32821	-0.56102	-0.31274

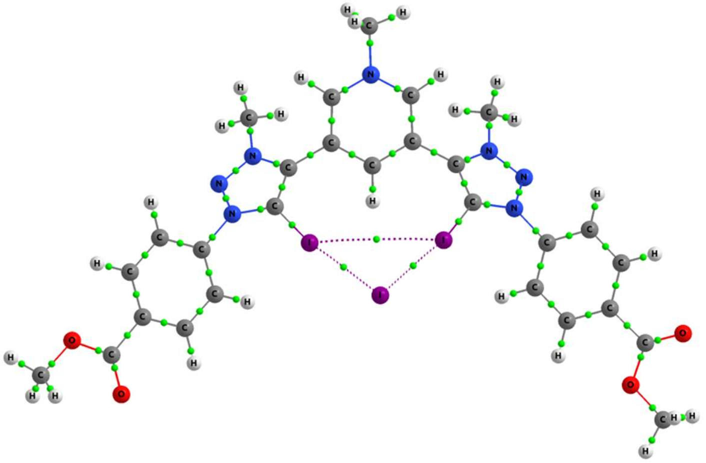

C	6.98996	-1.73252	-0.99556
C	5.1684	0.31574	-0.81707
C	1.16024	4.41631	0.78346
H	2.04856	4.91923	1.15972
C	-1.19473	3.2488	-0.04093
C	-5.6051	-1.97141	-1.68718
C	-7.24682	-0.66352	-0.46577
C	-5.08161	0.22917	-0.9308
C	-1.18547	4.40053	0.73082
H	-2.09128	4.9011	1.0684
C	-4.69147	-0.92738	-1.59845
C	-6.33998	0.38715	-0.36185
C	-6.87722	-1.84084	-1.12284
C	-2.4671	2.62997	-0.44349
C	2.96687	1.37779	-0.19365
C	2.50852	2.67197	-0.35886
C	5.73384	-1.86997	-1.59439
C	-7.82088	-2.99715	-1.2503
C	6.41085	0.47932	-0.21437
C	8.52227	-4.97672	-1.82859
H	8.00728	-5.76022	-2.38744
H	8.7981	-5.33396	-0.83094
H	9.41651	-4.64502	-2.36662
C	-0.07665	6.17777	1.94418
H	0.94082	6.53598	2.09673
H	-0.54524	5.919	2.89708
H	-0.67344	6.9201	1.40924
C	-9.95996	-3.8268	-0.78148
H	-10.1697	-4.0577	-1.83117
H	-10.8592	-3.46173	-0.28208
H	-9.57237	-4.71633	-0.2739
C	-3.54811	4.67741	-1.4981
H	-3.81278	5.29363	-0.63498
H	-2.56982	4.95516	-1.89702
H	-4.31432	4.7433	-2.27179
C	3.57431	4.74409	-1.381
H	2.60101	5.00764	-1.80128
H	3.80826	5.35876	-0.50788
H	4.35708	4.82902	-2.13607

Figure S1. Molecular electrostatic potential on the 0.001 au electron density isosurface for selected compounds at m062x/aug-cc-pVDZ computational level.

A_H_per	A_OH_mes
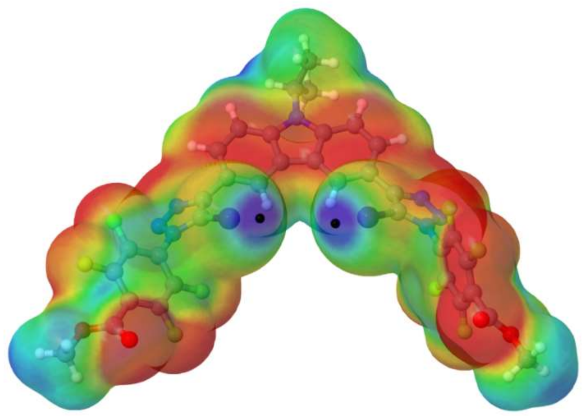	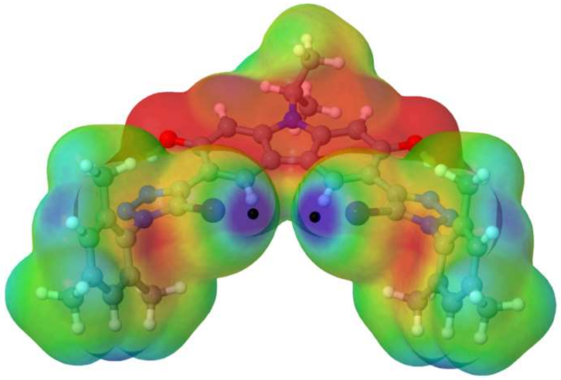

Table S2. Equilibrium constants $k = \exp\left\{\frac{\{\Delta G(X^-)\}}{RT}\right\}$ for the halogen-bonding interaction for complexes **A** and **B**.

	K		
**Complexes**	**Cl**	**Br**	**I**
**A_H_mes**	2.46E+02	8.58E+01	3.39E+01
**A_H_per**	2.25E+04	1.66E+04	2.66E+03
**A_OH_mes**	1.32E+03	1.26E+03	6.11E+02
**A_OH_per**	1.44E+06	2.25E+05	1.78E+05
**B_H_mes**	4.55E+13	8.55E+13	2.35E+14
**B_H_per**	4.73E+16	1.58E+16	1.53E+15

Figure S2. Molecular electrostatic potential on the 0.001 au electron density isosurface for selected compounds at M062X/aug-cc-pVDZ computational level.

$C\_H\_PhCO_2Me$	C_H_per
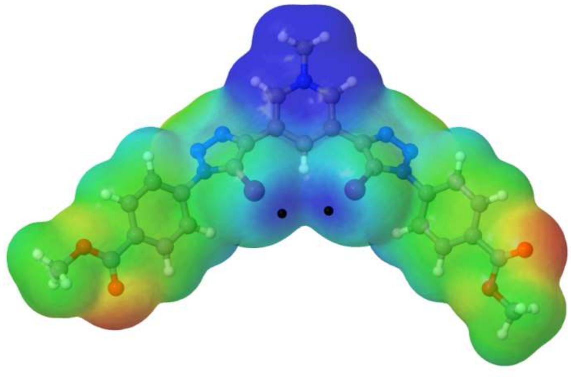	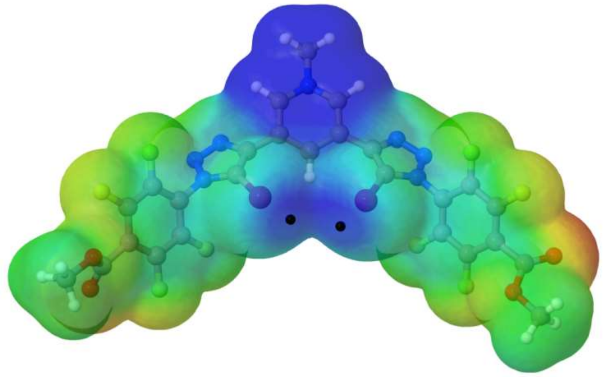
$C\_OH\_PhCO_2Me$	C_OH_per

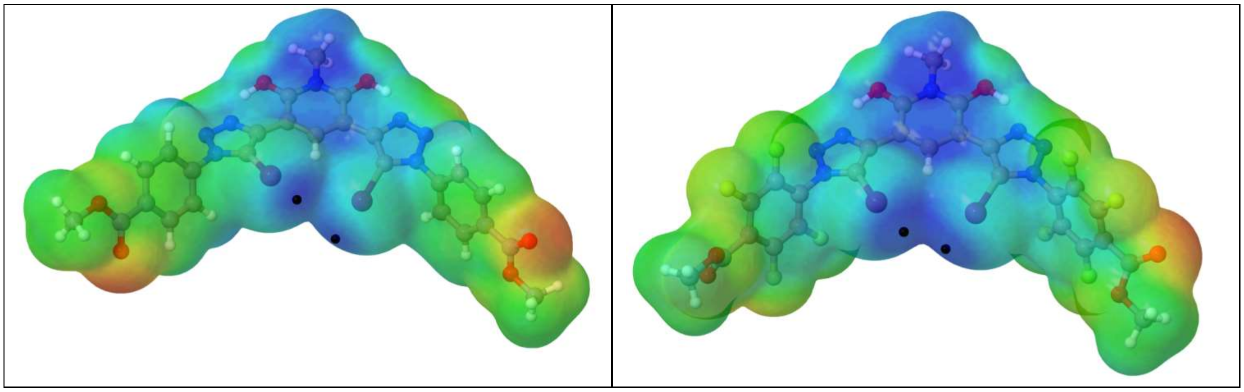

Table S3. Maxima ($V_{max,X}$) values of the molecular electrostatic potential (in a.u.) over the 0.001 e^- electron density isosurface for all the monomers (**C**) calculated at MP2/aug-cc-pVDZ level.

Monomers	1	2
**C_H_PhCO$_2$Me**	0.1505	0.1529
**C_H_per**	0.1511	0.1509
**C_OH_PhCO$_2$Me**	0.1675	0.1675
**C_OH_per**	0.1620	0.1622

Table S4. Equilibrium constants $k = \exp\left\{\frac{\{\Delta G(X^-)\}}{RT}\right\}$ for the halogen-bonding interaction for complexes **C** and **D**.

	K		
**Complexes**	**Cl**	**Br**	**I**
**C_H_PhCO$_2$Me**	1.11E+05	6.65E+04	8.64E+04
**C_H_per**	8.24E+05	1.10E+06	2.29E+05
**C_OH_PhCO$_2$Me**	9.15E+04	1.24E+05	8.74E+05
**C_OH_per**	1.65E+06	9.31E+05	6.14E+05
**D_H_PhCO$_2$Me**	4.20E+08	3.24E+09	5.05E+08
**D_H_per**	2.06E+09	4.86E+09	8.96E+08

Figure S3. Relationship between density at BCPs and bond distances (X···I) for all the families under study.

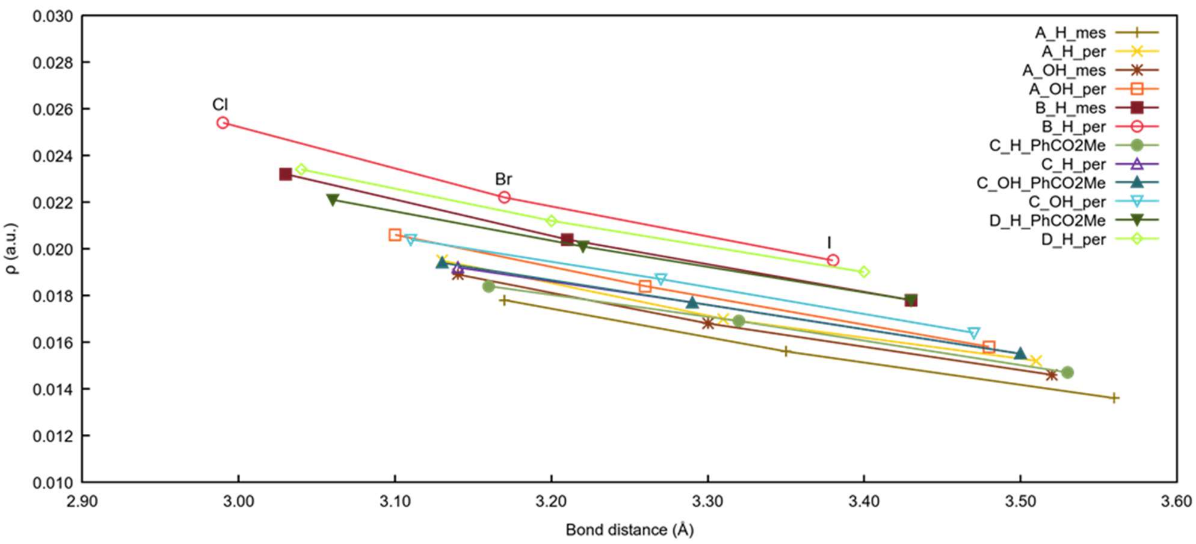

A_H_mes
A_H_per
A_OH_mes
A_OH_per
B_H_mes
B_H_per
C_H_PhCO2Me
C_H_per
C_OH_PhCO2Me
C_OH_per
D_H_PhCO2Me
D_H_per
Cl
Br
I
ρ (a.u.)
Bond distance (Å)
0.030
0.028
0.026
0.024
0.022
0.020
0.018
0.016
0.014
0.012
0.010
2.90
3.00
3.10
3.20
3.30
3.40
3.50
3.60
